# Donor-Acceptor Derivatives of Indolo[3,2-b]indole and Benzothieno[3,2-b]benzothiophene: Similar Annulated Structures but Divergent Properties

**DOI:** 10.3390/molecules31122046

**Published:** 2026-06-11

**Authors:** Liya A. Poletavkina, Ivan V. Dyadishchev, Artem V. Bakirov, Evgenia A. Svidchenko, Nikolay M. Surin, Nikita O. Dubinets, Dmitry O. Balakirev, Svetlana M. Peregudova, George V. Cherkaev, Irina A. Chuyko, Sergei N. Chvalun, Yuriy N. Luponosov

**Affiliations:** 1 Enikolopov Institute of Synthetic Polymeric Materials, Russian Academy of Sciences, Profsoyuznaya St. 70, Moscow 117393, Russia; l.poletavkina@ispm.ru (L.A.P.); dyadischev_iv@ispm.ru (I.V.D.); bakirov.artem@gmail.com (A.V.B.); svidchenko@ispm.ru (E.A.S.); surinnm@ispm.ru (N.M.S.); nikita.dubinets@gmail.com (N.O.D.); balakirev@ispm.ru (D.O.B.); smp@ineos.ac.ru (S.M.P.); georgij.cherkaev@gmail.com (G.V.C.); chuyko@ispm.ru (I.A.C.); s-chvalun@yandex.ru (S.N.C.); 2 Federal Research Center for Problems of Chemical Physics and Medicinal Chemistry, Russian Academy of Sciences, Semenov Ave. 1, Chernogolovka, Moscow 142432, Russia; 3 National Research Center “Kurchatov Institute”, Kurchatov Sq. 1, Moscow 123182, Russia; 4 Nesmeyanov Institute of Organoelement Compounds of the Russian Academy of Sciences, Vavilova St. 28, Moscow 119991, Russia

**Keywords:** annulated molecular structures, π-conjugated oligomers, push-pull molecules, optical properties, organic semiconductors

## Abstract

Annulated organic molecular structures with planar, fused backbones exhibit superior properties compared to non-fused systems, including high crystallinity, strong π–π stacking, and excellent charge transport characteristics. The rational design of annulated compounds with targeted characteristics presents a significant challenge that requires a comprehensive understanding of structure–property relationships. This work addresses this by synthesizing a series of novel push–pull systems featuring benzothieno[3,2-b]benzothiophene (BT) or its nitrogen-rich analogue, indolo[3,2-b]indole (ID), as electron-donating units, connected via a phenylene π-spacer to two distinct electron-accepting groups (carbonyl or dicyanovinyl). The thermal, structural, optical and electrochemical properties of these compounds were thoroughly investigated. Computational studies of the optical and electrochemical properties, including those of unsubstituted ID and BT model cores, showed excellent agreement with experimental data, validating the theoretical models. Notably, ID-based derivatives exhibited remarkably high photoluminescence quantum yield and enhanced solubility compared to their BT counterparts, along with thermal properties that are more favorable for device fabrication. This work provides the first systematic comparison of these annulated cores, offering novel structure–property insights that may support the rational design of organic functional materials and contribute to the further development of organic electronics.

## 1. Introduction

Small-molecule organic semiconductors represent a promising class of materials for organic electronic applications, including organic light-emitting diodes (OLEDs), organic field-effect transistors (OFETs) and organic and perovskite solar cells [1,2,3,4,5,6,7]. The feasibility of their use, as well as the ultimate device performance, depend critically on the physicochemical properties of the materials, which are governed by the molecular structure. Molecular structure directly influences key material parameters such as charge carrier mobility [8,9], optical properties [10,11], thin film morphology and solid-state molecular packing [7,8,12,13]. Therefore, elucidating the relationships between molecular structure and physicochemical properties of organic semiconductors is both of fundamental interest and essential for the rational design and efficient fabrication of organic electronic devices [2,14].

A broad spectrum of synthetic organic chemistry techniques can be employed to produce materials with the desired characteristics. The most commonly used current synthetic strategies for upgrading organic semiconductors include: extending π-conjugated systems to improve intermolecular interactions and charge mobility [15]; introducing electron acceptor (A) and electron donor (D) groups to regulate molecular orbital energies (HOMO and LUMO) and change the width of the bandgap [16,17]; functionalizing side chains with alkyl radicals to enhance compound solubility and film morphology [18]; and heteroatomic substitution with atoms such as sulfur, nitrogen, and oxygen, which influences electron density and molecular packing [19].

Among annulated heteroatom-containing donor blocks, benzothieno[3,2-b]benzothiophene (BT) is a well-known compound [20]. Its structure consists of two fused thiophene rings connected to two benzene rings, forming a rigid π-conjugated system. This architecture enables dense and efficient molecular packing, allowing BT derivatives to exhibit high hole mobility and enhanced stability due to their relatively low HOMO energy levels [21,22]. In this case, the sulfur atoms are involved in non-covalent interactions (S···S and S···π), which improve molecular packing. The sulfur unshared electron pairs delocalize into the π-system, lowering the energy barrier for charge transfer [23]. Functionalization of BT derivatives is typically achieved at the lateral positions of the benzene rings, with the 2,7-positions being the most commonly utilized sites.

Indole[3,2-b]indole (ID) is a nitrogen-containing analogue of BT, featuring a rigid, planar structure composed of two fused indole units. The nitrogen atoms in the pyrrole fragments contribute to the aromatic sextet by donating their unshared pair electrons, resulting in a more electron-rich π-system compared to sulfur in thiophene. This leads to higher HOMO energy levels and improved p-type conductivity [24,25]. Additionally, nitrogen participates in non-covalent hydrogen bonding interactions (C-H···N), which promote denser and more ordered molecular packing within the block, significantly influencing charge mobility [26]. Another notable advantage of the ID structure is the presence of functionalizable positions on the nitrogen heteroatoms, thereby expanding the opportunities for structural modification [27]. Despite the extensive development of BT- and ID-based compounds, studies specifically addressing their push-pull derivatives remain relatively scarce. Consequently, the systematic modulation of the optical and electrochemical properties of BT and ID molecular systems through the incorporation of electron-donating and electron-accepting groups continue to represent a comparatively underexplored research area. Moreover, direct comparative analyses between these two analogues are virtually absent from the literature, hindering a comprehensive understanding of their relative advantages and limitations. Addressing these gaps is of both scientific and practical importance for researchers in organic electronics and materials science. Such investigations will not only deepen our fundamental understanding of structure–property relationships in organic semiconductors but also inform the rational design of next-generation high-performance organic electronic materials.

**Figure 1:**
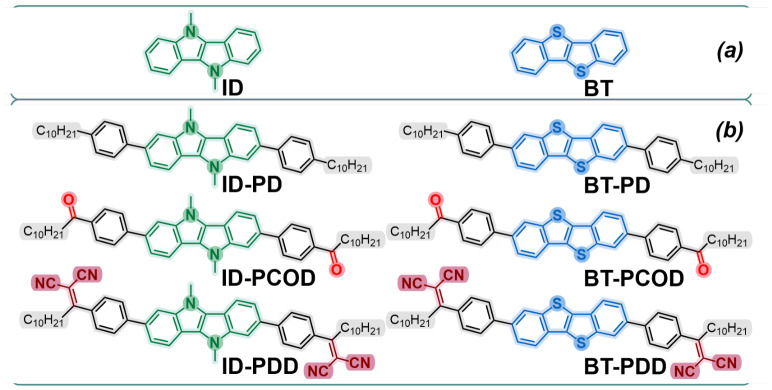
Chemical structures of the model compounds (**a**) and push-pull molecules synthesized in this work (**b**).

In this work, we report the design and synthesis of a series of novel molecules with ID and BT cores connected via a benzene π-spacer to either alkyl or various electron-withdrawing groups (EWGs) (Figure 1). To enable a more detailed analysis of the optical and electrochemical properties of the synthesized molecules, two model compounds were also prepared (see ID and BT, Figure 1a). A comprehensive study of their optical and electrochemical properties, phase behavior, thermal stability, and solubility can provide deep insights into the structural factors governing their physicochemical properties, thereby elucidating the underlying structure–property relationships.

## 2. Results and Discussion

### 2.1. Synthesis

**Figure 2:**
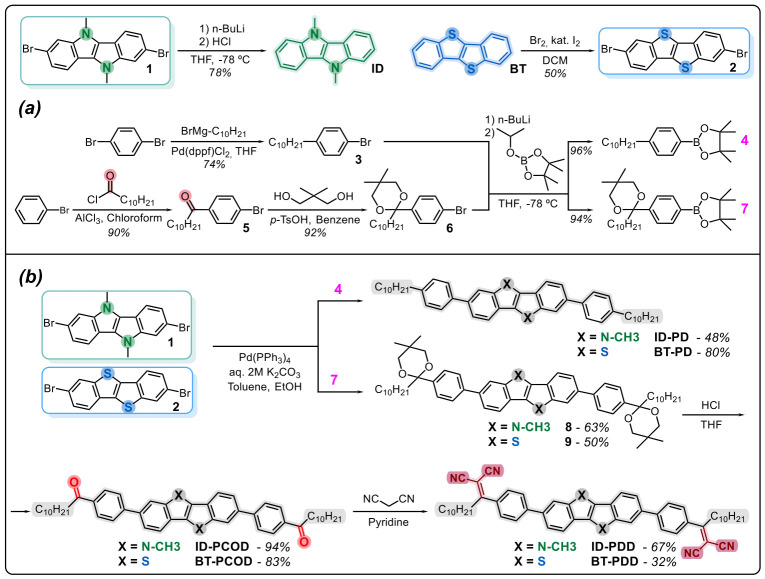
The synthesis scheme of key precursors (**a**) and target molecules (**b**).

The synthetic strategy for the target molecules is outlined in Figure 2 and includes the preparation of several key precursors, namely, 2,7-dibromo-5,10-dimethyl-5,10-dihydroindolo[3,2-b]indole (**1**), 2,7-dibromo[1]benzothieno [3,2-b][1]benzothiophene (**2**), 2-(4-decylphenyl)-4,4,5,5-tetramethyl-1,3,2-dioxaborolane (**4**) and 2-decyl-5,5-dimethyl-2-[4-(4,4,5,5-tetramethyl-1,3,2-dioxaborolan-2-yl)phenyl]-1,3-dioxane (**7**), followed by Suzuki cross-coupling reaction between them.

The target compound ID-PD and precursor **1** were previously obtained and described by the present authors [28]. The target compound BT-PD was first obtained by Fedorenko et al. [29], and subsequently by the present authors [30].

The non-methylated precursor of compound **1** was prepared using a well-known method [31]. The model compound 5,10-dimethyl-5,10-dihydroindolo[3,2-b]indole (ID) was obtained by lithiation of compound 1 followed by addition of hydrochloric acid, the yield was 78%. A dibromo-functional derivative of benzothieno[3,2-b]benzothiophene (compound **2**) was prepared in accordance with a published method [32], whilst the model compound BT was obtained during its synthesis. The organoboronic precursor **4** was synthesized according to a previously described procedure [29], which involves the Kumada reaction between 1,4-dibromobenzene and n-dodecylmagnesium bromide (**3**) in 74% yield. Then, a lithium derivative of compound **6** was obtained and subsequently reacted with 2-isopropoxy-4,4,5,5,5-tetramethyl-1,3,2-dioxaborolan (IPTMDOB). This process yielded precursor **4** with a yield of 96%. To synthesize precursor **7**, bromobenzene (**5**) was first formylated with a yield of 90%. Then, the carbonyl group was protected using 2,2-dimethylpropane-1,3-diol with a yield of 92%. Finally, compound **6** was lithiated with n-BuLi and reacted with IPTMDOB. The yield at the final stage of obtaining precursor **7** was 95%.

Suzuki cross-coupling reactions between compounds **1** and **2** and the organoboron precursor **4** produced the target ID-PD and BT-PD products in yields of 48% and 80%, respectively (Figure 2b). Suzuki cross-coupling reactions between **1** or **2** and precursor **7** produced acetals **8** and **9** in yields of 63% and 50%, respectively. The protective groups were removed via acid hydrolysis of the acetals using 1 M HCl, yielding the target molecules ID-PCOD and BT-PCOD with carbonyl EWGs in high yields. Finally, target molecules ID-PDD and BT-PDD with terminal EWGs based on dicyanovinyl were synthesized via Knöevenagel condensation of aldehydes with malononitrile, yielding 32–67%.

The chemical structure and purity of the target compounds were confirmed by NMR spectroscopy, GPC, elemental analysis and MALDI-TOF mass spectrometry (see Experimental Part in Supporting Information, Figures S1–S28).

### 2.2. Optical Properties

The compounds obtained belong to the class of heterocyclic compounds. The general features of the electronic absorption and luminescence spectra of organic compounds are discussed in detail in many works [33,34]. It is well-known that in the ground state of heterocyclic compounds, the unshared pair of electrons on the heteroatom participate in conjugation with the π-electrons of the molecule. Therefore, in the absorption spectra of such compounds, in addition to high-intensity (if there is no symmetry ban ε ≥ 10^3^ L mol^−1^ cm^−1^) absorption bands resulting from π→π* transitions, there are bands attributable to n→π* transitions. The absorption bands due to n→π* transitions are low-intensity even if such transitions are symmetry-resolved (ε ≤ 10^3^ L mol^−1^ cm^−1^). As a consequence, nπ* bands are typically obscured under intense ππ* bands. The fluorescence properties of molecules with n,π*-states are contingent on the relative positioning of the lowest excited singlet and triplet ππ* and nπ* energy levels. The presence of nπ*-states with energies lower than the energy of the long-wavelength π→π*-transition itself can lead to suppression of π*→π-fluorescence. The presence of a large number of closely spaced heteroatoms may lead to an increase in the number of n→π* transitions, which may result in an increase in the probability of S_ππ*_ − T_nπ*_ conversion.

In heteroaromatic systems, the relative energetic alignment and coupling of the n→π* and π→π* excited states are highly sensitive to the identity, number, and spatial arrangement of heteroatoms within the molecular framework. Consequently, even compounds possessing closely related substitution patterns may exhibit substantially different absorption and fluorescence characteristics, as replacement of heteroatoms (for example, nitrogen by sulfur) can profoundly modify the electronic structure of the donor core, the distribution of excited-state electron density, and the efficiency of non-radiative S_ππ*_ − T_nπ*_ conversion pathways. Accordingly, the influence of substituents on photoluminescence quantum yield (PLQY) in different heteroaromatic series should be regarded as inherently structure-dependent rather than governed by a universal substituent effect.

**Figure 3:**
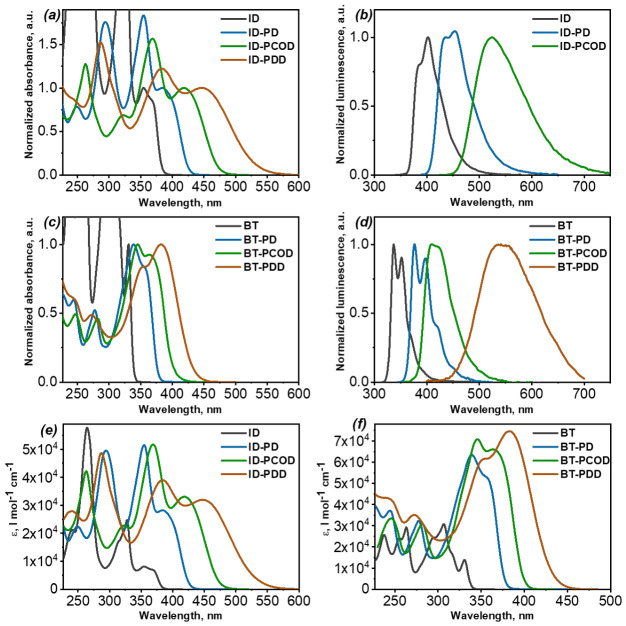
UV–Vis normalized absorption (**a**,**c**) and luminescence (**b**,**d**) spectra of the model compounds and target molecules in THF solution. Spectral distribution of the extinction coefficient of compounds in THF solution: ID-based derivatives (**e**) and BT-based derivatives (**f**).

The optical properties of the model and target molecules were investigated using UV–Vis absorption and photoluminescence spectroscopy in diluted THF solutions (Figure 3) as well as in a solid state (Figure S31). The corresponding data are summarized in Table 1.

#### 2.2.1. Photophysical Properties in THF Solution

As illustrated in Figure 3a, the absorption spectrum of the model ID comprises multiple transitions, with the longest wavelength bands exhibiting a maximum at 354 nm and a shoulder at 370 nm. The intensity difference between the long and short wavelength absorption bands is 7 times. The sequential introduction of substituents phenyldecyl (PD), phenylcarbonyl decyl (PCOD) and phenyl dicyanovinyl decyl (PDD) results in the displacement of the longest wavelength π→π* transition to the lower energy region, while the value of the extinction coefficient at the maximum of this band increases 4–5 times (Figure 3e).

The fluorescence spectrum of the model ID has a vibrational structure (Figure 3b), and the PLQY is 58%. The introduction of substituents shifts the fluorescence spectrum to the long-wavelength region. The compound ID-PD exhibits a slightly modified vibrational structure, resulting in an increased PLQY of 76%. In the compound ID-PCOD, the fluorescence spectrum is broad and structureless, and the PLQY is 92%. Probably, the increase in PLQY is due to the fact that the energy of π→π* transitions decreased with increasing π-conjugation, while n→π* transitions increased [33]. Consequently, the probability of S_ππ*_ − T_nπ*_ conversion decreased. The replacement of the carbonyl group with a strong dicyanovinyl group leads to the disappearance of fluorescence in the ID-PDD compound. As a result of this substitution, the number of heteroatoms in the system and, by extension, the number of nπ*-states whose energy is proximate to that of the S_1_→S_0_ state increased. Consequently, the probability of S_ππ*_ − T_nπ*_ conversion increased. The sequential introduction of PD and PCOD substituents to the ID core leads to a shift in the 0–0 transition to the low energy region at 2600 and 5200 cm^−1^, respectively, relative to the unsubstituted core, and an increase in the Stokes shift from 1100 to 3100 and 4800 cm^−1^.

As illustrated in Figure 3c the absorption spectrum of compound BT comprises multiple transitions. The longest wavelength transition, which is detached, is situated at 331 nm. The intensity of the most prominent transition was found to be 2.2 times higher than that of the longest wavelength transition (Figure 3f). As observed in the case of the ID core, the sequential introduction of PD, PCOD, and PDD substituents results in the shift in the longest wavelength transition to the lower energy region, thereby increasing the extinction coefficient at the maximum of this band by a factor of 4–5 (Figure 3f).

The fluorescence spectrum of the BT model compound is structural (Figure 3d) and the PLQY is 3%. Introducing substituents into the BT structure shifts the fluorescence spectrum towards the long-wavelength region. In BT-PD, the shape of the fluorescence spectrum is very close to the BT, from which it can be concluded that their vibrational structures are identical. Furthermore, the PLQY increases by up to 38%. When the carbonyl substituent (compound BT-PCOD) is added, the structure of the fluorescence spectrum disappears and the PLQY decreases to 19%. The fluorescence spectrum of compound BT-PDD is structureless and the PLQY is 10%. In general, ID-based compounds (with the exception of ID-PDD) exhibit a higher PLQY in THF solution than BT-based compounds. The integral extinction coefficient for both groups of compounds is similar.

#### 2.2.2. Quantum-Chemical Calculations

**Figure 4:**
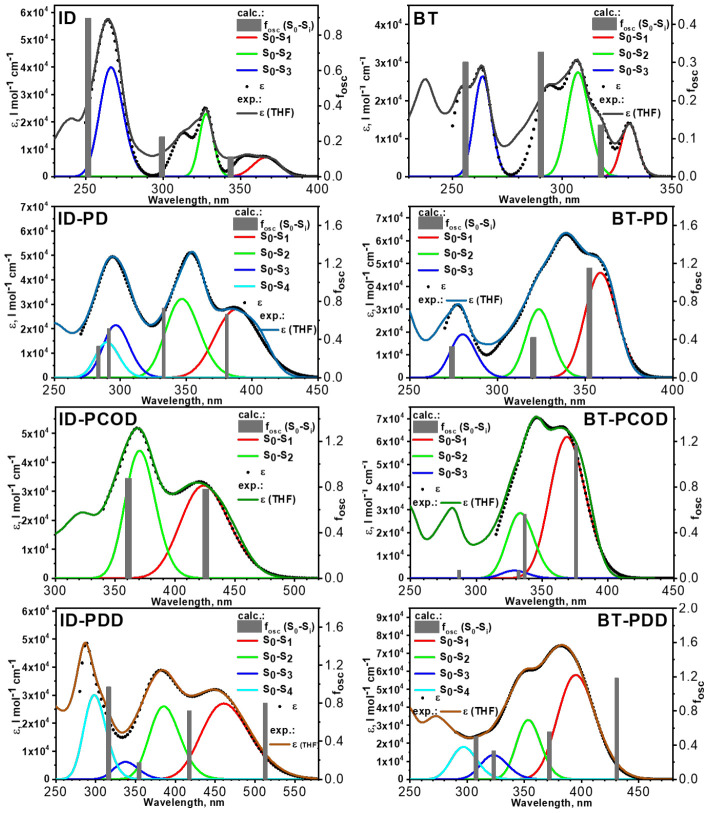
Calculated and experimental extinction coefficient spectra for condensed compounds, based on ID and BT cores.

The results of the calculation of purely electronic transitions, compared with experimental absorption spectra in THF solution, are presented in Table 2 and Figure 4 (the most significant transitions, with f_osc_ > 0.05, are indicated). The orbitals corresponding to the HOMO and LUMO states of the studied compounds are shown in Figure S29.

According to the calculation results, introducing PD, PCOD and PDD substituents into unsubstituted cores consistently shifts the position of the long-wavelength transition to a lower-energy region, as observed experimentally. For the ID and BT cores and the ID-PD and BT-PD compounds, the experimental transition position is in the lower energy region compared to the calculated position (1230–1800 cm^−1^ for the ID and BT cores and 500 cm^−1^ for the compounds with the substituent PD). For the ID-PCOD compound, the experimental transition position is close to the calculated position. For the BT-PCOD compounds (500 cm^−1^), and especially for the ID-PDD and BT-PDD compounds (2100–2200 cm^−1^), the experimental transition position is in the higher energy region compared to the calculated position. In the latter case, this may be due to the introduction of strong EWGs that significantly alter the molecule’s dipole moment and determine its interaction with the solvent.

The oscillator strength of the long-wavelength transition changes most sharply when the PD substituent is introduced into the unsubstituted core (increasing from 0.1 to 0.7). Subsequent introduction of the PCOD and PDD substituents has little effect on this value. This is consistent with the observed behavior of the spectral distribution of the extinction coefficient (Figure 3e,f). During the transition to the excited state in compounds containing substituents, electron density increases on the substituents and decreases on the central nucleus (Figure S29).

In the formation of the absorption spectra of these condensed compounds, apart from purely electronic transitions, vibrational components have a significant contribution, which could not be obtained in the calculation. By focusing on the number of electronic transitions and oscillator strength obtained from the calculation, it was possible to decompose the absorption spectra of condensed compounds into electronic and vibrational components. To compare the calculated and experimental data, we applied the approach proposed in the work [35] and realized by us earlier for various objects [36,37]. The model absorption spectra were constructed by summing individual bands having the form of Gaussian functions for electronic and vibrational transitions. The oscillator strength of each band was calculated by the following formula:

(1) $$f_{osc}={\left(n_{D}\right)}^{2}\cdot 4.32\cdot 10^{-9}\int_{\tilde{\nu }}\varepsilon_{\tilde{\nu }}d\tilde{\nu },$$

where $n_{D}$ is the refractive index of the solvent (THF), $\varepsilon_{\tilde{\nu }}$ is the molar extinction coefficient taken in the wave number scale $\tilde{\nu }$, cm^−1^.

The compounds under consideration are characterized by well-defined mirror symmetry in their absorption and fluorescence spectra. The electronic and vibrational components of the long-wavelength transition are located in the region of the reflected fluorescence spectrum (Figure S30).

In general, the calculated and experimental absorption spectra show a satisfactory correlation (Figure 4). The largest deviation of the long-wavelength transition oscillator strength from the calculated value is observed for BT and BT-PD, at up to 40%. For the other compounds, this deviation does not exceed 10%. The value of the vibrational component obtained from the decomposition of the experimental absorption spectra for specific transitions is comparable to, if not greater than, the electronic component (Table 2).

As the calculations show, the position of the fluorescence transition S_1_→S_0_ shifts to the long-wavelength region of the spectrum when substituents are introduced to the cores sequentially. This is consistent with the experimental data. The fluorescence oscillator strength increases by around a factor of six when the PD substituent is attached to an unsubstituted core. Additionally, the fluorescence oscillator strength is notably higher in BT-PDD than in ID-PDD (1.53 versus 0.38). Table 2 shows the calculated fluorescence constant (k_r_) and singlet-triplet conversion constant (k_nr_) values obtained based on the calculated fluorescence transition oscillator strength and measured quantum yield of the studied compounds. The calculated singlet–triplet conversion constant values confirm the qualitative explanation of the quantum yield dependence on substituent type stated above.

In general, the oscillator strength and energy of the oscillator transitions of the studied compounds, as obtained from TDDFT, are in good agreement with experimental results.

#### 2.2.3. Photophysical Properties in Solid State

The absorption spectra of the films and the fluorescence spectra of the powders are presented in Figure S31; a common feature of the absorption spectra of films of all compounds is their bathochromic shift compared to solutions, while retaining approximately the same set of absorption bands. The absorption spectra of films with the PDD substituent most closely resemble those of solutions, while the absorption bands of the other films are characterized by redistribution of intensity. This may indicate a change in the conformation of the molecules in the films compared to the molecules in solution.

All maxima of the fluorescence spectra of powders (except BT-PDD) are shifted to the long-wave region of the spectrum compared to the solution ones. It was also generally observed that the PLQY of the powders decreased compared to the THF solution, except for the BT-PDD compound, for which it increased twofold.

The model ID and ID-PD retained a weakly expressed structural form of the fluorescence spectrum. The model compound BT, along with compounds BT-PD and BT-PCOD in powder form, demonstrate a clearly defined structural shape in their fluorescence spectra. In general, ID-based compounds (with the exception of ID-PDD) exhibit higher PLQY in the solid state than BT-based compounds.

### 2.3. Electrochemical Properties

**Figure 5:**
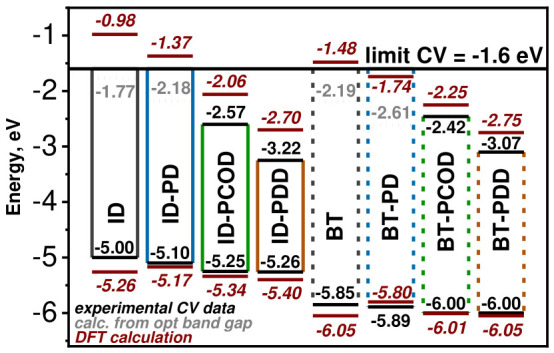
Experimental (black) and calculated (grey and red) energy levels for model and target compounds in THF solution.

The electrochemical behavior of the model and target compounds was investigated with cyclic voltammetry (CV) technique. Using oxidation and reduction potentials, experimental energies of the highest occupied molecular orbital (HOMO) and the lowest unoccupied molecular orbital (LUMO) levels and the corresponding electrochemical bandgap values were calculated. DFT calculation of the HOMO and LUMO energies was also performed (Figure 5; Table S1, Figure S32).

It can be concluded that both carbonyl and dicyanovinyl EWGs lower the electron density on ID and BT cores and significantly hinder oxidation (∆E = 220 mV for ID and ∆E = 200 mV for BT) compared to model ID and BT. The utilization of a more electron-saturated ID core results in the oxidation of ID series molecules being more facile than that of BT series molecules. The oxidation potentials for all pairs of ID series and BT series differ by approximately 0.7–0.8 eV.

The values of the reduction potentials of ID-PCOD and BT-PCOD are significantly shifted to the cathodic region (∆E = 0.65 V) compared to the reduction potentials of ID-PDD, BT-PDD, seen from the comparison of cathodic potentials. The hindered reduction in both cases is due to the fact that the carbonyl group with the donor alkyl substituent is more electron-saturated than the dicyanovinyl group. The experimental values correspond well with the calculated ones. This confirms that the ID core is a stronger electron donor than the BT core. Consequently, the entire ID series exhibits higher HOMO energy levels and a lower electrochemical bandgap than the BT series, which is consistent with the optical absorption data.

### 2.4. Solubility and Thermal Properties

The solubility of all synthesized materials was evaluated in tetrahydrofuran (THF) at room temperature (Table 3). Derivatives bearing dicyanovinyl groups exhibited the highest solubility. Specifically, the solubility of ID-PDD and BT-PDD increased by factors of 2 and 10, respectively, relative to derivatives lacking electron-withdrawing groups (EWGs). This behavior may be attributed to the relatively large size of the dicyanovinyl group, which substantially influences molecular packing and intermolecular interactions. In contrast, the introduction of carbonyl EWGs into ID-PD and BT-PD resulted in a decrease in solubility. The ID derivatives exhibited higher solubility than their BT analogues. Notably, BT-PDD represents a clear exception to this trend, a finding that is indirectly supported by its lower isotropization temperature and enthalpy (see Section 2.5).

The thermal stability of all synthesized molecules was investigated using thermogravimetric analysis (TGA). The data is presented in Table 3 and Figure S33. All materials showed relatively high thermal destruction temperatures (*T*_d_—calculated as 5% loss of sample weight) in air (above 300 °C, with the exception of BT-PD) and in inert atmospheres (above 350 °C). The introduction of EWGs (PCOD and PDD) led to an enhancement in the thermal stability of derivatives of both donor cores. In general, the BT derivatives exhibit greater thermal stability. However, ID derivatives exhibit enhanced resistance to thermal oxidation, a property that is particularly evident in the pair with PD substituents. For instance, the *T*_d_ of ID-PD is 84 °C higher than that of BT-PD. It is noteworthy that for molecules with EWGs, incomplete destruction was observed in an inert atmosphere (Figure S33b). The phenomenon may be associated with the generation of thermostable compounds within the specified temperature range, as well as/or with incomplete oxidation [31].

### 2.5. X-Ray Diffraction and Phase Behavior

**Figure 6:**
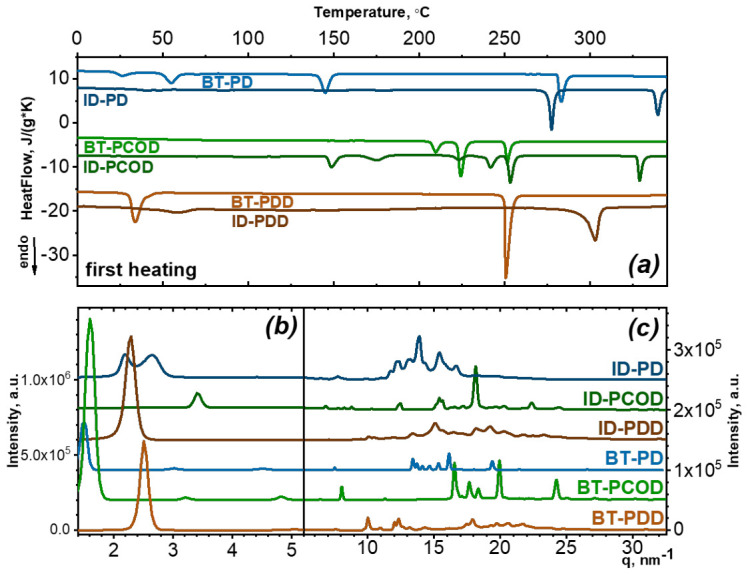
DSC first heating scans for the obtained compounds (**a**); powder XRD curves obtained at room temperature, shifted for clarity (**b**). The SAXS region of the curves (**c**).

All samples tend to form highly crystalline powders at room temperature (Figure 6, Table 4). However, significant changes in the SWAXS (small- and wide-angle X-ray scattering) curves occur when the chemical structure is modified, demonstrating sets of sharp reflections. All patterns were indexed and consequently refined to obtain the crystal parameters. The characteristic feature is the presence of a strong small-angle peak indicating a relatively large crystal cell parameter aligned roughly along the molecular axis. Different types of crystal systems are observed, as can be seen in Table 4. The number of molecules per crystal cell at room temperature also varies from 2 to 4, but the estimated density does not change significantly. It is important to note that the cross section is close to ~100 A^2^ for all compounds studied, indicating a similar type of ordering of the conjugated fragments.

To further analyze phase behavior in situ heating was conducted with constant rate of 7°/min (Figures S36–S41), and the experimental upper limit was 250 °C. While some samples show relatively simple phase sequence (ID-PDD and BT-PDD), others demonstrate a rich variety of crystal–crystal transitions, also showing a wide range of phase coexisting. The phase behavior of compounds was investigated using differential scanning calorimetry (DSC). The data is presented in Figure 6a and Figure S34 and Table S2. The DSC curves show mostly enantiotropic transitions, which are repeated during the second heating.

Both ID-PDD and BT-PDD exhibit two phase transitions on their DSC curves (Figure 6a). For ID-PDD, similar to ID-PD, the transition happens already at 60 °C; however, further heating leads to intensity growth due to structure annealing. The new phase was indexed as triclinic, while the as-received crystal was hexagonal. The longest parameter obviously did not change, same as the crystal volume. This transition in ID-PDD is not enantiotropic and does not reproduce upon reheating (Figure S34), which may be advantageous for device fabrication. As demonstrated by XRD, BT-PDD shows one transition at higher temperatures, close to 220 °C. Indexing showed triclinic cell change to monoclinic with doubling of the crystal cell and slight shortening of the longest parameter, probably to inclination of the molecular axis. The high-temperature endothermic effects on the DSC for both ID-PDD and BT-PDD correspond to melting, as confirmed by polarized optical microscopy (POM) (Figure S35f,l). ID-PCOD also exhibited complex phase behavior, showing four distinct crystal phases at room temperature, 170 °C, 180 °C and 210 °C, while further heating showed signs of melting through a low-ordered smectic LC state (with a parameter of 36.2 Å). It is evident from the DSC ID-PCOD curve that six endothermic effects occur during the first heating. Comparison with XRD data indicates that the first three transitions (at 148 °C, 175 °C, and 223 °C) correspond to transitions between crystalline phases. These are followed by the formation of a smectic state beginning at approximately 230 °C, and subsequently by melting, as confirmed by POM (Figure S35e). The obtained crystal parameters varied widely, demonstrating different crystal systems and number of molecules per crystal cell. Heating of the BT-PCOD resulted in two crystal-crystal transitions, slightly decreasing the longest crystal parameter while maintaining the monoclinic symmetry; however, the latter transition yielded broad reflections due to small crystal size and its imperfection. On the DSC curve, BT-PCOD exhibits three endothermic effects, each corresponding to a transition between crystalline states (Figure S40, Table 4). POM analysis showed that melting of BT-PCOD was not observed up to the maximum temperatures within the investigated range (Figure S35k). ID-PD exhibits three endothermic transitions, each of which is enantiotropic and reproducible upon reheating. During POM analysis at 350 °C, the onset of melting is observed for ID-PD (Figure S35d), whereas melting is not detected for BT-PD within the entire studied temperature range (Figure S35j). Notably, BT-PD displays the most complex phase behavior below 150 °C; all endothermic effects observed in this region correspond to transitions between crystalline phases (Figure S38, Table 4). For BT-PD, three types of crystal patterns can be clearly identified: the as-received, heated up to 70 °C and the high temperature starting from ~150 °C. While all of these crystals appeared to be monoclinic with the longest axis of ~41Å, the cross section varied significantly, leading to substantial crystal volume change.

Overall, XRD data generally corroborate the DSC findings, with minor discrepancies attributable to heating rate effects and crystal anisotropy. While the compounds demonstrated a rich diversity of crystal packings upon slight changes in chemical structure, determining the precise molecular packing in such crystals is a sophisticated task and should be carried out in a separate study, likely with the aid of single-crystal data. A comparison of ID derivatives to BT derivatives shows similar layered crystalline structures with one anisotropic crystal axis substantially larger than the others. This specific morphological feature governs key material properties, including optical characteristics and charge transport.

## 3. Materials and Methods

### 3.1. Materials

Tetrakis(triphenylphosphine)palladium(0) Pd(PPh_3_)_4_, [1,1′-bis(diphenylphosphino)ferrocene]dichloropalladium(II) Pd(dppf)Cl_2_, malononitrile, bromobenzene, 1,4-dibromobenzene, decyl bromide, magnesium, undecanoyl chloride, aluminum chloride, 2,2-dimethyl-1,3-propanediol, p-toluenesulfonic acid (p-TosH), n-butyl lithium (2.5 M solution in hexane), and 2-isopropoxy-4,4,5,5-tetramethyl-1,3,2-dioxaborolane (IPTMDOB) were obtained from Sigma–Aldrich Co (St Louis, MO, USA) and used without further purification. THF, toluene, ethanol, petroleum ether, pyridine, chloroform, and benzene were dried and purified according to the known techniques and then used as solvents. All reactions, unless stated otherwise, were carried out under an inert atmosphere. Knövenagel condensation was carried out in the microwave (Discovery, CEM corporation, Matthews, NC, USA), using a standard method with the open vessel option, 50 watts.

### 3.2. Synthetic Procedures

Below, details of the synthesis of the compounds from Figure 2 are described.

2,7-dibromo-5,10-dimethyl-5,10-dihydroindolo[3,2-b]indole (**1**). A round bottom flask equipped with a magnetic stirrer and a reflux condenser was baked, cooled and filled with Ar. After, 2,7-dibromo-5,10-dihydroindolo[3,2-b]indole (4.0 g, 11.0 mmol), NaH (1.75 g, 44.0 mmol) and anhydrous THF (105 mL) were added to the reaction vessel. Then 6.2 g (44.0 mmol) of iodomethane was added to the reaction mixture at room temperature. The reaction mixture was then heated for 3 h at 40 °C. Then aqueous hydrochloric acid solution and diethyl ether were added to the reaction mixture for neutralization at room temperature. The obtained mixture was passed through a glass filter. The residue was purified by washing with water, diethyl ether and methanol. The product **1** is obtained as a gray powder (3.79 g, 88% yield). ^1^H NMR (300 MHz, CDCl_3_): δ [ppm] 4.05 (s, 6H), 7.28 (dd, 2H, J_1_ = 1.2 Hz, J_2_ = 8.4 Hz), 7.56 (d, 2H, J = 1.2 Hz), 7.71 (d, 2H, J = 8.5 Hz). Anal. calcd. (%) for C_16_H_12_Br_2_N_2_: C, 49.01; H, 3.08; N, 7.14. Found: C, 48,95; H, 3.13; N, 7.12. MALDI-TOF MS: found *m*/*z* 391.93; calculated for [M]^+^ 391.93.

5,10-dimethyl-5,10-dihydroindolo[3,2-b]indole (**ID**). A 2.5 M solution of n-butyllithium (0.38 mL, 0.9 mmol) in hexane was added drop wise to a solution of compound **1** (155 mg, 0.4 mmol) in 100 mL of dry THF at −80 °C. The reaction mixture was then stirred for 1 h at −80 °C, after which a 1 M aqueous hydrochloric acid solution (0.95 mL, 0.9 mmol) was added in one portion at −80 °C. Then to the reaction mixture was extracted in a mixture of diethyl ether–water. The organic phase was combined, the solvent was evaporated in vacuum and the residue was dried at 1 Torr. The product was purified by column chromatography on silica gel (eluent: toluene) to give pure compound ID (67 mg, 73%) as a white powder. ^1^H NMR (250 MHz, CDCl_3_): δ [ppm] 4.11 (s, 6H), 7.13–7.20 (overlapping peaks, 2H), 7.26–7.35 (overlapping peaks, 2H), 7.44 (d, 2H, J = 8.2 Hz), 7.90 (d, 2H, J = 7.9 Hz). Anal. calcd (%) for C_16_H_14_N_2_: C, 82.02; H, 6.02; N, 11.96. Found: C, 82.13; H, 5.97; N, 12.01. MALDI-TOF MS: found *m*/*z* 234.20; calculated for [M]^+^ 234.12.

1-bromo-4-decylbenzene (**3**). Decylmagnesium bromide, obtained from decyl bromide (7.5 g, 33.9 mmol) and magnesium (0.98 g, 40.7 mmol) in diethyl ether (25 mL), was added dropwise to a mixture of 1,4-dibromobenzene (10.0 g, 42.2 mmol) and PdCl_2_(dppf) (77.5 mg, 0.1 mmol) in diethyl ether (50 mL) at 0°C. After stirring at room temperature for 2 days, the reaction mixture was extracted in water–diethyl ether system. The combined organic phases were dried over sodium sulfate and filtered. The solvent was evaporated in vacuum and the residue was dried at 1 Torr. Precursor **3** was purified by vacuum distillation and obtained as a colorless liquid (10.08 g, 80%). ^1^H NMR (300 MHz, CDCl_3_): δ [ppm] 0.88 (t, 3H, J = 6.8 Hz), 1.24–1.31 (overlapping peaks, 14H), 1.52–1.62 (m, 2H), 2.54 (t, 2H, J = 7.9 Hz), 7.04 (d, 2H, J = 8.5 Hz), 7.38 (d, 2H, J = 8.5 Hz). 13C NMR (75 MHz, CDCl_3_): δ [ppm] 14.12, 22.68, 29.17, 29.31, 29.45, 29.56, 29.59, 31.33, 31.89, 35.34, 119.20, 130.15, 131.21, 141.83. Anal. calcd (%) for C_16_H_25_Br: C, 64.64; H, 8.48. Found: C, 64.52; H, 8.39.

2-(4-Decylphenyl)-4,4,5,5-tetramethyl-1,3,2-dioxaborolane (**4**). A 2.5 M solution of n-butyllithium (6.36 mL, 15.9 mmol) in hexane was added drop wise to a solution of compound **3** (5.0 g, 15.1 mmol) in 180 mL of dry THF at −30 °C. Afterwards the reaction mixture was stirred for 1 h at −30 °C and then IPTMDOB (2.9 g, 15.9 mmol) was added in one portion at −30 °C. The reaction mixture was stirred for 1 h at −30 °C, then the cooling bath was removed, and the stirring was continued for 1 h. After completion of the reaction, freshly distilled diethyl ether, distilled water and aqueous 1 M HCl solution were added to the reaction mixture. The organic phase was separated, washed with water, and dried over sodium sulfate and filtered. The solvent was evaporated to give 5.04 g (96%) of compound **4** as a colorless liquid, which was used in next stage without any purification. ^1^H NMR (300 MHz, CDCl_3_): δ [ppm] 0.88 (t, 3H, J = 6.8 Hz), 1.24–1.29 (overlapping peaks, 14H), 1.33 (s, 12H), 1.56–1.63 (m, 2H), 2.61 (t, 2H, J = 7.8 Hz), 7.19 (d, 2H, J = 7.9 Hz), 7.73 (d, 2H, J = 7.9 Hz). ^13^C NMR (75 MHz, CDCl_3_): δ [ppm] 14.11, 22.67, 24.83, 29.31, 29.48, 29.56, 29.59, 29.69, 31.34, 31.89, 36.17, 83.57, 127.89, 128.18, 134.78, 146.43. Anal. calcd (%) for C_22_H_37_BO_2_: C, 76.74; H, 10.83. Found: C, 76.81; H, 10.89.

1-(4-bromophenyl)-1-undecanone (**5**). Undecanoyl chloride (16.7 g, 81.8 mmol) was added dropwise to a stirred suspension of aluminium trichloride (AlCl_3_) (13.1 g, 98.2 mmol) in bromobenzene (25.7 g, 163.7 mmol). After complete addition, the suspension was stirred for 1 h at 50 °C. The reaction mixture (with temperature 50 °C) was carefully poured into ice water and extracted three times with dichloromethane. The organic phase was combined, dried over MgSO_4_, the solvent was evaporated in vacuum and the residue was dried at 1 Torr. The product was purified by column chromatography on silica gel (eluent—toluene: petroleum ether, 1:2) to give pure compound **5** (18.6 mg, 70%) as a white powder. ^1^H NMR (300 MHz, CDCl_3_): δ [ppm] 0.86 (t, 3H, J = 6.8 Hz), 1.23–1.38 (overlapping peaks, 14H), 1.65–1.75 (m, 2H), 2.91 (t, 2H, J = 7.6 Hz), 7.58 (d, 2H, J = 8.5 Hz), 7.80 (d, 2H, J = 8.7 Hz). ^13^C NMR (75 MHz, CDCl_3_): δ [ppm] 14.09, 22.66, 24.24, 29.29, 29.43, 29.47, 29.54, 31.86, 38.57, 127.95, 129.58, 131.82, 135.74, 199.48. Anal. calcd (%) for C_17_H_25_BrO: C, 62.77; H, 7.75. Found: C, 62.82; H, 7.79.

2-(4-bromophenyl) -5,5-dimethyl-2-decyl -1,3-dioxane (**6**). Compound **5** (16.4 g, 50.5 mmol) was dissolved in dry benzene (164 mL). After complete dissolution 2,2-dimethyl-1,3 propanediol (26.3 g, 252.7 mmol) and *p*-TosH (1.9 g, 10.1 mmol) were added. Then the mixture was stirred at reflux for 22 h using a Dean–Stark water separator. Then, after cooling the reaction mixture to room temperature, triethylamine was added and the mixture was extracted in a water–toluene system. The combined organic phases were dried over sodium sulfate and filtered. The solvent was evaporated in vacuum and the residue was dried at 1 Torr. This crude product was purified by column chromatography on silica gel (eluent—toluene: petroleum ether, 1:4) to give pure compound **6** (18.3 g, 87%) as a colorless liquid. ^1^H NMR (300 MHz, CDCl_3_): δ [ppm] 0.56 (s, 3H), 0.85 (t, 3H, J = 7.0 Hz), 1.17–1.33 (overlapping peaks, 19H), 1.64–1.72 (overlapping peaks, 2H), 3.36 (s, 4H), 7.24 (d, 2H, J = 8.7 Hz), 7.49 (d, 2H, J = 8.5 Hz). ^13^C NMR (75 MHz, CDCl_3_): δ [ppm] 14.11, 21.86, 22.66, 22.93, 29.27, 29.54, 29.56, 29.59, 29.70, 30.04, 31.88, 44.55, 71.54, 101.36, 121.63, 129.27, 131.57, 139.17. Anal. calcd (%) for C_22_H_35_BrO_2_: C, 64.23; H, 8.58. Found: C, 64.19; H, 8.55.

2-decyl-5,5-dimethyl-2-[4-(4,4,5,5-tetramethyl-1,3,2-dioxaborolan-2-yl)phenyl]-1,3-dioxane (**7**). 2.5 M solution of n-butyllithium (18.4 mL, 46.1 mmol) in hexane was added drop wise to a solution of compound **6** (18.1 g, 43.9 mmol) in 360 mL of dry THF at −80 °C. Afterwards the reaction mixture was stirred for 1,5 h at −80 °C and then IPTMDOB (0.93 g, 5 mmol) was added in one portion at −80 °C. The reaction mixture was stirred for 1 h at −80 °C, then the cooling bath was removed, and the stirring was continued for 1 h. After completion of the reaction, freshly distilled diethyl ether, distilled water and aqueous 1 M HCl solution were added to the reaction mixture. The organic phase was separated, washed with water, and dried over sodium sulfate and filtered. The solvent was evaporated to give 18.9 g (94%) of compound **7** as a white solid, which was used in the next stage without any purification. ^1^H NMR (300 MHz, Acetone-d_6_): δ [ppm] 0.55 (s, 3H), 0.85 (t, 3H, J = 6.8 Hz), 1.18–1.29 (overlapping peaks, 19H), 1.33 (s, 12H), 1.62–1.68 (overlapping peaks, 2H), 3.37 (s, 4H), 7.37 (d, 2H, J = 8.3 Hz), 7.77 (d, 2H, J = 8.2 Hz). ^13^C NMR (75 MHz, Acetone-d_6_): δ [ppm] 14.32, 21.96, 23.19, 23.26, 23.61, 25.17, 29.97, 30.23, 30.25, 30.40, 30.47, 32.55, 45.32, 72.01, 84.47, 102.18, 127.46, 135.59, 144.44. Anal. calcd (%) for C_28_H_47_BO_4_: C, 73.35; H, 10.33. Found: C, 73.46; H, 10.41.

2,7-bis[4-(2-decyl-5,5-dimethyl-1,3-dioxan-2-yl)phenyl]-5,10-dimethyl-5,10-dihydroindolo[3,2-b]indole (**8**). To compound **1** (0.25 g, 0.6 mmol), compound 7 (0.70 g, 1.5 mmol), and Pd(PPh_3_)_4_ (37 mg, 0.03 mmol) in inert atmosphere were added degassed toluene (25 mL), ethanol (3.8 mL), and 2M K_2_CO_3_ aqueous solution (2.3 mL). The reaction mixture was stirred under reflux for 6 h. After cooling to room temperature, the reaction mixture was poured into a water–toluene mixture and extracted. The organic phase was separated, and solvent was evaporated under reduced pressure. The residue was purified by column chromatography on silica gel (eluent: toluene) to give pure product **8** (0.36 g, 63% yield) as a yellow-green solid. ^1^H NMR (300 MHz, CDCl_3_): δ [ppm] 0.87 (t, 6H, J = 6.9 Hz), 1.16–1.49 (overlapping peaks, 44H), 1.76–1.84 (m, 4H), 3.39–3.57 (m, 8H), 4.18 (s, 6H), 7.44–7.52 (overlapping peaks, 6H), 7.67 (d, 2H, J = 1.1 Hz), 7.74 (d, 4H, J = 8.3 Hz), 7.92–7.98 (m, 2H). Anal. calcd (%) for C_60_H_82_N_2_O_4_: C, 80.49; H, 9.23; N, 3.13. Found: C, 80.40; H, 9.21; N, 3.15. MALDI-TOF MS: found *m*/*z* 894.13; calculated for [M]^+^ 894.63.

2,2′-[benzothieno[3,2-b]benzothiene-2,7-diylbis(4,1-phenylene)]bis(2-decyl-5,5-dimethyl-1,3-dioxane) (**9**). To compound **2** (1.80 g, 4.5 mmol), compound 7 (6.22 g, 13.6 mmol), and Pd(PPh_3_)_4_ (470 mg, 0.4 mmol) in inert atmosphere were added degassed toluene (155 mL), ethanol (20 mL), and 2M Na_2_CO_3_ aqueous solution (23 mL). The reaction mixture was stirred under reflux for 17 h. After cooling to room temperature, the reaction mixture was poured into a water–toluene mixture and extracted. The organic phase was separated, and solvent was evaporated under reduced pressure. The residue was purified by column chromatography on silica gel (eluent: toluene) to give pure product **9** (2.08 g, 51% yield) as a white solid. ^1^H NMR (250 MHz, CDCl_3_): δ [ppm] 0.60 (s, 6H), 0.85 (t, 6H, J = 6.7 Hz), 1.16–1.27 (overlapping peaks, 28H), 1.29 (s, 6H), 1.35–1.48 (broadened signal, 4H), 1.72–1.85 (m, 4H), 3.37–3.57 (overlapping peaks, 8H), 7.50 (d, 4H, J = 8.2 Hz), 7.67–7.78 (overlapping peaks, 6H), 7.95 (d, 2H, J = 8.8 Hz), 8.17 (d, 2H, J = 1.2 Hz). Anal. calcd (%) for C_58_H_76_S_2_O_4_: C, 77.29; H, 8.50; S, 7.11. Found: C, 77.21; H, 8.48; S, 7.14.

2,7-bis(4-decylphenyl)-5,10-dimethyl-5,10-dihydroindolo[3,2-b]indole (**ID-PD**). To compound **1** (0.36 g, 0.9 mmol), compound 4 (0.79 g, 2.3 mmol), and Pd(PPh_3_)_4_ (66 mg, 0.1 mmol) in inert atmosphere were added degassed THF (25 mL), and 2M Na_2_CO_3_ aqueous solution (3.4 mL). The reaction mixture was stirred under reflux for 18 h. After cooling to room temperature, the reaction mixture was poured into water and diethyl ether. The organic phase was separated, and solvent was evaporated under reduced pressure. The residue was purified by column chromatography on silica gel (eluent: toluene) and recrystallization in toluene. Final product was further dissolved in THF and precipitated with petroleum ether to give 0.29 g (48% yield) of ID-PD as a gray green solid. ^1^H NMR (300 MHz, Acetone-d_6_): δ [ppm] 0.87 (t, 6H, J = 6.8 Hz), 1.25–1.36 (overlapping peaks, 28H), 1.52–1.60 (m, 4H), 2.13 (t, 4H, J = 7.5 Hz), 4.24 (s, 6H), 7.31 (d, 4H, J = 8.1 Hz), 7.46 (dd, 2H, J_1_ = 1.5 Hz, J_2_ = 8.3 Hz), 7.71 (d, 4H, J = 8.3 Hz), 7.80 (d, 2H, J = 1.5 Hz), 8.04 (d, 2H, J = 8.1 Hz). Anal. calcd (%) for C_48_H_62_N_2_: C, 86.43; H, 9.37; N, 4.20. Found: C, 86.39; H, 9.35; N, 4.26. MALDI-TOF MS: found *m*/*z* 666.54; calculated for [M]^+^ 666.49.

1,1′-[(5,10-dimethyl-5,10-dihydroindolo[3,2-b]indole-2,7-diyl)bis(4,1-phenylene)]diundecan-1-one (**ID-PCOD**). A total of 1 M HCl (0.8 mL) was added to a solution of compound **8** (0.35 g, 0.4 mmol) in THF (9 mL) and then the reaction mixture was stirred for 2 h at reflux at boiling temperature. After completion of the reaction the mixture was cooled to room temperature, and the organic phase was separated using diethyl ether, washed with water and filtered. The residue was purified by column chromatography on silica gel (eluent: chloroform). The final product was further dissolved in toluene and precipitated with petroleum ether to give 0.26 g (94% yield) of ID-PCOD as a yellow-orange solid. ^1^H NMR (300 MHz, CDCl_3_): δ [ppm] 0.89 (t, 6H, J = 7.0 Hz), 1.25–1.45 (overlapping peaks, 28H), 1.74–1.84 (m, 4H), 2.99 (t, 4H, J = 7.3 Hz), 4.18 (s, 6H), 7.48 (dd, 2H, J_1_ = 1.5 Hz, J_2_ = 8.3 Hz), 7.67 (d, 2H, J = 1.3 Hz), 7.81 (d, 4H, J = 8.4 Hz), 7.96 (d, 2H, J = 8.3 Hz), 8.05 (d, 4H, J = 8.4 Hz). ^13^C NMR (75 MHz, CDCl_3_): δ [ppm] 14.01, 22.66, 24.69, 29.31, 29.50, 29.52, 29.54, 29.60, 31.74, 31.91, 38.72, 108.39, 114.64, 117.92, 118.17, 127.32, 127.55, 128.73, 134.13, 135.54, 142.20, 146.75, 200.10. Anal. calcd (%) for C_50_H_62_N_2_O_2_: C, 83.06; H, 8.64; N, 3.87. Found: C, 82.98; H, 8.61; N, 3.91. MALDI-TOF MS: found *m*/*z* 721.96; calculated for [M]^+^ 722.48.

1,1′-([1]benzothieno[3,2-b][1]benzothiene-2,7-diylbis(4,1-phenylene))bis(undecan-1-one) (**BT-PCOD**). A total of 1 M HCl (4.4 mL) was added to a solution of compound **9** (1.98 g, 2.2 mmol) in THF (79 mL) and then the reaction mixture was stirred for 5 h at reflux at boiling temperature. After completion of the reaction the mixture was cooled to room temperature, and the organic phase was separated using diethyl ether, washed with water and filtered. The residue was purified by recrystallization in THF to give pure BT-PCOD (1.33 g, 83% yield) as a light-yellow solid. ^1^H NMR (300 MHz, CDCl_3_): δ [ppm] 0.89 (t, 6H, J = 6.5 Hz), 1.27–1.30 (overlapping peaks, 28H), 1.75–1.83 (m, 4H), 3.00 (t, 4H, J = 7.6 Hz), 7.72–7.82 (overlapping peaks, 6H), 7.98 (d, 2H, J = 8.5 Hz), 8.06 (d, 4H, J = 8.1 Hz), 8.18 (d, 2H, J = 0.8 Hz). Anal. calcd (%) for C_48_H_56_S_2_O_2_: C, 79.07; H, 7.74; S, 8.79. Found: C, 78.98; H, 7.70; S, 8.84. MALDI-TOF MS: found *m*/*z* 729.77; calculated for [M + H]^+^ 729.38.

2,2′-[(5,10-dimethyl-5,10-dihydroindolo[3,2-b]indole-2,7-diyl)bis(4,1-phenyleneundec-1-yl-1-ylidene)]dimalononitrile (**ID-PDD**). Compound ID-PCOD (271 mg, 0.4 mmol), malononitrile (74 mg, 1.1 mmol) and dry pyridine (6.8 mL) were placed in a reaction vessel and stirred under argon atmosphere for 22 h at reflux using the microwave heating. After completeness of the reaction the mixture was cooled to room temperature, pyridine was evaporated in vacuum and the residue was dried at 1 Torr. The crude product was purified by column chromatography on silica gel (eluent: chloroform). The ID-PDD product was obtained as a bard solid (205 mg, 67%). ^1^H NMR (300 MHz, CDCl_3_): δ [ppm] 0.88 (t, 6H, J = 7.0 Hz), 1.25–1.41 (overlapping peaks, 28H), 1.48–1.59 (m, 4H), 3.01 (t, 4H, J = 7.7 Hz), 4.17 (s, 6H), 7.48 (dd, 2H, J_1_ = 1.5 Hz, J_2_ = 8.4 Hz), 7.62 (d, 4H, J = 8.4 Hz), 7.67 (d, 2H, J = 0.9 Hz), 7.86 (d, 4H, J = 8.4 Hz), 7.97 (d, 2H, J = 8.3 Hz). ^13^C NMR (75 MHz, CDCl_3_): δ [ppm] 13.98, 22.62, 28.67, 29.09, 29.23, 29.34, 29.45, 31.73, 31.85, 37.50, 83.93, 108.38, 112.88, 113.21, 114.75, 118.03, 118.08, 127.66, 127.81, 128.25, 133.08, 133.55, 142.22, 146.24, 179.62. Anal. calcd (%) for C_56_H_62_N_6_: C, 82.11; H, 7.63; N, 10.26. Found: C, 82.03; H, 7.70; N, 10.18. MALDI-TOF MS: found *m*/*z* 817.94; calculated for [M]^+^ 818.50.

2,2′-([1]benzothieno[3,2-b][1]benzothiene-2,7-diylbis(4,1-phenyleneundec-1-yl-1-ylidene))dimalononitrile (**BT-PDD**). Compound BT-PCOD (1.07 g, 1.5 mmol), malononitrile (290 mg, 4.4 mmol) and dry pyridine (27 mL) were placed in a reaction vessel and stirred under argon atmosphere for 25 h at reflux using the microwave heating. After completeness of the reaction the mixture was cooled to room temperature, pyridine was evaporated in vacuum and the residue was dried at 1 Torr. The crude product was purified first by column chromatography on silica gel (eluent:toluene) and then by recrystallization in a toluene–petroleum ether system. The BT-PDD product was obtained as a yellow solid (392 mg, 32%). ^1^H NMR (250 MHz, CDCl_3_): δ [ppm] 0.86 (t, 6H, J = 7.0 Hz), 1.21–1.40 (overlapping peaks, 28H), 1.43–1.53 (m, 4H), 3.01 (t, 4H, J = 7.3 Hz), 7.61 (d, 4H, J = 8.5 Hz), 7.73 (dd, 2H, J_1_ = 1.5 Hz, J_2_ = 8.5 Hz), 7.82 (d, 4H, J = 8.5 Hz), 7.99 (d, 2H, J = 8.2 Hz), 8.18 (d, 2H, J = 0.9 Hz). Anal. calcd (%) for C_54_H_56_N_4_S_2_: C, 78.60; H, 6.84; N, 6.79; S, 7.77. Found: C, 78.48; H, 6.94; N, 6.71; S, 7.82. MALDI-TOF MS: found *m*/*z* 824.68; calculated for [M]^+^ 824.39.

### 3.3. Methods

#### 3.3.1. NMR Spectra

^1^H and ^13^C NMR spectra were recorded using a “Bruker Avance II 300” spectrometer (Bruker Corporation, Karlsruhe, Germany) at 300.17 MHz and 75.48 MHz, respectively, and utilizing CDCl_3_ signal (7.25 ppm and 77.00 ppm, for ^1^H and ^13^C NMR, respectively) as the internal standard. In the case of ^1^H NMR spectroscopy, the compounds to be analyzed were taken in the form of 1% solutions in CDCl_3_ or Acetone-d6. In the case of ^13^C NMR spectroscopy, the compounds to be analyzed were taken in the form of 5% solutions in CDCl_3_. The spectra were then processed using the ACD Labs software (version 2016free). NMR spectra were recorded using the equipment of Collaborative Access Center “Center for Polymer Research” of ISPM RAS. Owing to the low solubility of ID-PD, BT-PD, BT-PCOD, and BT-PDD, their ^13^C NMR spectra could not be acquired because samples of sufficient concentration for the analysis could not be prepared.

#### 3.3.2. Elemental Analysis

Elemental analysis for carbon, nitrogen, and hydrogen was performed using a CHN automatic analyzer, CE 1106 (Italy). The sulfur content was determined by precipitation titration with BaCl_2_. Prior to the measurements, the samples were carefully prepared and dried to ensure reliable analytical results. All analyses were carried out under standard laboratory conditions, and the obtained values showed good reproducibility. The experimental error associated with the elemental analysis was estimated to be within the range of 0.30–0.50%.

#### 3.3.3. Mass Spectra

MALDI-TOF mass spectra were recorded using an Autoflex II Bruker (Bruker Daltonik GmbH, Karlsruhe, Germany) instrument with a resolution of FWHM 18000 (Bruker Corporation, Karlsruhe, Germany). The spectrometer was equipped with a nitrogen laser operating at a wavelength of 337 nm and a time-of-flight mass analyzer functioning in reflection mode. Measurements were performed at an accelerating voltage of 20 kV. Prior to the analysis, the samples were deposited onto a polished stainless-steel target plate and allowed to dry under ambient conditions. The spectra were acquired in the positive ion detection mode. To improve the signal quality and reproducibility, each final spectrum represented the accumulation of 300 individual spectra collected from different areas of the sample surface. 2,5-Dihydroxybenzoic acid (DHB, Acros, 99%) and α-cyano-4-hydroxycinnamic acid (HCCA, Acros, 99%) were used as matrix substances for sample preparation.

#### 3.3.4. Thermogravimetric Analysis

TGA was conducted in dynamic mode within the temperature range of 30 to 700 °C using a “Mettler Toledo TG50” (Greifensee, Switzerland) system equipped with an M3 microbalance. This system enabled the measurement of sample weights within a range of 0–150 mg with a precision of 1 μg. The heating and cooling rates were set at a constant rate of 10 °C/min. Each compound was studied in both air and an N_2_ flow of 200 mL/min.

#### 3.3.5. Differential Scanning Calorimetry

DSC scans were obtained using a “Mettler Toledo DSC30” (Greifensee, Switzerland) system, with a heating/cooling rate of 10 °C/min within a temperature range of 0–350 °C for all compounds. The N_2_ flow rate was set at 50 mL/min.

#### 3.3.6. Polarized Optical Microscopy

Polarized optical images were obtained by employing a polarizing microscope (Axioskop 40 A Pol, Zeiss AG, Jena, Germany) with a Lincam camera (Carl Zeiss, Jena, Germany) and a heating stage (THMS600, Linkam Scientific Instruments, Salfords, UK).

#### 3.3.7. UV–Vis Spectroscopy

The absorption spectra of molecular solutions in THF and of polycrystalline layers were measured on a UV-2501PC spectrophotometer (Shimadzu, Kyoto, Japan). Optical absorption spectra were recorded at room temperature in diluted THF solutions (10^−5^ M), polycrystalline layers cast from compounds solutions on quartz or glass substrates.

The absorption spectra of the compounds in solid state were measured for thin polycrystalline films.

#### 3.3.8. Fluorescence Spectroscopy

Fluorescence spectra were recorded using a FLUORAN-2 spectrophotometer–spectrofluorimeter (VNIIOFI, Moscow, Russia). For measurements of solution samples, standard quartz cuvettes with dimensions of 10 × 10 mm were employed. The fluorescence quantum yields of the solutions were determined by comparison with reference standards possessing known quantum yields, using the method based on measurements of optically diluted solutions [38]. Solutions of Rhodamine 6G in ethanol (ΦF = 0.95) and POPOP in cyclohexane (PLQY = 0.93) were used as standards for the determination of fluorescence quantum yields of the investigated luminophores [39]. The fluorescence spectra and quantum yields of powdered samples were measured using an integrating sphere made of highly reflective polytetrafluoroethylene. Prior to the experiments, the integrating sphere was calibrated using crystalline powder of Tetraphenyl butadiene, which is characterized by a high fluorescence quantum yield (PLQY = 0.95) and low reabsorption losses (k_reabs_ = 0.88). Such calibration ensured reliable measurements of solid-state fluorescence characteristics.

The fluorescence properties and quantum yield of the investigated compounds were studied in the powdered state. The corresponding fluorescence spectra are presented in Figure S31b,d. Excitation was carried out at the long-wavelength absorption maximum of the films. It should be noted that the fluorescence spectra of the powdered samples were significantly affected by reabsorption effects, leading to distortion of the spectral shape. Therefore, the reported fluorescence maxima and fluorescence quantum yield values should be considered as reference (“technical”) parameters that strongly depend on the specific measurement conditions employed. Consequently, a direct comparison between the fluorescence quantum yields of powders and those measured in solution can only be regarded as approximate and conditional.

#### 3.3.9. Chromatography

Gel permeation chromatography (GPC) analysis was performed on a “Shimadzu” instrument with a RID-10A refractometer and an SPD-M10AVP diode matrix as detectors (Kyoto, Japan) using 7.8 × 300 mm Phenomenex columns (Torrance, CA, USA) filled with the Phenogel sorbent with pore sizes of 500 and 103 Å and THF as the eluent. For thin layer chromatography, “Sorbfil” (Krasnodar, Russia) plates were used. In the case of column chromatography, silica gel 60 (Merck, Darmstadt, Germany) was taken.

#### 3.3.10. Cyclic Voltammetry

CV measurements were carried out using solid compact layers of the compounds, which in turn were made by electrostatically rubbing the materials onto a glassy carbon electrode using IPC-Pro M potentiostat (Saint Petersburg, Russian Federation). Measurements were made in acetonitrile solution using 0.1 M tetrabutylammonium hexafluorophosphate (Bu_4_NPF_6_) in acetonitrile as supporting electrolyte. The scan rate was 200 mV s^−1^. The glassy carbon electrode was used as a work electrode. Potentials were measured relative to a saturated calomel electrode (SCE). CV measurements for 10^−3^ M solutions of the compounds were done in a 1,2-dichlorobenzene/acetonitrile (1:4) mixture containing 0.1 M tetrabutylammonium hexafluorophosphate in a standard three-electrode cell equipped with a glass carbon working electrode (s = 2 mm^2^), a platinum plate as a counter electrode and an SCE as a reference electrode. The HOMO and the LUMO energies were evaluated using the first standard oxidation (*φ*_ox_) and reduction (*φ*_red_) potentials obtained from CV experiments as E_HOMO_ = −*e*(*φ*_ox_ + 4.40)(eV) and E_LUMO_ = −*e*(*φ*_red_ + 4.40)(eV), where *e* is the elementary charge [40,41].

#### 3.3.11. Theoretical Calculations

Frontier orbital configurations and S_1_→S_0_ transition energies in the gas phase were calculated using density functional theory (DFT) and time-dependent density functional theory (TDDFT). Initial geometries for all molecules were optimized in the ground state using DFT with the PBE0 exchange–correlation functional [42], the Ahlrichs def2-SVp basis set [43], and D3BJ dispersion corrections [44]. These optimized geometries were then used as starting points for TDDFT-based optimization of the S1 excited state, employing the same functional and basis set. Energies were subsequently refined using the def2-TZVp basis set [45]. All quantum chemical calculations were performed using the ORCA software package (version 6.0.1) [46].

#### 3.3.12. Solubility

The solubility of the compounds was determined using saturated solutions in THF obtained by stirring an excess amount of the solid material in the solvent. The compounds were gradually added in small portions to the pure solvent (5–50 mL) until saturation was achieved. The resulting saturated solutions were filtered through 0.22 µm nylon syringe filters (25 mm), after which the solvent was removed by evaporation. The remaining residue was then dried under vacuum at 80 °C to constant weight, and the obtained mass was used to calculate the solubility.

#### 3.3.13. X-Ray Diffraction

The 2D small- and wide-angle X-ray scattering (SWAXS) analysis of the samples was performed using BioMUR station of the Kurchatov synchrotron (National Research Center Kurchatov Institute). A 1.7 T bending magnet operating at an energy of 8 keV (1.445 Å), resolution δE/E of 10^−3^, and a photon flux of 10^9^ was used as a source of radiation. The beam size at a sample was 0.4 × 0.3 mm^2^; diffraction patterns were recorded with a Dectris Pilatus 1 M detector (Villigen, Switzerland). Sample-to-detector distance was approximately 150 mm, silver behenate and NaC(Na_2_Ca_3_Al_2_F_14_) were used as calibration standards, exposure time was 12 s. The Linkam THMS 600 (Salfords, UK) heating stage was employed for in situ temperature experiments with 7C/min rate and highest reachable temperature of 250 °C. Data integration and processing was performed by Fit_2_D V18.002 software by Andy Hammersly/ESRF (Grenoble, France). Further processing was carried out by Reflex module of Biovia Materials Studio 2023 software package V23.1.0.3829 (Dassault Systems, Waltham, MA, US). Crystal lattice indexing was performed using X-cell [47] and, further, Pawley refinement procedure.

The ID-PD sample was studied using TSWAXS camera S3-Micropix, manufactured by Hecus (Graz, Austria) (CuKa, l = 1.542 Å). Two detectors were used: two-dimensional Dectris (Villigen, Switzerland) Pilatus 100K and linear gas position sensitive detector PSD 50M operating at a pressure of 8 bar Ar/Me, the high-voltage and current at Xenocs (Grenoble, France) Genix generator were 50 kV and 1 mA. For shaping of X-ray beam the Fox 3D vacuum optics were used, and the slits in the Kratky collimator were set to 0.1 and 0.2 mm correspondingly. The angular scale was between 0.003 Å^−1^ and 1.9 Å^−1^. To calibrate small- and wide-angle diffractograms, silver behenate and lupolen (LDPE) calibrants were used as a reference. To eliminate the influence of air the X-ray optics system and camera was vacuumed to pressure (2 ÷ 3) × 10^−2^ mm Hg. The exposition time was 1200 s.

## 4. Conclusions

A novel series of small molecules incorporating indolo[3,2-b]indole (ID) and benzothieno[3,2-b]benzothiophene (BT) annulated units, bridged by a phenylene π-spacer with either alkyl or various electron-withdrawing groups (EWGs) has been synthesized. ID-based compounds exhibit enhanced electron-donating strength, reflected in elevated HOMO energy levels, reduced optical bandgaps, and pronounced bathochromic shifts in both absorption and emission spectra. Comprehensive theoretical calculations were found to be in close agreement with experimental observations, thereby reinforcing the structure–property relationships identified. Remarkably, ID derivatives achieve PLQY in solution as high as 92%, underscoring their better potential for emissive applications as compared to BD core. The substitution of the BT core with its nitrogen-rich ID analogue improves solubility and tailors phase behavior and crystal packing, critically influencing parameters essential for device fabrication. Thus, the annulated ID core emerges as a compelling platform for the development of medium bandgap luminescent and soluble organic semiconducting materials. The results of this study underscore the molecular tunability of annulated semiconductors via heteroatom incorporation and the use of side substituents with varying electron-accepting abilities. Building on this first systematic comparison of BD and ID derivatives, future research may focus on the targeted molecular engineering of annulated semiconductors to further advance the development of high-performance organic electronic devices.

## Figures and Tables

**Table 1:** Optical properties of the model and target compounds in dilute THF solutions and solid state.

Compound	THF Solution					Solid State		
	$\lambda_{abs}^{\max}$, nm	$\lambda_{fluor}^{\max}$, nm	υ_A_–υ_F_, cm^−1^	∆v = 0–0, cm^−1^	PLQY, %	$\lambda_{abs}^{\max}$, nm	$\lambda_{fluor}^{\max}$, nm	PLQY, %
ID	370^sh^, 354, 328, 314^sh^, 264, 240	385^sh^, 402, 423^sh^	1050	26,600	58	385, 367, 334, 321^sh^, 267	444, 456	38
ID-PD	384, 355, 294, 249	436, 453, 484^sh^	3050	24,000	76	428, 409, 362^sh^, 351	474, 497	18
ID-PCOD	418, 369, 322, 264	524	4840	21,400	92	483^sh^, 459, 372, 343^sh^	551, 585^sh^	56
ID-PDD	447, 383, 287	no fluor.	–	–	0	480, 382	no fluor.	0
BT	331, 316^sh^, 307, 295^sh^, 263, 255, 238	337, 352, 368^sh^	540	30,000	3	348, 333, 319^sh^	372, 388^sh^, 419, 444^sh^	1
BT-PD	356^sh^, 339, 278, 245	377, 397, 420^sh^	1570	27,100	38	399, 380, 362	448, 473, 504, 546^sh^	21
BT-PCOD	363, 345, 282, 247	409, 425^sh^	3100	25,600	19	422, 405, 382	467, 493^sh^	4
BT-PDD	382, 356^sh^, 274, 242^sh^	537	7560	22,500	10	402, 368	525	22

Notes: $\lambda_{abs}^{\max}$ is an absorption maximum wavelength; $\lambda_{fluor}^{\max}$ is an emission maximum wavelength; υ_A_–υ_F_ is a Stokes shift; PLQY is a photoluminescence quantum yield; ^sh^ is a shoulder of the spectra.

**Table 2:** Results of TDDFT calculations of condensed compounds.

Compound	Experiment (THF)			Calculated (PBE0)				Calculated	
	S_0_→S_1_, nm	f_osc_ (e)	f_osc_ (v)	S_0_→S_1_, nm	f_osc_ (e)	S_1_→S_0_, nm	f_osc_ (Fluor)	k_r_, ns^−1^	k_nr_, ns^−1^
ID	366	0.10	0.07	343.8	0.11	398.3	0.126	0.05	0.04
	328	0.21	0.24	299.4	0.23				
	266	0.91	0.72	251.7	0.90				
ID-PD	388	0.68	0.32	380.8	0.67	446.9	0.724	0.24	0.08
	347	0.73	0.68	332.9	0.73				
	296	0.52	0.33	291.3	0.52				
	290	0.33	0.24	283.2	0.33				
ID-PCOD	424	0.77	0.18	425.8	0.78	493.2	0.818	0.22	0.02
	370	0.86	0.60	361.1	0.88				
ID-PDD	461	0.82	0.40	512.7	0.80	630.5	0.384	0.06	–
	385	0.71	0.46	417.7	0.72				
	337	0.18	0.15	354.2	0.18				
	298	1.00	0.51	316.2	0.97				
BT	331	0.10	0.10	317.6	0.14	347.7	0.169	0.09	3.01
	307	0.31	0.36	290.3	0.34				
	264	0.32	0.31	255.9	0.30				
BT-PD	359	0.70	0.87	352.3	1.16	404.9	1.515	0.62	1.01
	324	0.45	0.43	320.4	0.42				
	280	0.37	0.33	274	0.33				
BT-PCOD	369	1.31	0.71	375.8	1.17	428.1	1.501	0.55	2.33
	333	0.56	0.29	336.7	0.56				
	329	0.07	–	332.1	0.07				
	284	0.07	–	286.8	0.07				
BT-PDD	395	1.32	0.91	430.6	1.18	487.5	1.533	0.43	3.87
	353	0.60	0.66	371.7	0.56				
	323	0.33	0.16	323	0.33				
	297	0.49	0.29	307.6	0.50				

Notes: f_osc_ is an oscillator force; (e) and (v) is an electronic and vibrational component of the transition, recently; k_r_ is a fluorescence constant k_nr_ is a nonradiative constant (k_nr_ = k_r_/Q − k_r_).

**Table 3:** Solubility and thermal stability data for the target compounds.

Compound	Solubility, g/L	*T*_d_, °C	
		in Air	in Inert
ID-PD	12	355	376
ID-PCOD	1.2	315	394
ID-PDD	28	372	397
BT-PD	0.5	271	385
BT-PCOD	0.2	319	421
BT-PDD	58	335	391

Notes: the solubility measured in THF at room temperature; *T*_d_ is the decomposition temperature calculated for the 5% weight-loss according to TGA.

**Table 4:** Crystallographic data obtained after indexing and refinement at varied temperatures.

Compound	T, °C	System	a, Å	b, Å	c, Å	α, deg	β, deg	γ, deg	Volume, Å^3^	Rwp	MM	Nmol	ρ, g/cm^−3^
ID-PD	25	Triclinic	10.5	17.7	22.3	71.0	82.3	80.6	3815	-	667	4	1.162
ID-PCOD	25	Triclinic	29.0	11.4	11.6	87.7	116.8	96.3	3380	7.2	723	3	1.066
	170	Monoclinic	51.1	5.28	41.3	90	113.3	90	10495	12.7	723	10	1.144
	180	Triclinic	39.3	17.1	5.36	92.0	100.8	92.2	3538	8.7	723	3	1.018
	210	Monoclinic	49.8	5.13	33.3	90	113.0	90	7831	16.8	723	7	1.074
ID-PDD	25	Hexagonal	38.3	10.5	10.5	120	90	90	3665	4.5	819	3	1.113
	250	Triclinic	38.0	15.5	6.9	88.9	98.6	114	3634	10.1	819	3	1.123
BT-PD	25	Monoclinic	41.8	9.4	11.4	90	85.2	90	4447	9.5	673	4	1.006
	70	Monoclinic	41.7	9.5	6.6	98.6	90	90	2584	9.8	673	2	0.865
	250	Monoclinic	40.2	5.0	10.4	90	98.5	90	2078	8.7	673	2	1.076
BT-PCOD	25	Monoclinic	55.0	9.4	9.1	108.5	90	90	4426	8.0	729	4	1.094
	220	Monoclinic	44.8	11.7	9.0	105.9	90	90	4557	4.2	729	4	1.063
	230	Monoclinic	41.4	5.94	21.1	90	99.8	90	5109	7.5	729	4	0.948
BT-PDD	25	Triclinic	37.8	9.1	8.3	111.3	88.2	110.8	2497	10.0	825	2	1.098
	220	Monoclinic	32.8	6.0	27.1	90	102.3	90	5248	6.7	825	4	1.045

## Data Availability

The data supporting this work is in the article and Supplementary Materials.

## Supplementary Materials

The following supporting information can be downloaded at: https://www.mdpi.com/article/10.3390/molecules31122046/s1, Figure S1: ^1^H NMR spectrum of compound 1 in Chloroform-d; Figure S2: ^1^H NMR spectrum of compound ID in Chloroform-d; Figure S3: ^1^H NMR spectrum of compound 3 in Chloroform-d; Figure S4: ^13^C NMR spectrum of compound 3 in Chloroform-d; Figure S5: ^1^H NMR spectrum of compound 4 in Chloroform-d; Figure S6: ^13^C NMR spectrum of compound 4 in Chloroform-d; Figure S7: ^1^H NMR spectrum of compound 5 in Chloroform-d; Figure S8: ^13^C NMR spectrum of compound 5 in Chloroform-d; Figure S9: ^1^H NMR spectrum of compound 6 in Chloroform-d; Figure S10: ^13^C NMR spectrum of compound 6 in Chloroform-d; Figure S11: ^1^H NMR spectrum of compound 7 in Acetone-d_6_; Figure S12: ^13^C NMR spectrum of compound 7 in Acetone-d_6_; Figure S13: ^1^H NMR spectrum of compound 8 in Chloroform-d; Figure S14: ^1^H NMR spectrum of compound 9 in Chloroform-d; Figure S15: ^1^H NMR spectrum fragment of compound ID-PD in Acetone-d_6_; Figure S16: MS spectra for compound ID-PD; Figure S17: ^1^H NMR spectrum of compound BT-PD in Chloroform-d; Figure S18: MS spectra for compound BT-PD; Figure S19: ^1^H NMR spectrum of compound ID-PCOD in Chloroform-d; Figure S20: ^13^C NMR spectrum of compound ID-PCOD in Chloroform-d; Figure S21: MS spectra for compound ID-PCOD; Figure S22: ^1^H NMR spectrum of compound BT-PCOD in Chloroform-d; Figure S23: MS spectra for compound BT-PCOD; Figure S24: ^1^H NMR spectrum of compound ID-PDD in Chloroform-d; Figure S25: ^13^C NMR spectrum of compound ID-PDD in Chloroform-d; Figure S26: MS spectra for compound ID-PDD; Figure S27: ^1^H NMR spectrum of compound BT-PDD in Chloroform-d; Figure S28: MS spectra for compound BT-PDD; Figure S29: HOMO and LUMO of model and target compounds; Figure S30: Mirror symmetry in the absorption and emission spectra of condensed compound solutions based on ID and BT cores in THF; Figure S31: Normalized absorption spectra of thin polycrystalline films of condensed compounds with ID (a) and BT (c) cores. Normalized fluorescence spectra of condensed compound powders with ID (b) and BT (d) cores; Figure S32: Cyclic voltammograms of the model and target compounds; Figure S33: TGA curves in air (a) and under (b) inert atmosphere for the obtained compounds; Figure S34: DSC curves for the first heating (a), cooling (b) and second heating (c); Figure S35: Optical polarizing microscopy of the target compound; Figure S36: TWAXS plot of ID-PDD at heating rate of 7 C/min; Figure S37: TWAXS plot of BT-PDD at heating rate of 7 C/min; Figure S38: TWAXS plot of BT-PD at heating rate of 7 C/min; Figure S39: TWAXS plot of ID-PCOD at heating rate of 7 C/min; Figure S40: TWAXS plot of BT-PCOD at heating rate of 7 C/min; Figure S41: TWAXS plot of ID-PD at various temperature; Table S1: Electrochemical properties and theoretical DFT data of the model and target compounds; Table S2: Phase transitions found on the DSC curves of target compounds.

## Abbreviations

The following abbreviations are used in this manuscript:

A	Acceptor
BT	Benzothieno[3,2-b]benzothiophene
BT-PCOD	1,1′-([1]benzothieno[3,2-b][1]benzothiene-2,7-diylbis(4,1-phenylene))bis(undecan-1-one)
BT-PD	2,7-bis(4-decylphenyl)[1]benzothieno[3,2-b][1]benzothiophene
BT-PDD	2,2′-([1]benzothieno[3,2-b][1]benzothiene-2,7-diylbis(4,1-phenyleneundec-1-yl-1-ylidene))dimalononitrile
CV	Cyclic voltammetry
D	Donor
DFT	Density functional theory
DSC	Differential scanning calorimetry
EWG	Electron-withdrawing group
GPC	Gel permeation chromatography
HOMO	Highest occupied molecular orbital
ID	Indolo[3,2-b]indole
ID-PCOD	1,1′-[(5,10-dimethyl-5,10-dihydroindolo[3,2-b]indole-2,7-diyl)bis(4,1-phenylene)]diundecan-1-one
ID-PD	2,7-bis(4-decylphenyl)-5,10-dimethyl-5,10-dihydroindolo[3,2-b]indole
ID-PDD	2,2′-[(5,10-dimethyl-5,10-dihydroindolo[3,2-b]indole-2,7-diyl)bis(4,1-phenyleneundec-1-yl-1-ylidene)]dimalononitrile
IPTMDOB	2-isopropoxy-4,4,5,5-tetramethyl-1,3,2-dioxaborolane
LUMO	Lowest unoccupied molecular orbital
MALDI-TOF	Matrix-assisted laser desorption/ionization time-of-flight mass spectrometry
OFET	Organic field-effect transistor
OLED	Organic light-emitting diode
PLQY	Photoluminescence quantum yield
POM	Polarized optical microscopy
SCE	Saturated calomel electrode
SWAXS	Small- and wide-angle X-ray scattering
TDDFT	Time-dependent density functional theory
TGA	Thermogravimetric analysis
THF	Tetrahydrofuran
XRD	X-ray diffraction
