# Supplementary material for: Donor-Acceptor Derivatives of Indolo[3,2-b]indole and Benzothieno[3,2-b]benzothiophene: Similar Annulated Structures but Divergent Properties
Source: Molecules. 2026 Jun 11;31(12):2046. doi: 10.3390/molecules31122046 (PMC13306162; doi:10.3390/molecules31122046)
Supplement: Supplementary file 1 [file molecules-31-02046-s001.zip › molecules-4328290-supplementary.pdf]

ELECTRONIC SUPPORTING INFORMATION

Donor-Acceptor Derivatives of Indolo[3,2-b]indole and Benzothieno[3,2-b]benzothiophene: Similar Annulated Structures but Divergent Properties

Liya A. Poletavkina [1,2], Ivan V. Dyadishchev [1], Artem V. Bakirov [1,3], Evgenia A. Svidchenko [1], Nikolay M. Surin [1], Nikita O. Dubinets [1,3], Dmitry O. Balakirev [1], Svetlana M. Peregudova [1,4], George V. Cherkaev [1], Irina A. Chuyko [1,2], Sergei N. Chvalun [1,3] and Yuriy N. Luponosov [1,*]

[1]Enikolopov Institute of Synthetic Polymeric Materials, Russian Academy of Sciences, Profsoyuznaya St. 70, Moscow 117393, Russia

[2]Federal Research Center for Problems of Chemical Physics and Medicinal Chemistry, Russian Academy of Sciences, Semenov Ave. 1, Chernogolovka, Moscow 142432, Russia

[3]National Research Center "Kurchatov Institute", Kurchatov Sq. 1, Moscow 123182, Russia

[4]Nesmeyanov Institute of Organoelement Compounds of the Russian Academy of Sciences, Vavilova St. 28, Moscow 119991, Russia

*Correspondence: luponosov@ispm.ru

CONTENTS

1. ^{1}H, ^{13}C NMR and MS Spectra S2
2. Optical Data S29
3. CV Data S31
4. TGA and DSC Data S33
5. X-ray Data S35

1. ^{1}H, ^{13}C NMR and MS Spectra

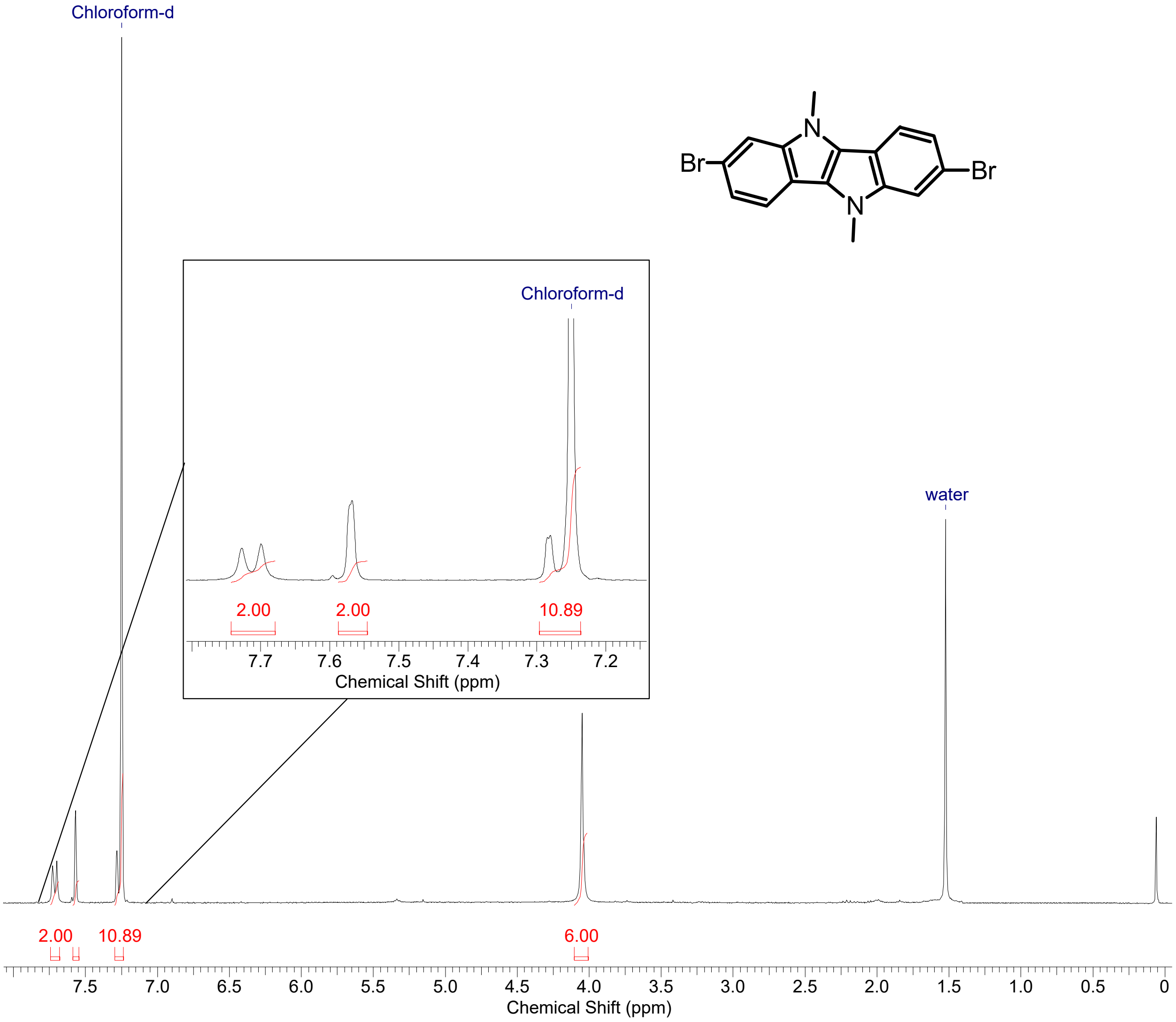

No.	Annotation	(ppm)
1	water	1.52
2	Chloroform-d	7.25

No.	(ppm)	Value	Absolute Value
1	[4.01 .. 4.11]	6.000	3.79572e+9
2	[7.24 .. 7.30]	10.894	6.89121e+9
3	[7.55 .. 7.59]	2.000	1.26514e+9
4	[7.68 .. 7.74]	2.004	1.26769e+9

Figure S1. ^{1}H NMR spectrum of compound **1** in Chloroform-d.

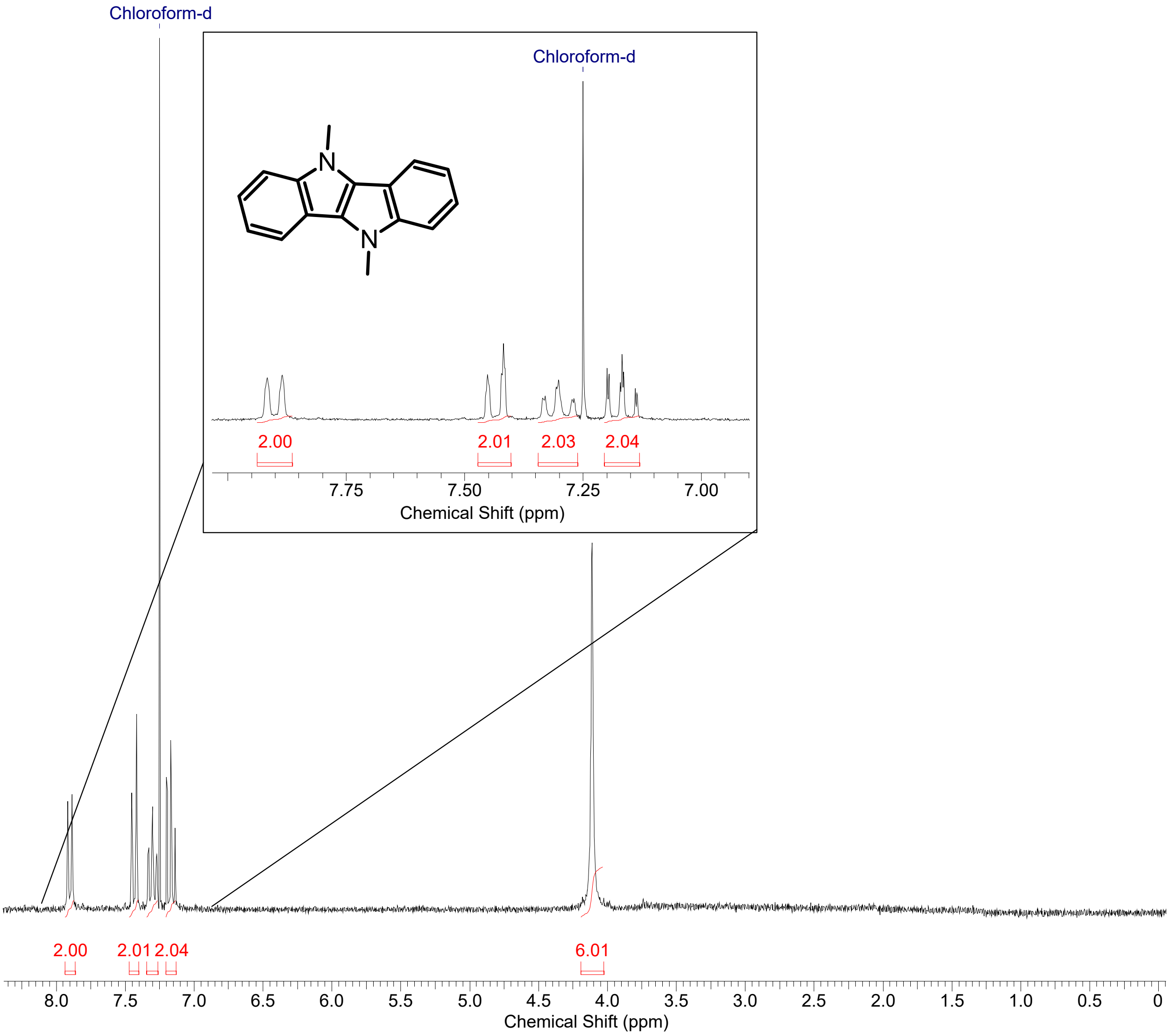

No.	Annotation	(ppm)
1	Chloroform-d	7.25

No.	(ppm)	Value	Absolute Value
1	[4.03 .. 4.19]	6.006	3.99834e+6
2	[7.13 .. 7.20]	2.045	1.36113e+6
3	[7.26 .. 7.35]	2.030	1.35139e+6
4	[7.40 .. 7.47]	2.010	1.33832e+6
5	[7.86 .. 7.94]	2.000	1.33139e+6

Figure S2. ^{1}H NMR spectrum of compound **ID** in Chloroform-d.

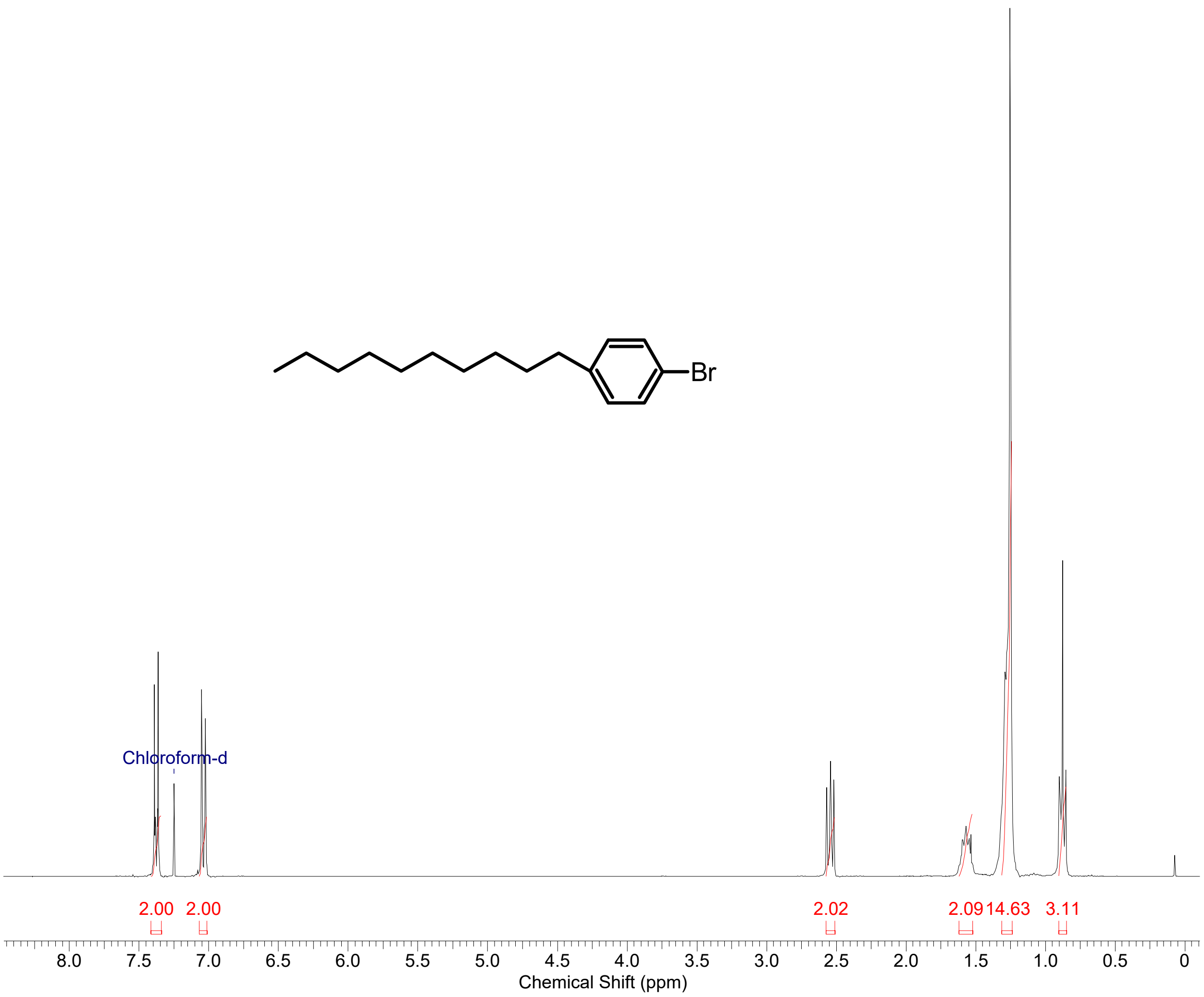

No.	Annotation	(ppm)
1	Chloroform-d	7.25

No.	(ppm)	Value	Absolute Value
1	[0.85 .. 0.90]	3.115	2.78033e+9
2	[1.24 .. 1.31]	14.629	1.30580e+10
3	[1.52 .. 1.62]	2.092	1.86716e+9
4	[2.51 .. 2.57]	2.018	1.80157e+9
5	[7.01 .. 7.07]	1.996	1.78164e+9
6	[7.34 .. 7.41]	2.000	1.78525e+9

Figure S3. ^{1}H NMR spectrum of compound **3** in Chloroform-d.

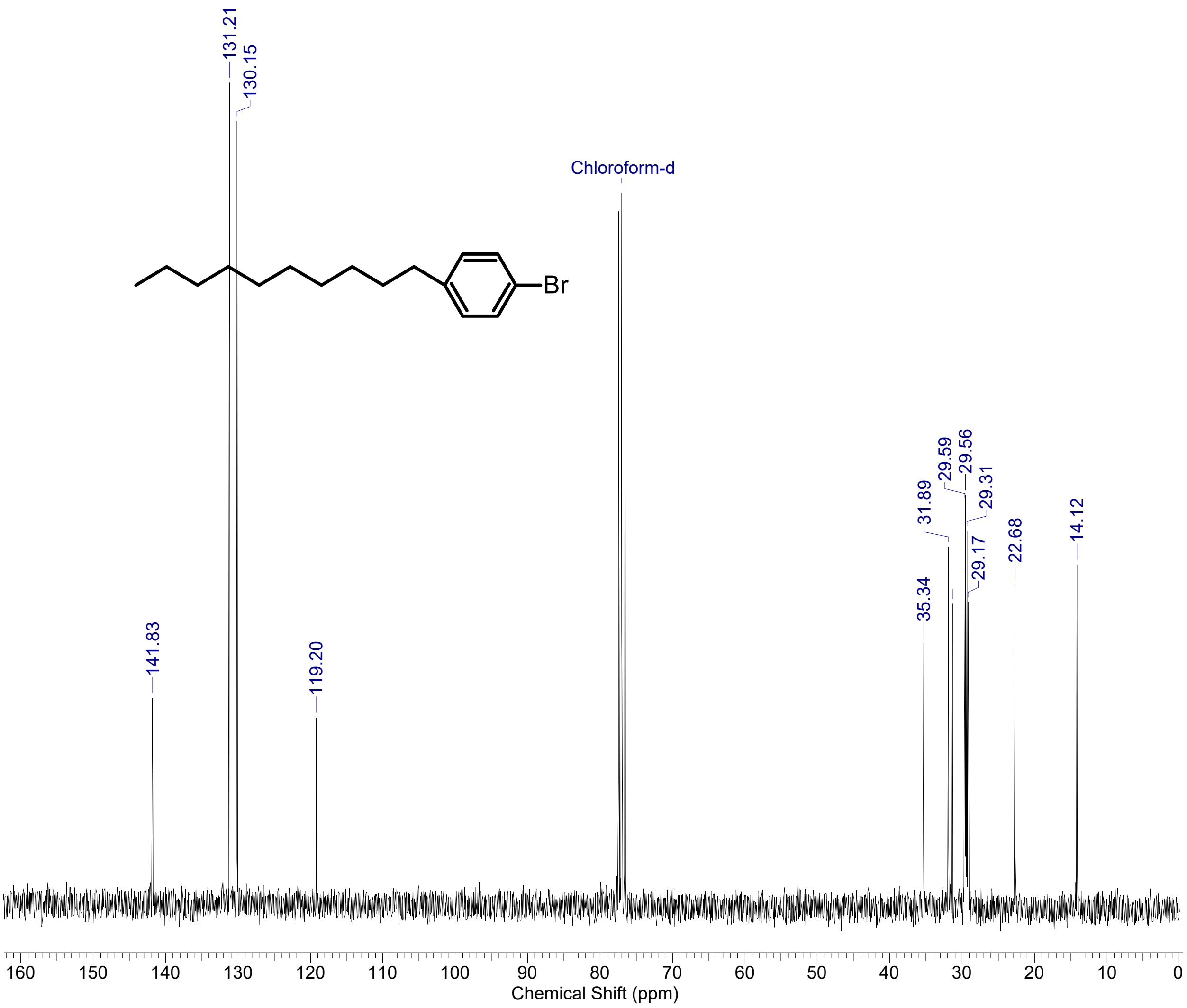

No.	(ppm)	(Hz)	Height
1	14.12	1065.4	0.4221
2	22.68	1711.7	0.3984
3	29.17	2201.5	0.3773
4	29.31	2212.6	0.4627
5	29.45	2223.1	0.4147
6	29.56	2230.8	0.5055
7	29.59	2233.6	0.4956
8	31.33	2364.6	0.3753
9	31.89	2406.7	0.4438
10	35.34	2667.1	0.3277
11	119.20	8996.9	0.2392
12	130.15	9823.4	0.9539
13	131.21	9903.6	1.0000
14	141.83	10705.3	0.2625

No.	Annotation	(ppm)
1	Chloroform-d	77.00

Figure S4. ^{13}C NMR spectrum of compound **3** in Chloroform-d.

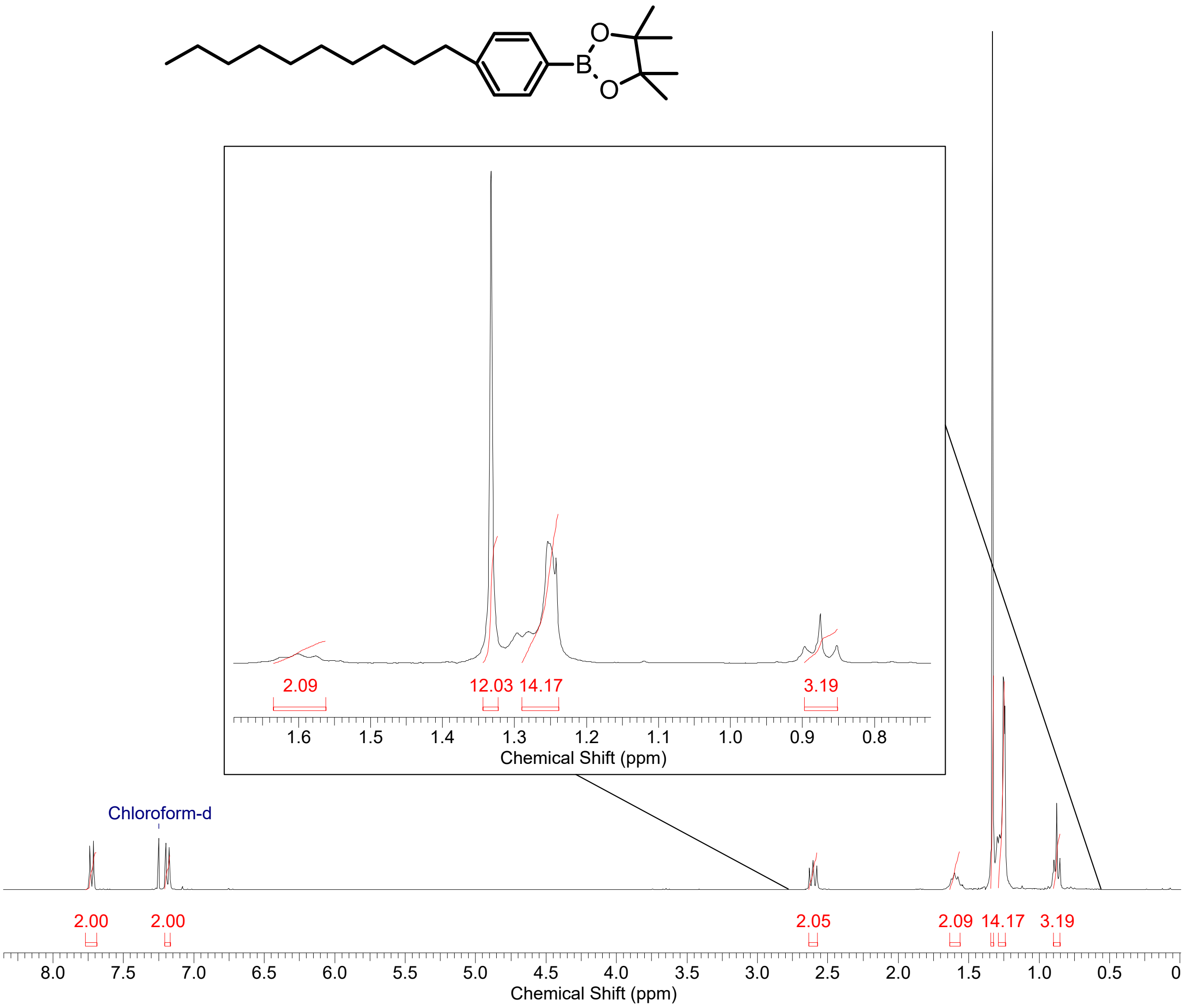

No.	Annotation	(ppm)
1	Chloroform-d	7.25

No.	(ppm)	Value	Absolute Value
1	[0.85 .. 0.90]	3.189	9.88971e+8
2	[1.24 .. 1.29]	14.171	4.39520e+9
3	[1.32 .. 1.34]	12.031	3.73145e+9
4	[1.56 .. 1.63]	2.085	6.46782e+8
5	[2.57 .. 2.64]	2.047	6.34775e+8
6	[7.17 .. 7.21]	2.001	6.20653e+8
7	[7.69 .. 7.77]	2.000	6.20306e+8

Figure S5. ^{1}H NMR spectrum of compound **4** in Chloroform-d.

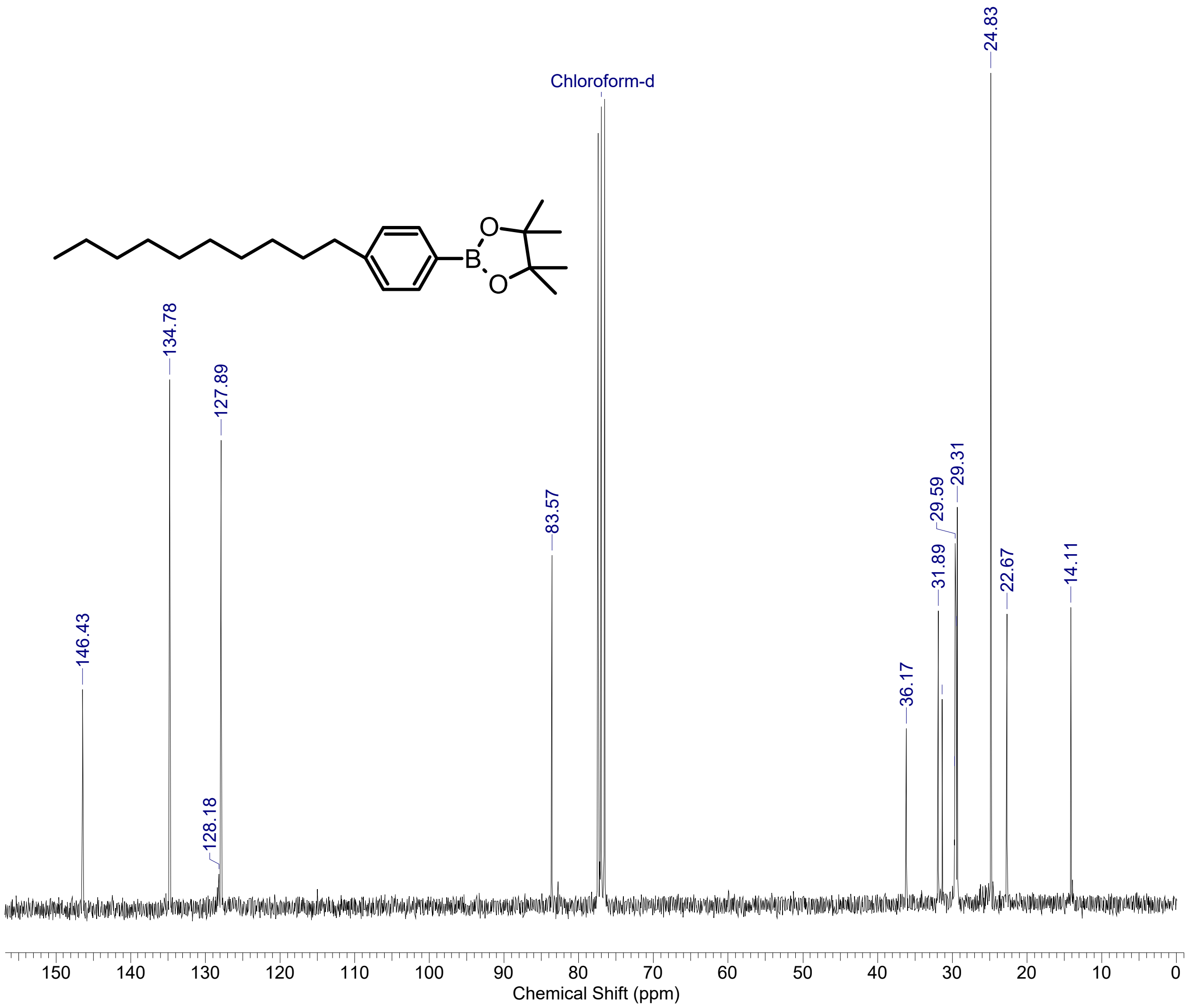

No.	(ppm)	(Hz)	Height
1	14.11	1064.8	0.3680
2	22.67	1711.1	0.3601
3	24.83	1874.2	1.0000
4	29.31	2212.0	0.4864
5	29.48	2225.3	0.3406
6	29.56	2231.4	0.3939
7	29.59	2233.6	0.4436
8	29.69	2240.8	0.1745
9	31.34	2365.2	0.2593
10	31.89	2406.7	0.3638
11	36.17	2730.1	0.2249
12	83.57	6307.7	0.4297
13	127.89	9652.6	0.5655
14	128.18	9674.7	0.0530
15	134.78	10172.8	0.6376
16	146.43	11051.9	0.2707

No.	Annotation	(ppm)
1	Chloroform-d	77.00

Figure S6. ^{13}C NMR spectrum of compound **4** in Chloroform-d.

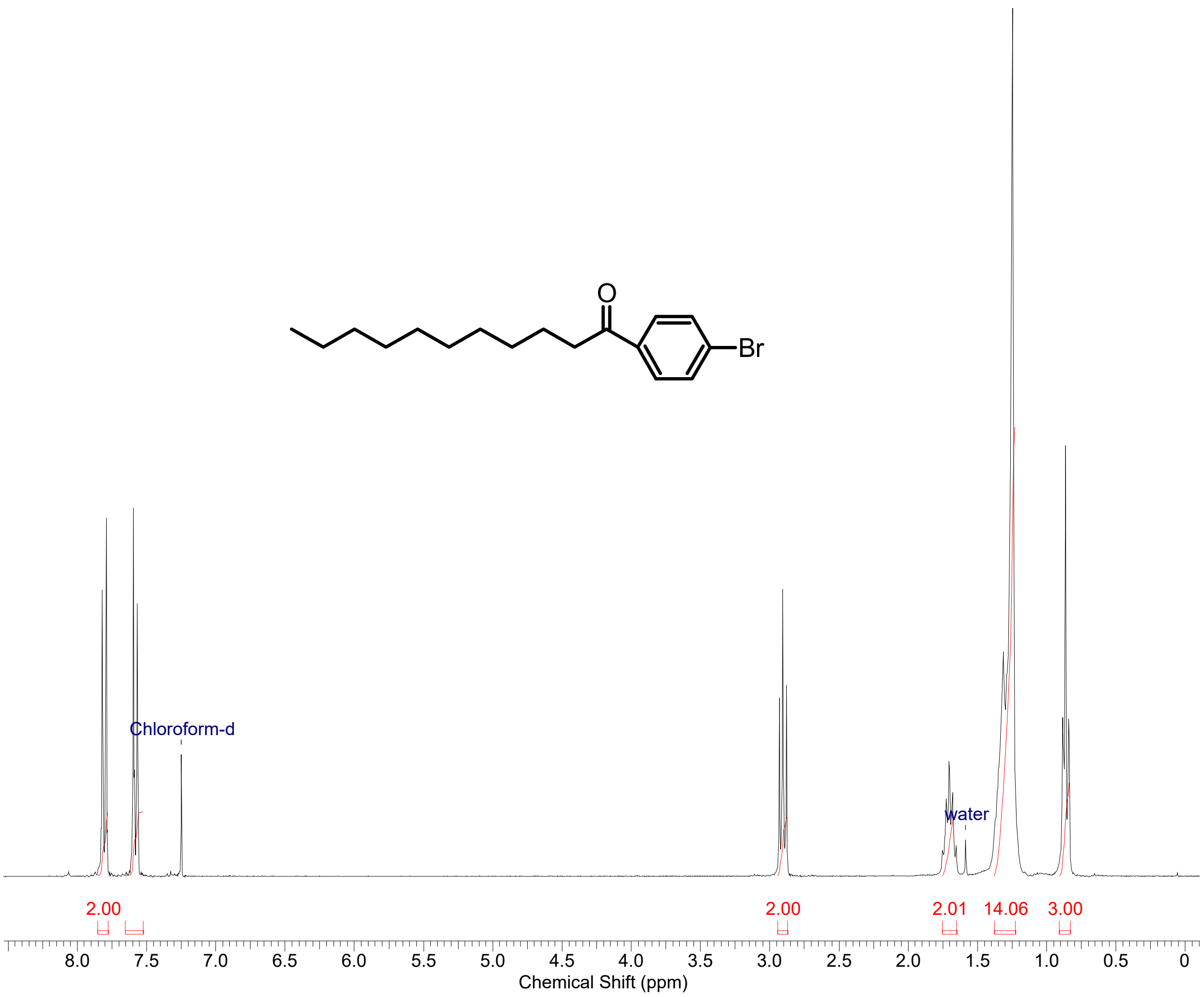

No.	Annotation	(ppm)
1	water	1.59
2	Chloroform-d	7.25

No.	(ppm)	Value	Absolute Value
1	[0.83 .. 0.91]	3.004	8.22311e+9
2	[1.23 .. 1.38]	14.061	3.84955e+10
3	[1.65 .. 1.75]	2.012	5.50764e+9
4	[2.87 .. 2.94]	2.002	5.48205e+9
5	[7.53 .. 7.65]	1.999	5.47250e+9
6	[7.78 .. 7.85]	2.000	5.47564e+9

Figure S7. ^{1}H NMR spectrum of compound **5** in Chloroform-d.

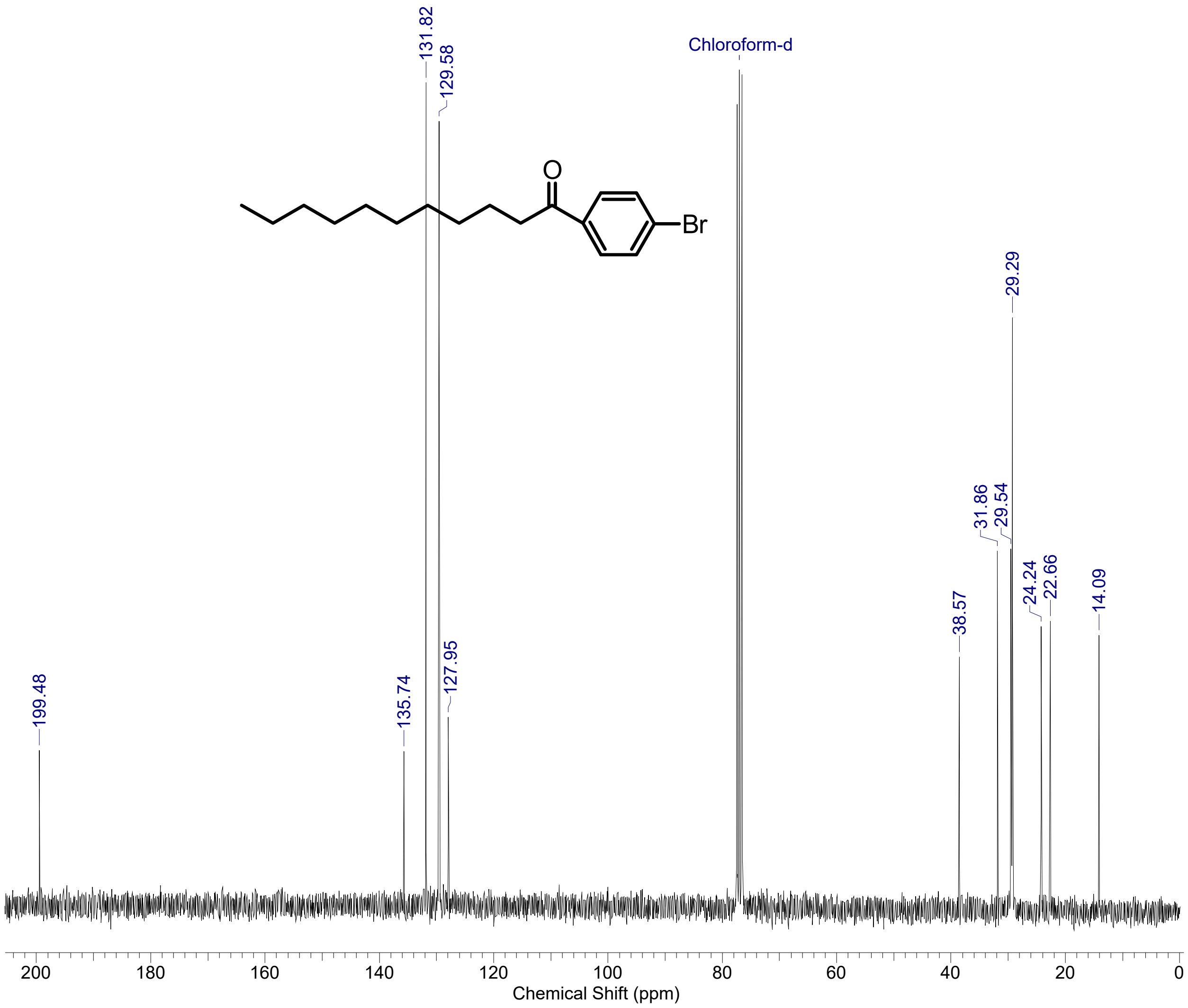

No.	(ppm)	(Hz)	Height
1	14.09	1063.7	0.3389
2	22.66	1710.0	0.3551
3	24.24	1829.5	0.3490
4	29.29	2210.9	0.7099
5	29.43	2221.4	0.4292
6	29.47	2224.2	0.4601
7	29.54	2229.7	0.4398
8	31.86	2405.0	0.4374
9	38.57	2910.9	0.3137
10	127.95	9657.6	0.2430
11	129.58	9780.3	0.9393
12	131.82	9949.5	0.9845
13	135.74	10245.3	0.2028
14	199.48	15056.4	0.2041

No.	Annotation	(ppm)
1	Chloroform-d	77.00

Figure S8. ^{13}C NMR spectrum of compound **5** in Chloroform-d.

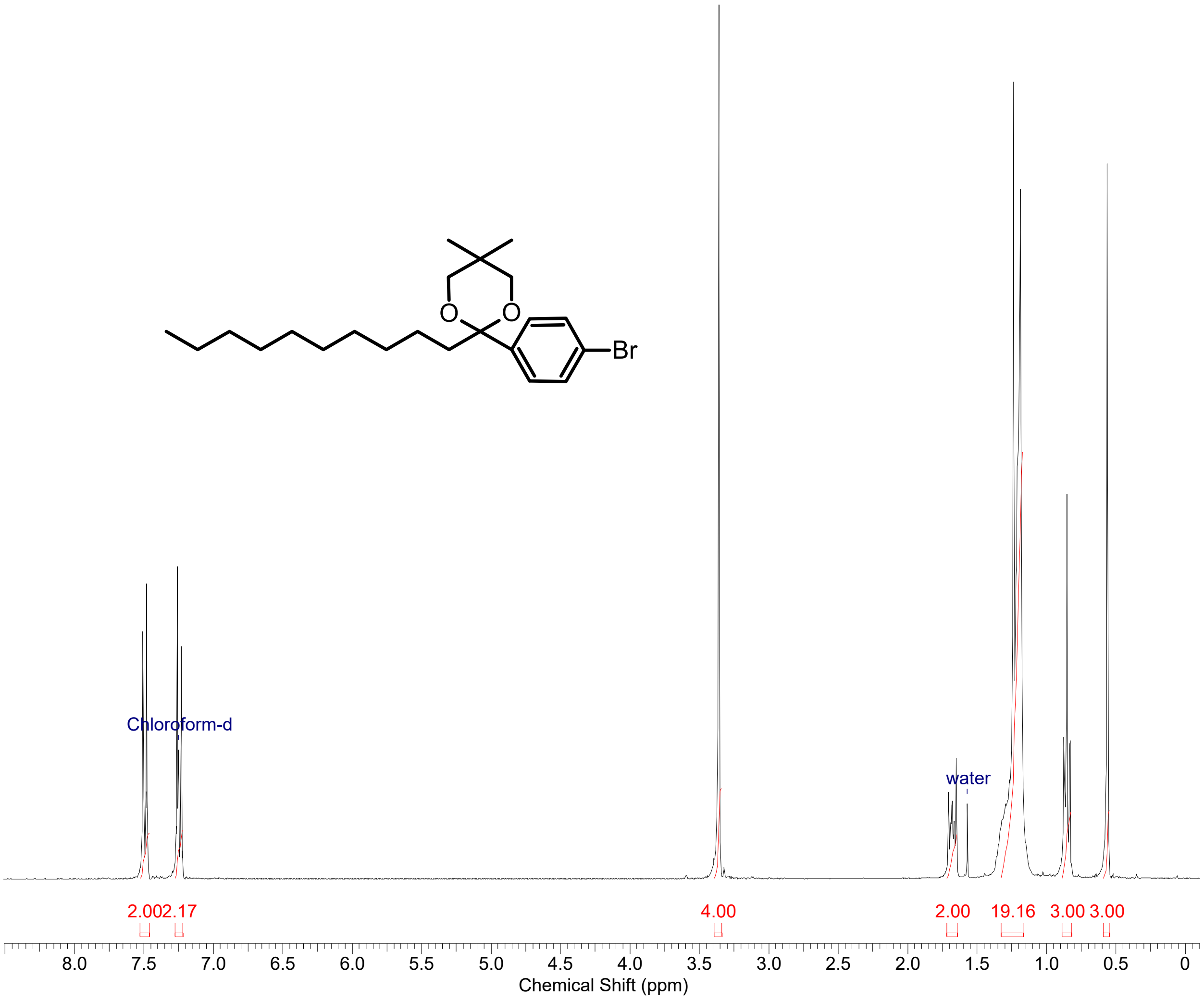

No.	Annotation	(ppm)
1	water	1.57
2	Chloroform-d	7.25

No.	(ppm)	Value	Absolute Value
1	[0.55 .. 0.59]	3.004	3.69411e+9
2	[0.82 .. 0.89]	3.001	3.68954e+9
3	[1.17 .. 1.33]	19.156	2.35522e+10
4	[1.64 .. 1.72]	2.001	2.45978e+9
5	[3.34 .. 3.40]	4.004	4.92312e+9
6	[7.22 .. 7.28]	2.170	2.66765e+9
7	[7.46 .. 7.53]	2.000	2.45910e+9

Figure S9. ^{1}H NMR spectrum of compound **6** in Chloroform-d.

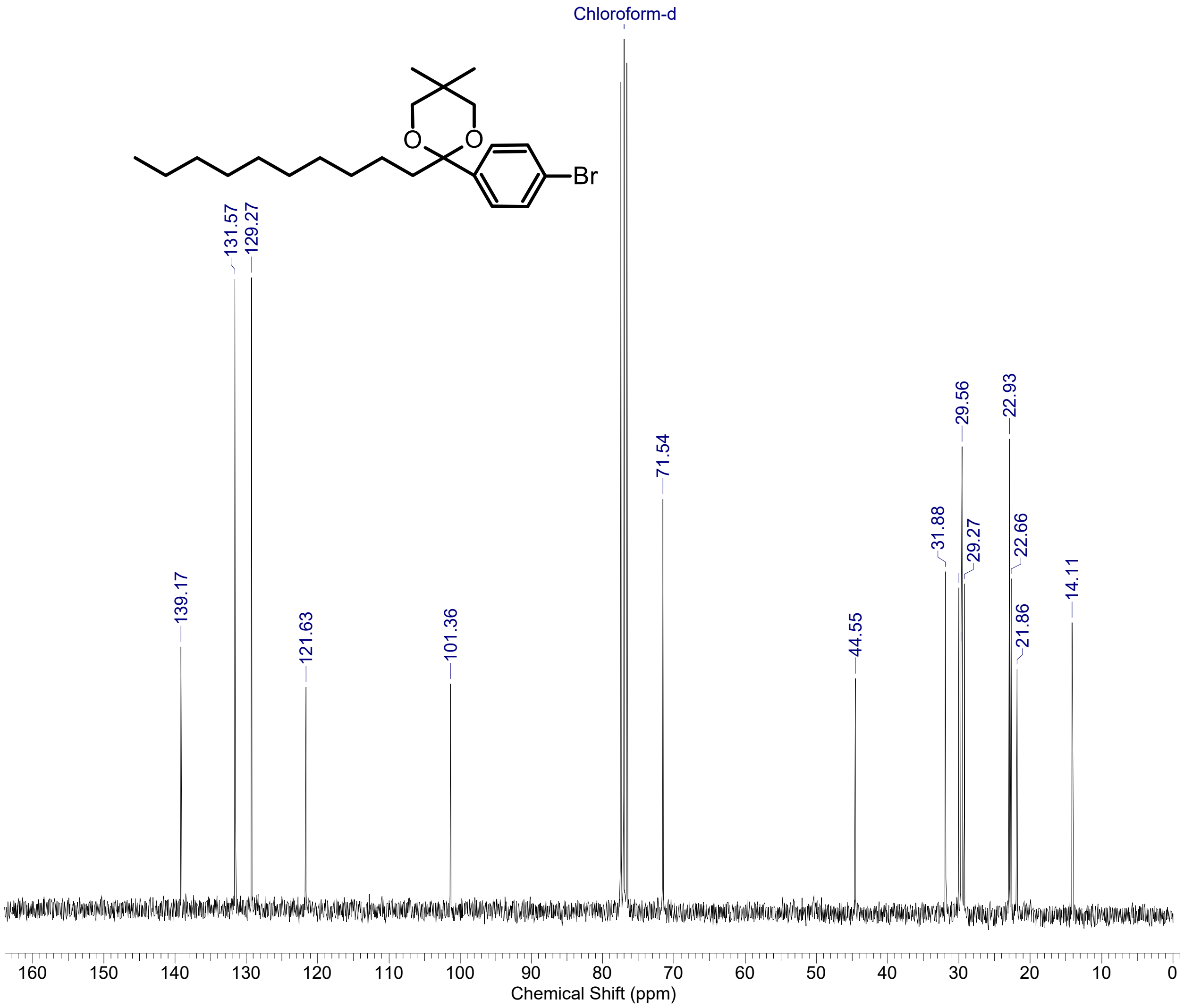

No.	(ppm)	(Hz)	Height
1	14.11	1064.8	0.3432
2	21.86	1649.8	0.2903
3	22.66	1710.6	0.3925
4	22.93	1731.0	0.5493
5	29.27	2209.3	0.3864
6	29.54	2229.7	0.5172
7	29.56	2230.8	0.5407
8	29.59	2233.1	0.4385
9	29.70	2241.9	0.3163
10	30.04	2267.3	0.3824
11	31.88	2406.1	0.4004
12	44.55	3362.6	0.2804
13	71.54	5399.3	0.4817
14	101.36	7650.6	0.2745
15	121.63	9180.4	0.2706
16	129.27	9757.1	0.7310
17	131.57	9930.7	0.7294
18	139.17	10504.0	0.3160

No.	Annotation	(ppm)
1	Chloroform-d	77.00

Figure S10. ^{13}C NMR spectrum of compound **6** in Chloroform-d.

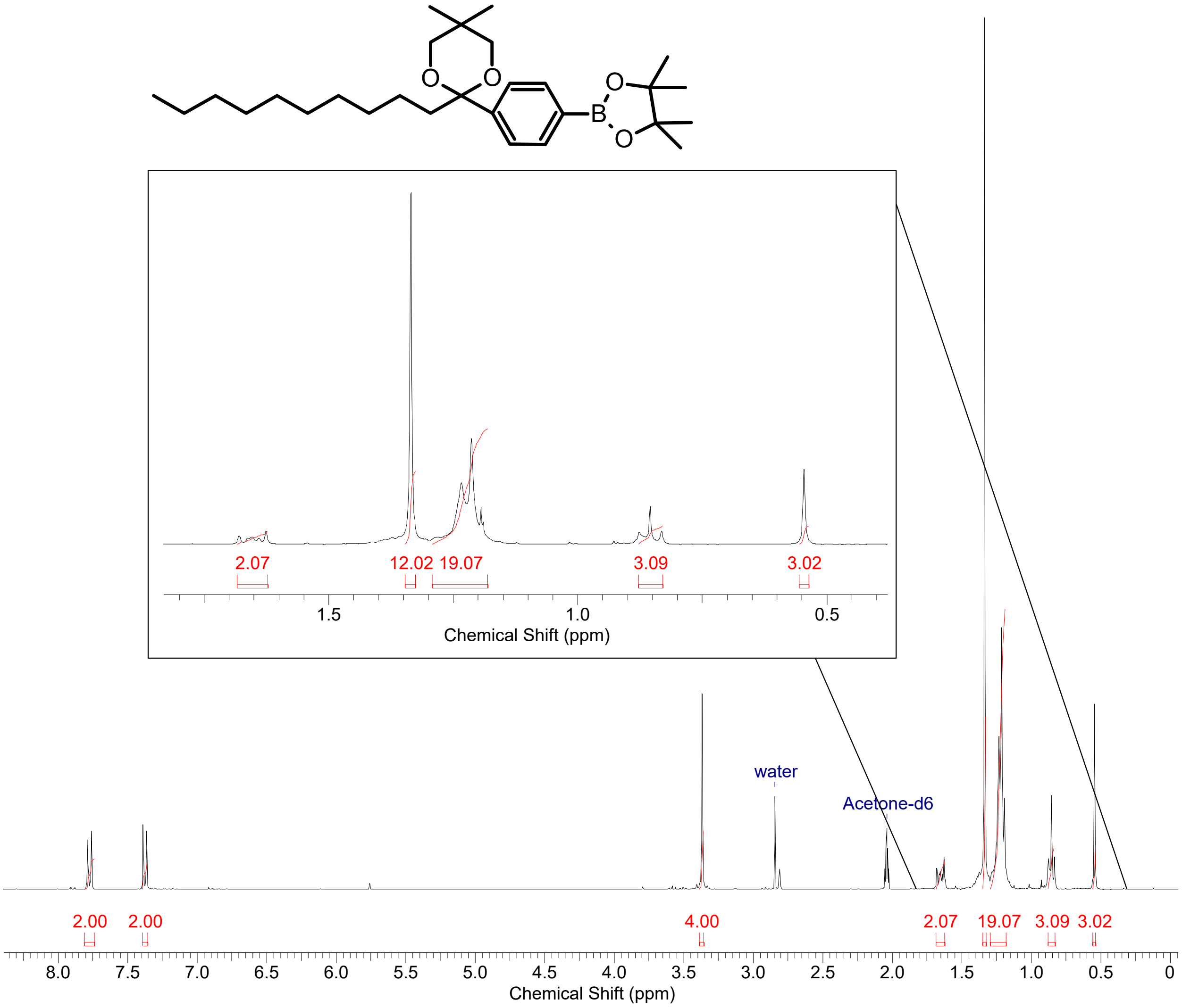

No.	Annotation	(ppm)
1	Acetone-d6	2.04
2	water	2.84

No.	(ppm)	Value	Absolute Value
1	[0.54 .. 0.56]	3.018	7.08555e+8
2	[0.83 .. 0.88]	3.089	7.25185e+8
3	[1.18 .. 1.29]	19.068	4.47680e+9
4	[1.33 .. 1.35]	12.020	2.82205e+9
5	[1.62 .. 1.68]	2.073	4.86721e+8
6	[3.35 .. 3.39]	4.004	9.40071e+8
7	[7.36 .. 7.39]	2.003	4.70357e+8
8	[7.74 .. 7.81]	2.000	4.69568e+8

Figure S11. ^{1}H NMR spectrum of compound **7** in Acetone-d$_6$.

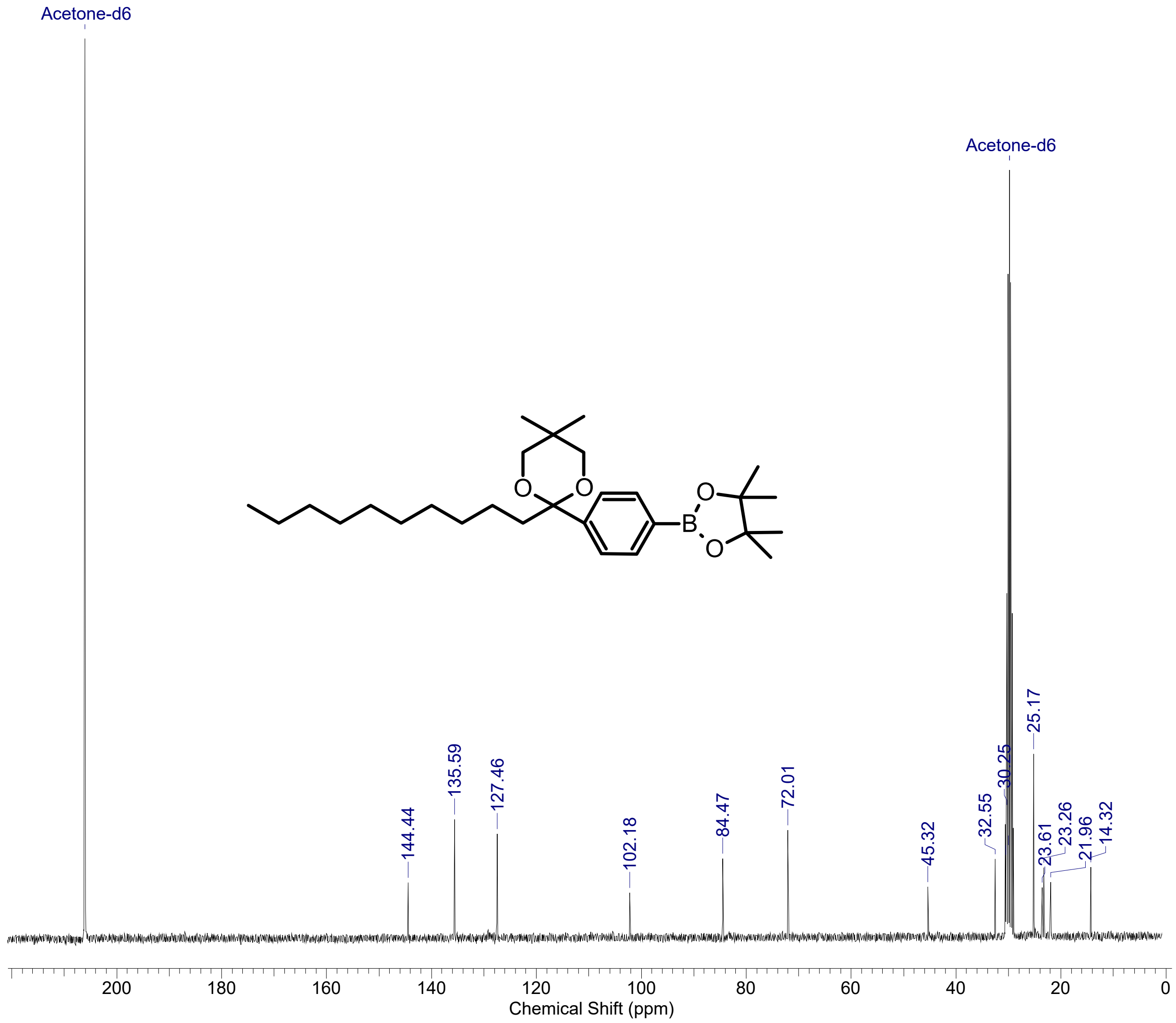

No.	(ppm)	(Hz)	Height
1	14.32	1081.0	0.0778
2	21.96	1657.7	0.0608
3	23.19	1750.5	0.0586
4	23.26	1755.5	0.0771
5	23.61	1782.1	0.0549
6	25.17	1899.8	0.2036
7	29.97	2262.0	0.0969
8	30.23	2281.9	0.1218
9	30.25	2283.5	0.1414
10	30.40	2294.6	0.0709

No.	(ppm)	(Hz)	Height
11	30.47	2299.6	0.0861
12	32.55	2457.1	0.0863
13	45.32	3420.8	0.0555
14	72.01	5434.9	0.1186
15	84.47	6375.3	0.0872
16	102.18	7712.2	0.0492
17	127.46	9620.7	0.1144
18	135.59	10233.8	0.1309
19	144.44	10902.2	0.0602

No.	Annotation	(ppm)
1	Acetone-d6	29.80
2	Acetone-d6	206.09

Figure S12. ^{13}C NMR spectrum of compound **7** in Acetone-d$_6$.

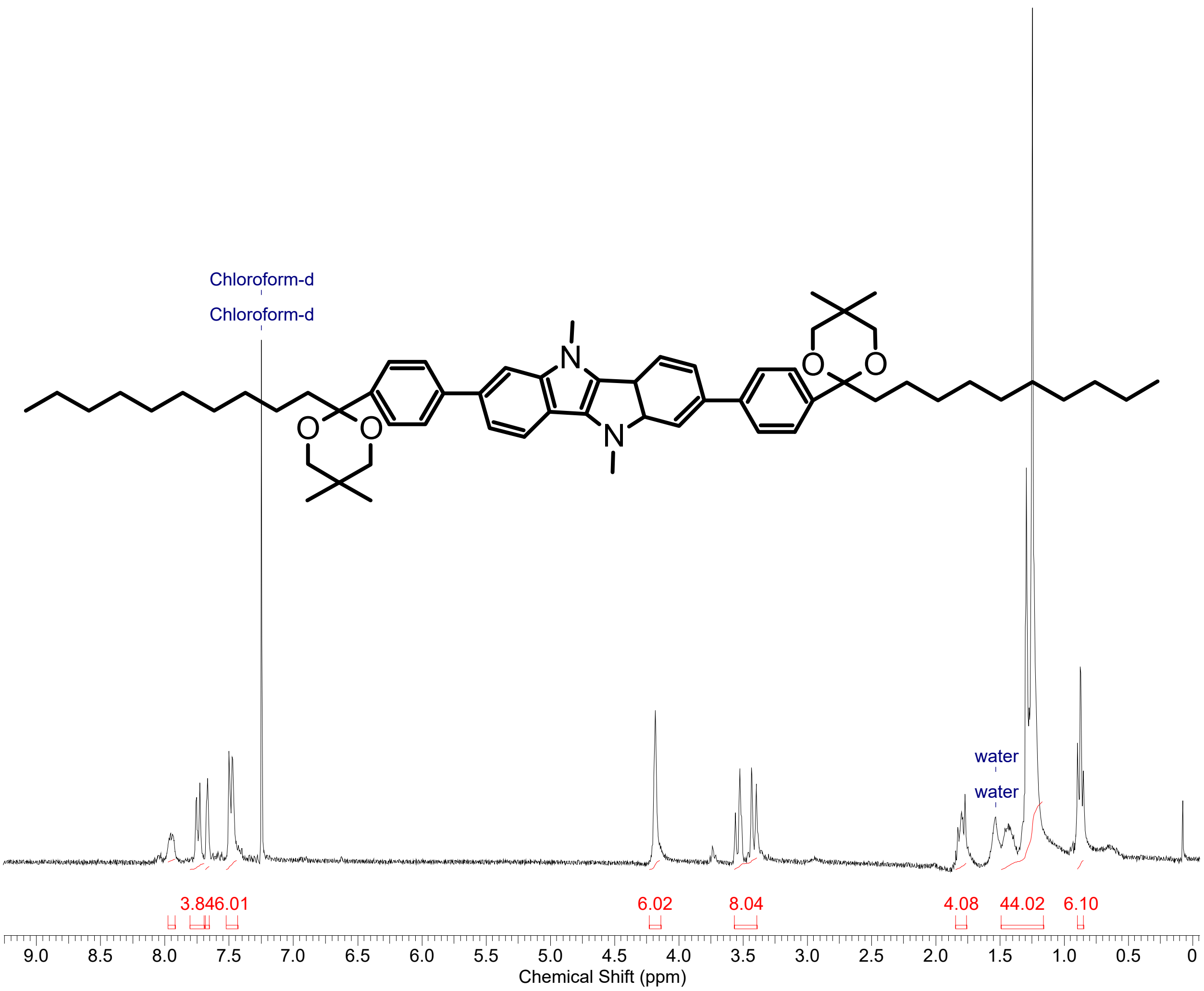

No.	Annotation	(ppm)
1	water	1.53
2	water	1.53
3	Chloroform-d	7.25
4	Chloroform-d	7.25

No.	(ppm)	Value	Absolute Value
1	[0.85 .. 0.90]	6.101	1.71504e+9
2	[1.16 .. 1.49]	44.020	1.23752e+10
3	[1.76 .. 1.84]	4.084	1.14804e+9
4	[3.39 .. 3.57]	8.043	2.26114e+9
5	[4.14 .. 4.23]	6.021	1.69265e+9
6	[7.44 .. 7.52]	6.015	1.69086e+9
7	[7.66 .. 7.69]	2.013	5.65986e+8
8	[7.69 .. 7.80]	3.837	1.07860e+9
9	[7.92 .. 7.98]	1.997	5.61296e+8

Figure S13. ^{1}H NMR spectrum of compound **8** in Chloroform-d.

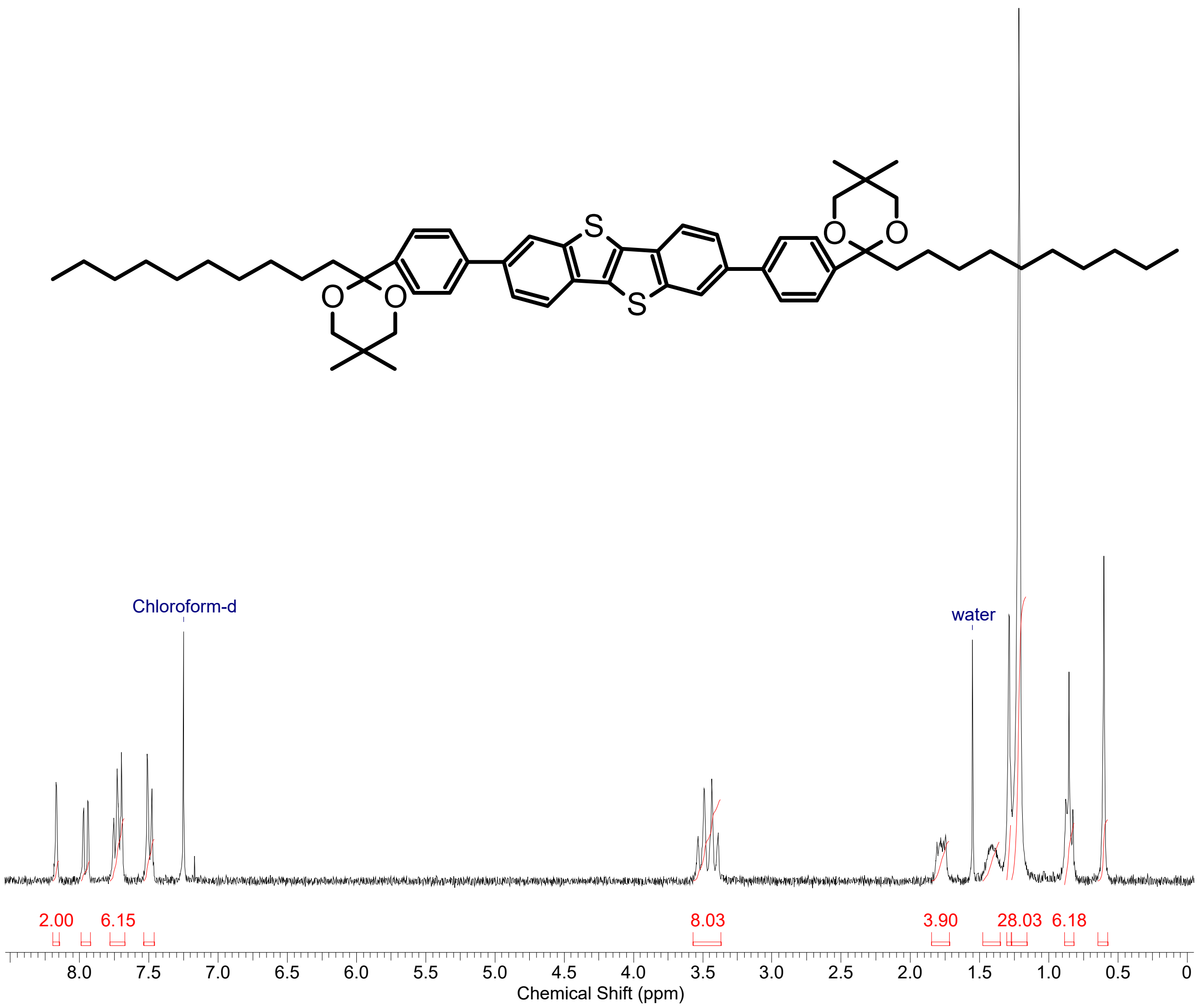

No.	Annotation	(ppm)
1	water	1.55
2	Chloroform-d	7.25

No.	(ppm)	Value	Absolute Value
1	[0.57 .. 0.64]	6.013	1.18607e+7
2	[0.82 .. 0.89]	6.181	1.21903e+7
3	[1.16 .. 1.27]	28.029	5.52836e+7
4	[1.27 .. 1.30]	6.081	1.19936e+7
5	[1.35 .. 1.48]	4.005	7.90028e+6
6	[1.72 .. 1.85]	3.898	7.68807e+6
7	[3.37 .. 3.57]	8.026	1.58297e+7
8	[7.46 .. 7.54]	4.086	8.05930e+6
9	[7.67 .. 7.78]	6.153	1.21355e+7
10	[7.92 .. 7.99]	2.001	3.94727e+6
11	[8.15 .. 8.19]	2.000	3.94467e+6

Figure S14. ^{1}H NMR spectrum of compound **9** in Chloroform-d.

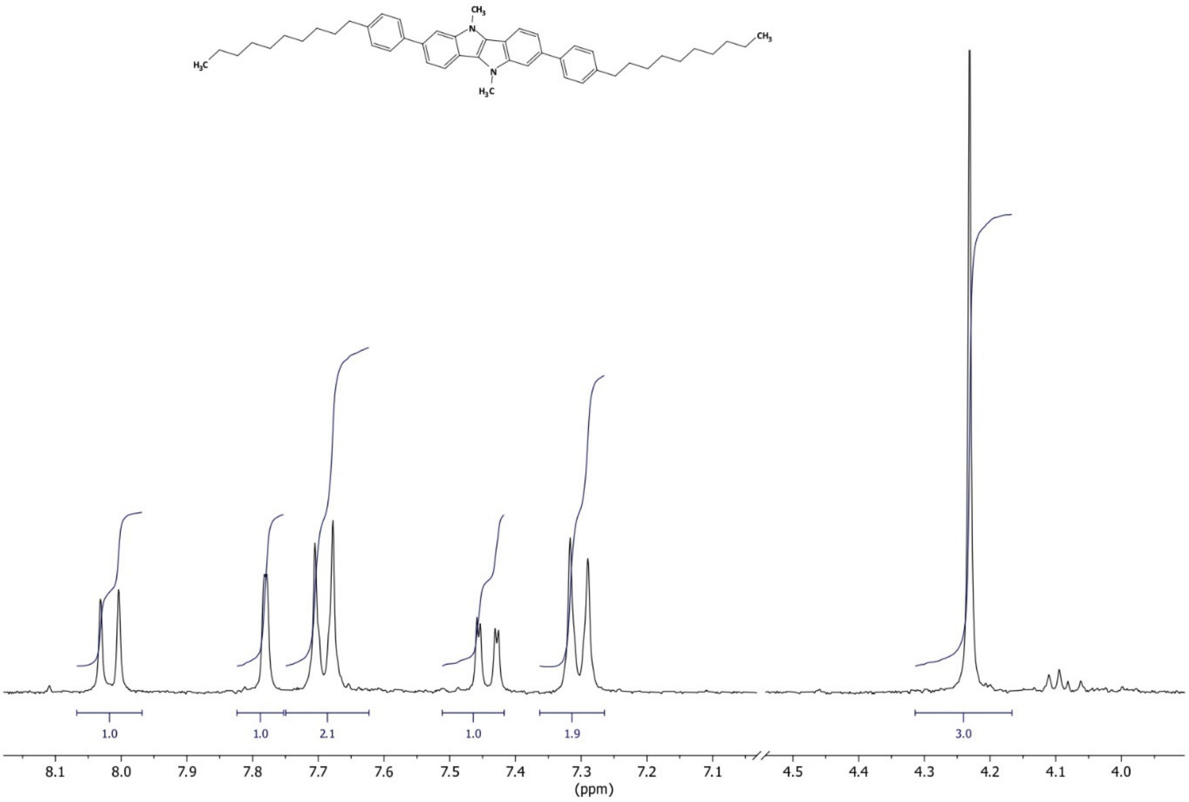

Figure S15. ^{1}H NMR spectrum fragment of compound **ID-PD** in Acetone-d$_6$.

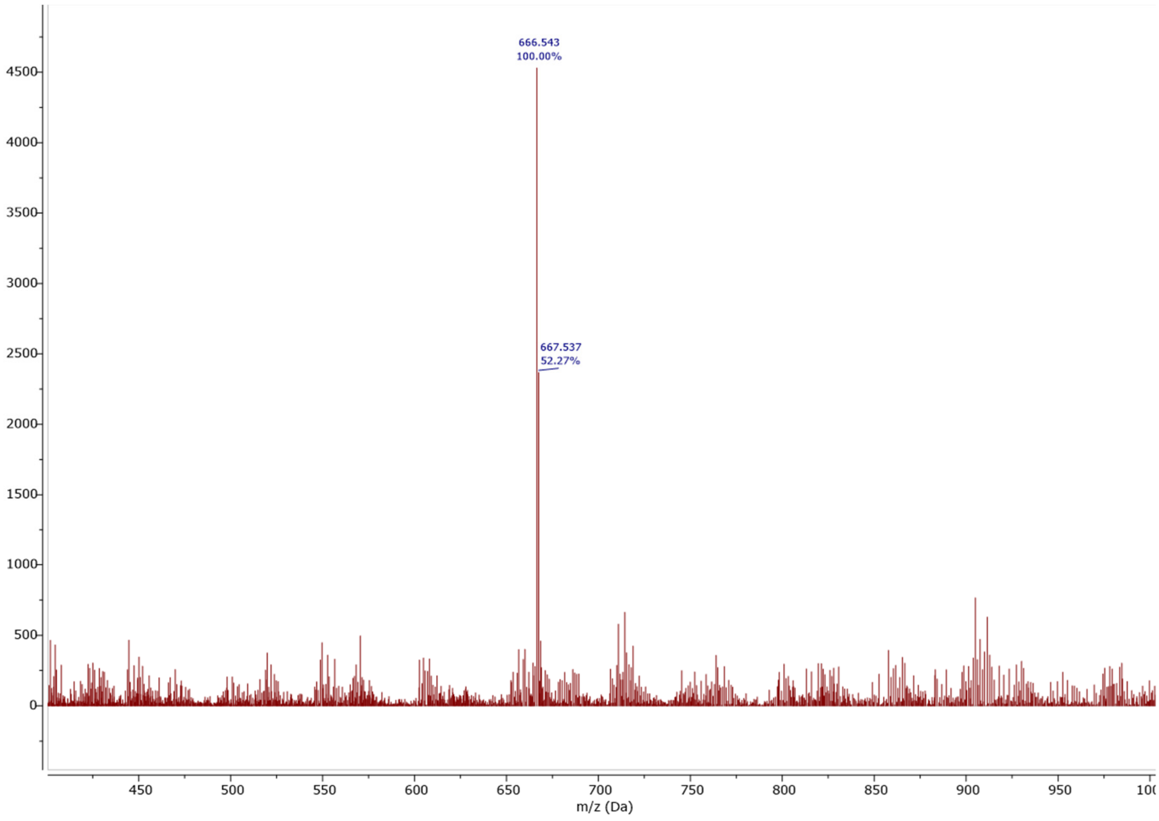

Figure S16. MS spectra for compound **ID-PD**.

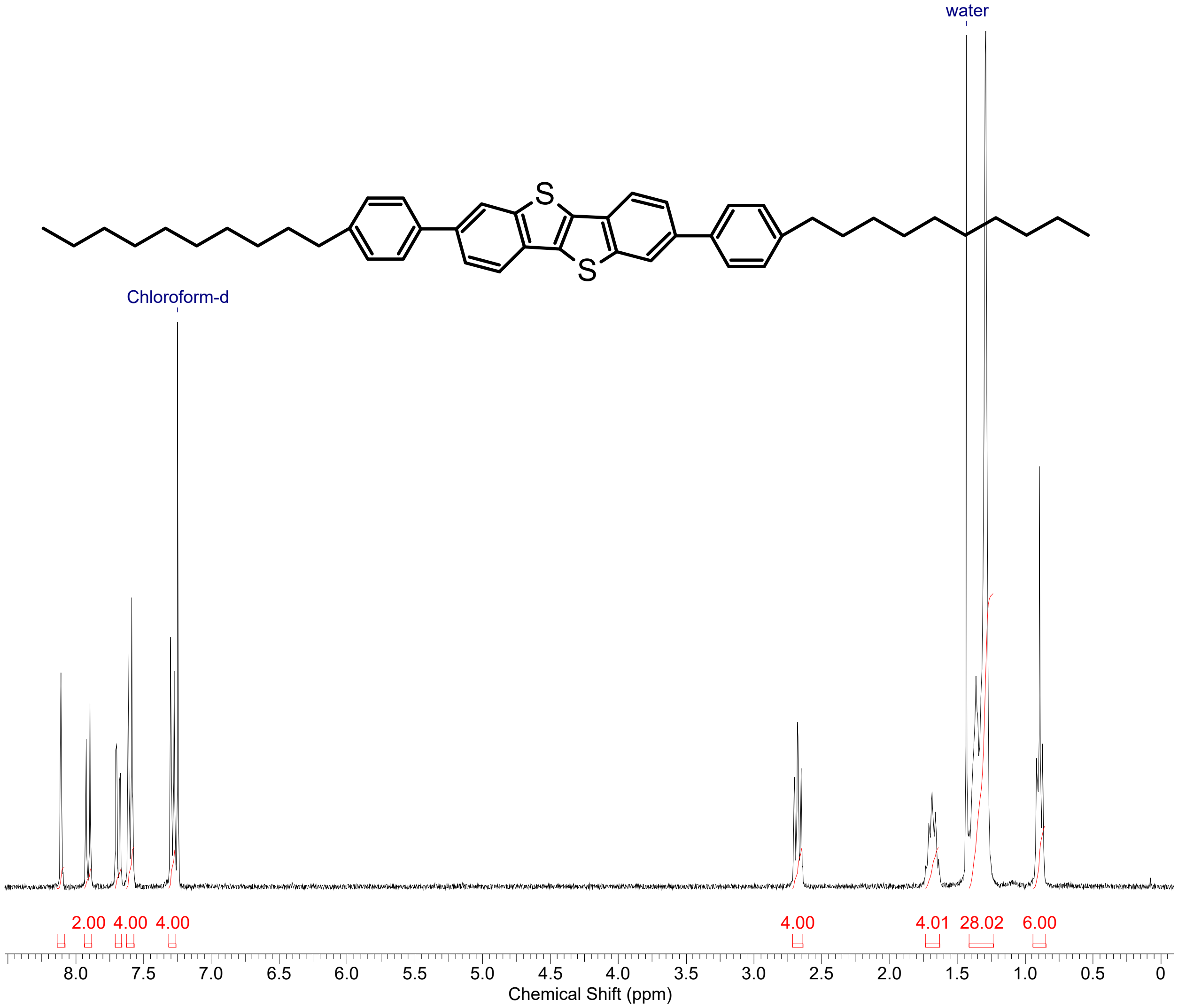

No.	Annotation	(ppm)
1	water	1.43
2	Chloroform-d	7.25

No.	(ppm)	Value	Absolute Value
1	[0.85 .. 0.94]	5.996	6.45024e+9
2	[1.24 .. 1.41]	28.018	3.01424e+10
3	[1.63 .. 1.74]	4.008	4.31236e+9
4	[2.64 .. 2.72]	4.003	4.30609e+9
5	[7.26 .. 7.31]	4.000	4.30327e+9
6	[7.57 .. 7.63]	3.999	4.30189e+9
7	[7.66 .. 7.71]	2.001	2.15243e+9
8	[7.88 .. 7.93]	2.005	2.15663e+9
9	[8.08 .. 8.14]	2.000	2.15164e+9

Figure S17. ^{1}H NMR spectrum of compound **BT-PD** in Chloroform-d.

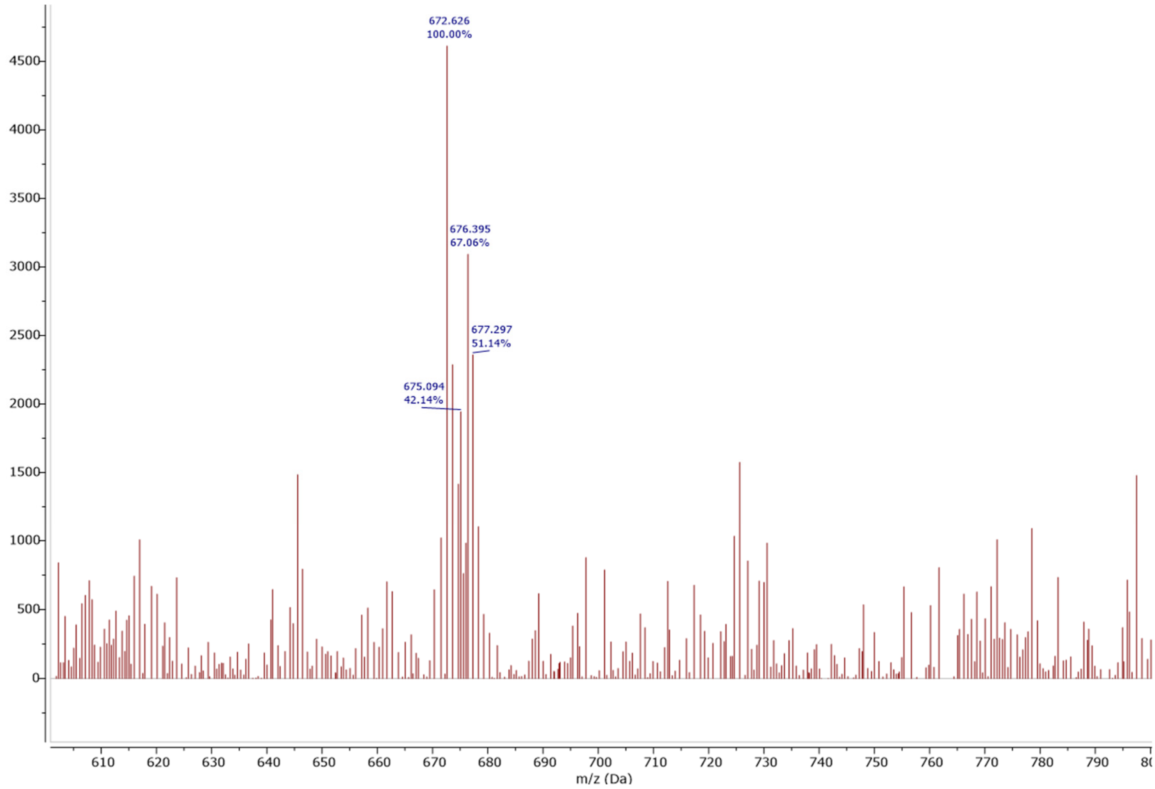

Figure S18. MS spectra for compound **BT-PD**.

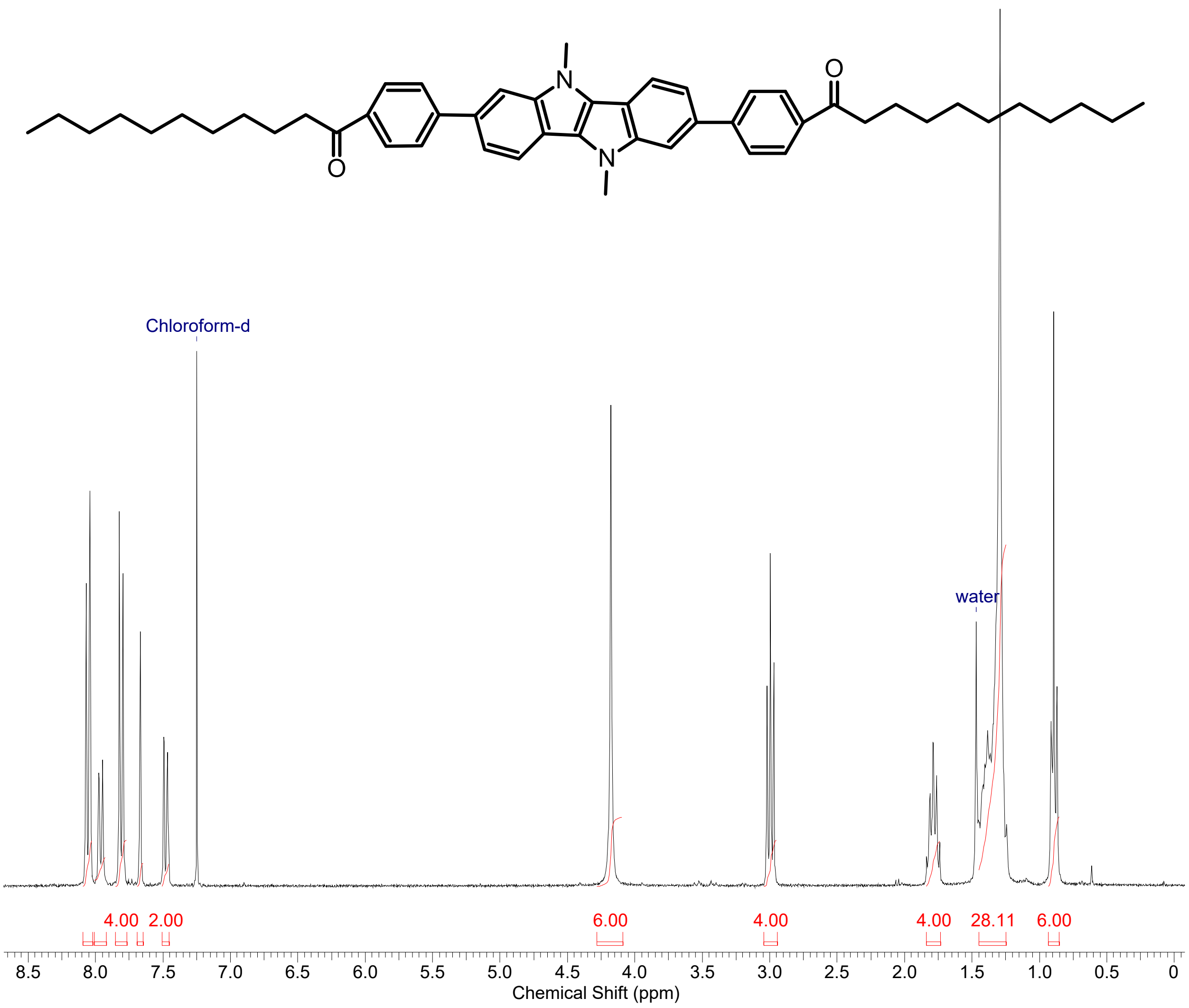

No.	Annotation	(ppm)
1	water	1.47
2	Chloroform-d	7.25

No.	(ppm)	Value	Absolute Value
1	[0.85 .. 0.94]	6.003	6.00809e+9
2	[1.25 .. 1.45]	28.113	2.81349e+10
3	[1.74 .. 1.84]	4.004	4.00735e+9
4	[2.95 .. 3.04]	3.997	3.99979e+9
5	[4.09 .. 4.28]	5.995	6.00016e+9
6	[7.46 .. 7.51]	2.000	2.00127e+9
7	[7.65 .. 7.69]	2.002	2.00330e+9
8	[7.77 .. 7.85]	3.999	4.00254e+9
9	[7.92 .. 8.01]	2.000	2.00153e+9
10	[8.02 .. 8.09]	3.999	4.00189e+9

Figure S19. ^{1}H NMR spectrum of compound **ID-PCOD** in Chloroform-d.

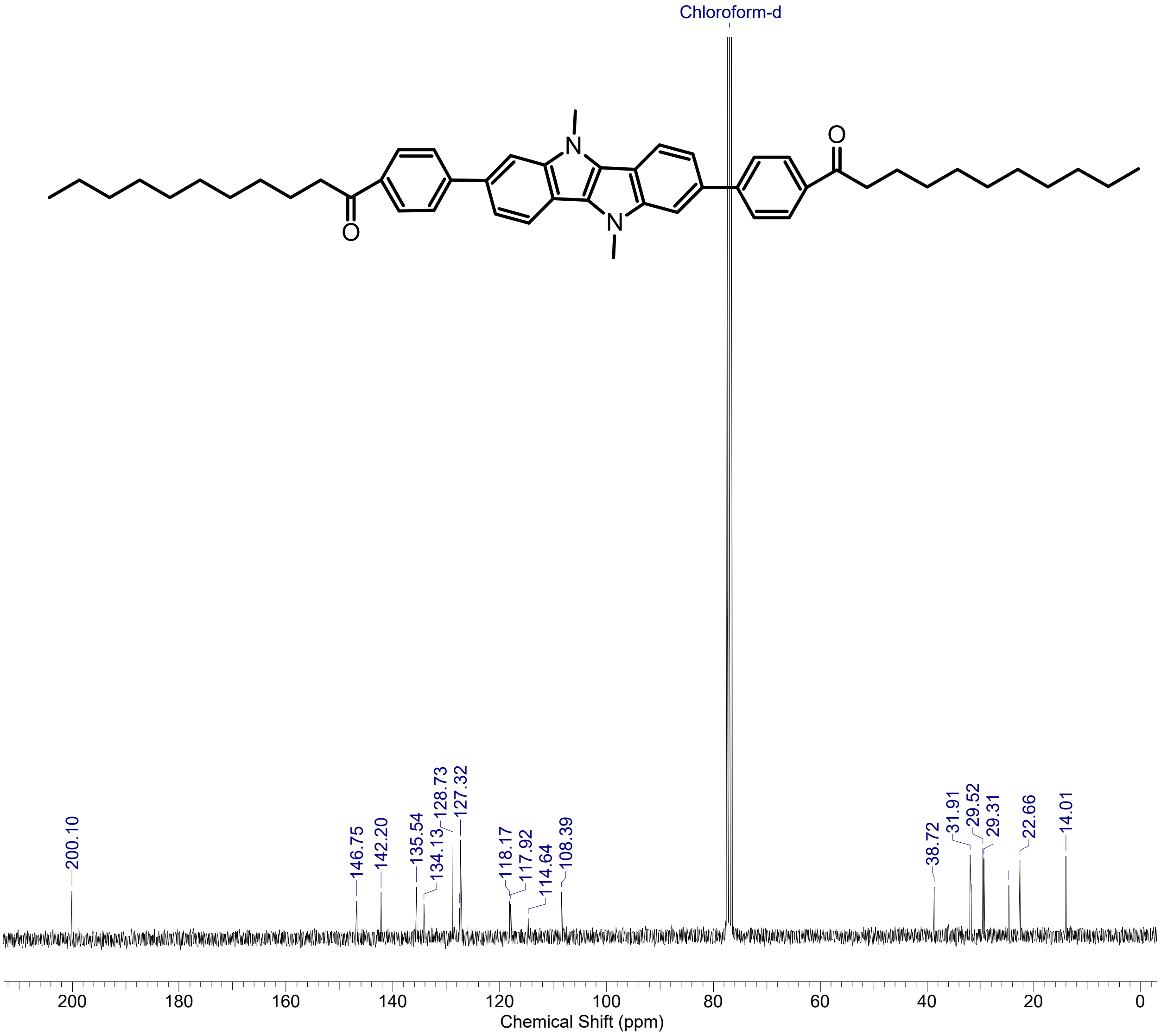

No.	(ppm)	(Hz)	Height
1	14.01	1057.6	0.0700
2	22.66	1710.6	0.0665
3	24.69	1863.2	0.0458
4	29.31	2212.6	0.0678
5	29.50	2227.0	0.0700
6	29.52	2228.1	0.0763
7	29.54	2229.7	0.0760
8	29.60	2234.2	0.0716
9	31.74	2395.6	0.0346
10	31.91	2408.9	0.0709
11	38.72	2922.5	0.0438
12	108.39	8180.8	0.0403

No.	(ppm)	(Hz)	Height
13	114.64	8653.0	0.0180
14	117.92	8900.1	0.0298
15	118.17	8918.9	0.0324
16	127.32	9610.0	0.0829
17	127.55	9627.2	0.0270
18	128.73	9716.2	0.0820
19	134.13	10124.2	0.0304
20	135.54	10230.3	0.0441
21	142.20	10732.9	0.0395
22	146.75	11076.2	0.0327
23	200.10	15103.4	0.0409

No.	Annotation	(ppm)
1	Chloroform-d	77.00

Figure S20. ^{13}C NMR spectrum of compound **ID-PCOD** in Chloroform-d.

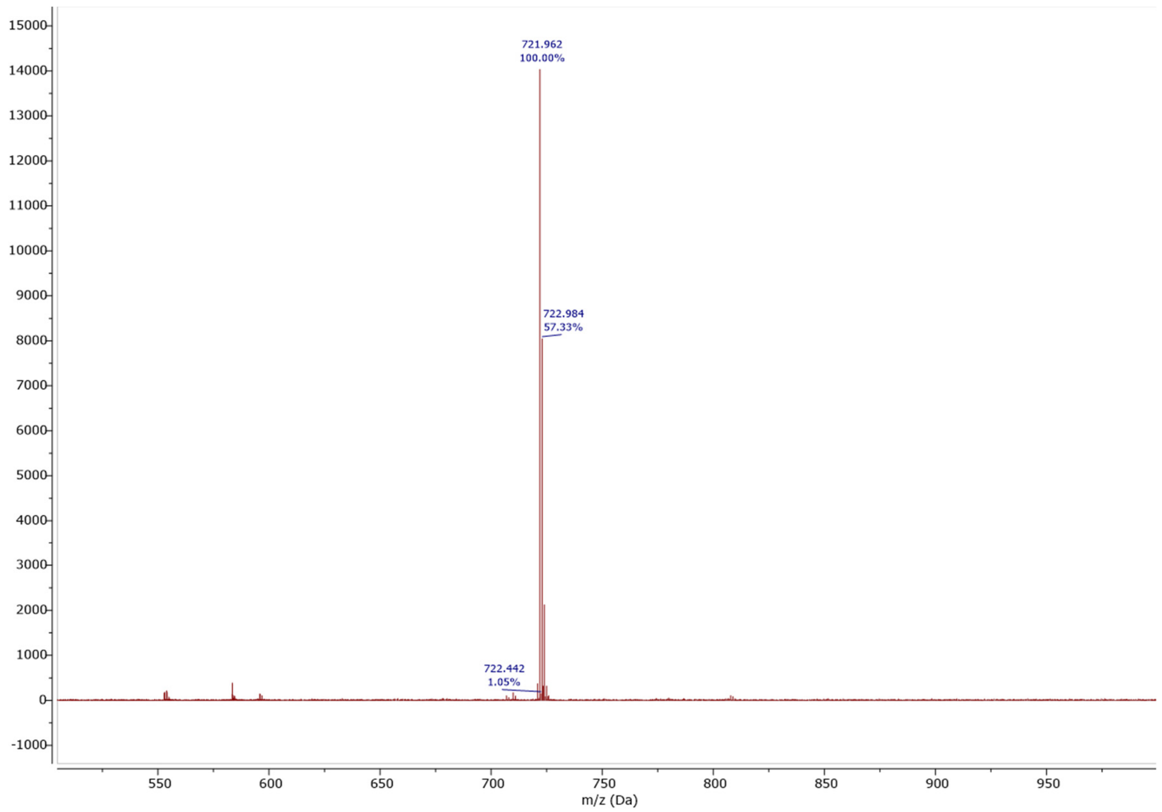

Figure S21. MS spectra for compound **ID-PCOD**.

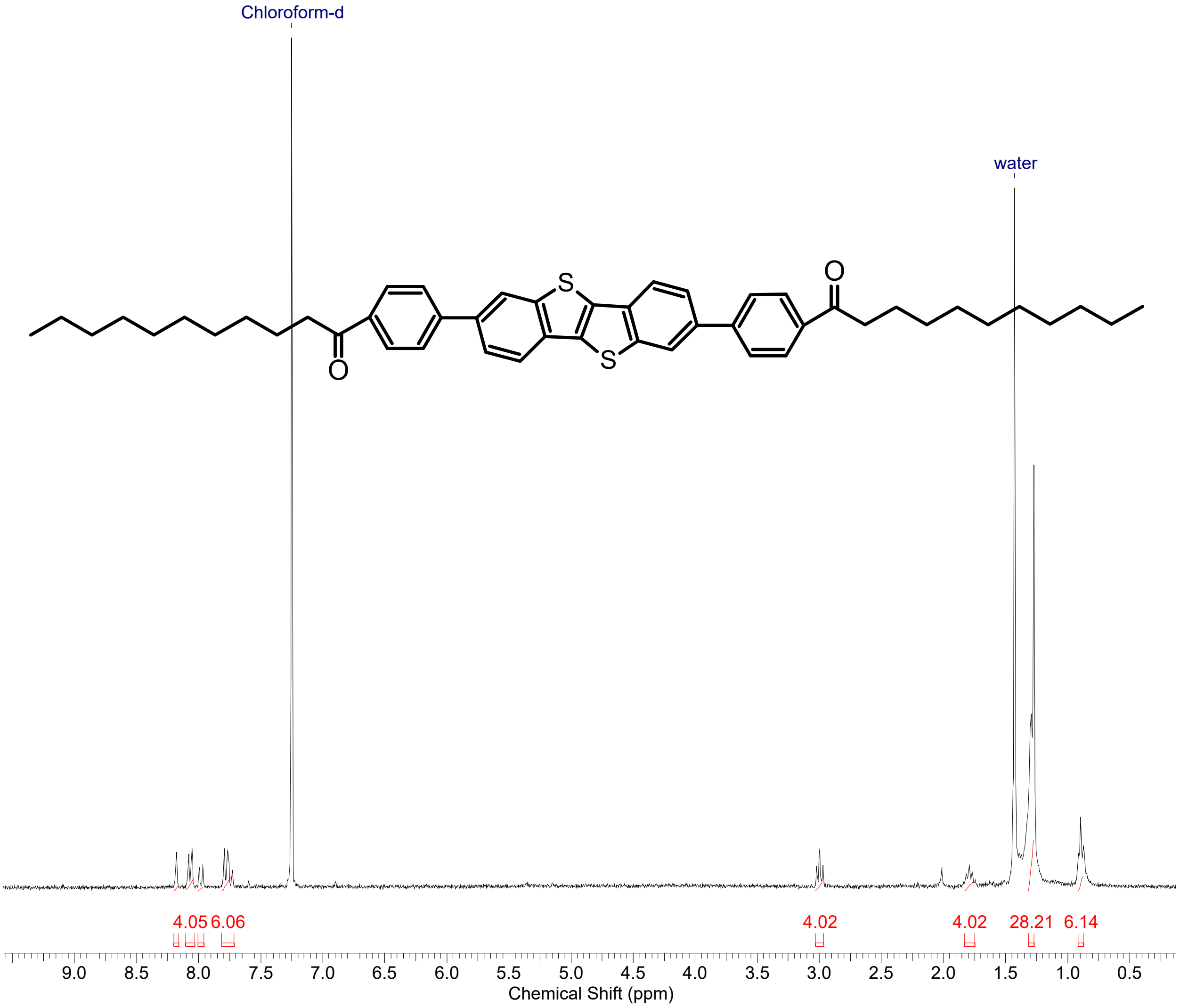

No.	Annotation	(ppm)
1	water	1.43
2	Chloroform-d	7.25

No.	(ppm)	Value	Absolute Value
1	[0.87 .. 0.91]	6.143	1.69659e+9
2	[1.27 .. 1.31]	28.213	7.79189e+9
3	[1.75 .. 1.83]	4.021	1.11055e+9
4	[2.96 .. 3.03]	4.023	1.11111e+9
5	[7.72 .. 7.82]	6.055	1.67232e+9
6	[7.96 .. 8.00]	2.063	5.69677e+8
7	[8.03 .. 8.10]	4.051	1.11883e+9
8	[8.16 .. 8.20]	2.000	5.52370e+8

Figure S22. ^{1}H NMR spectrum of compound **BT-PCOD** in Chloroform-d.

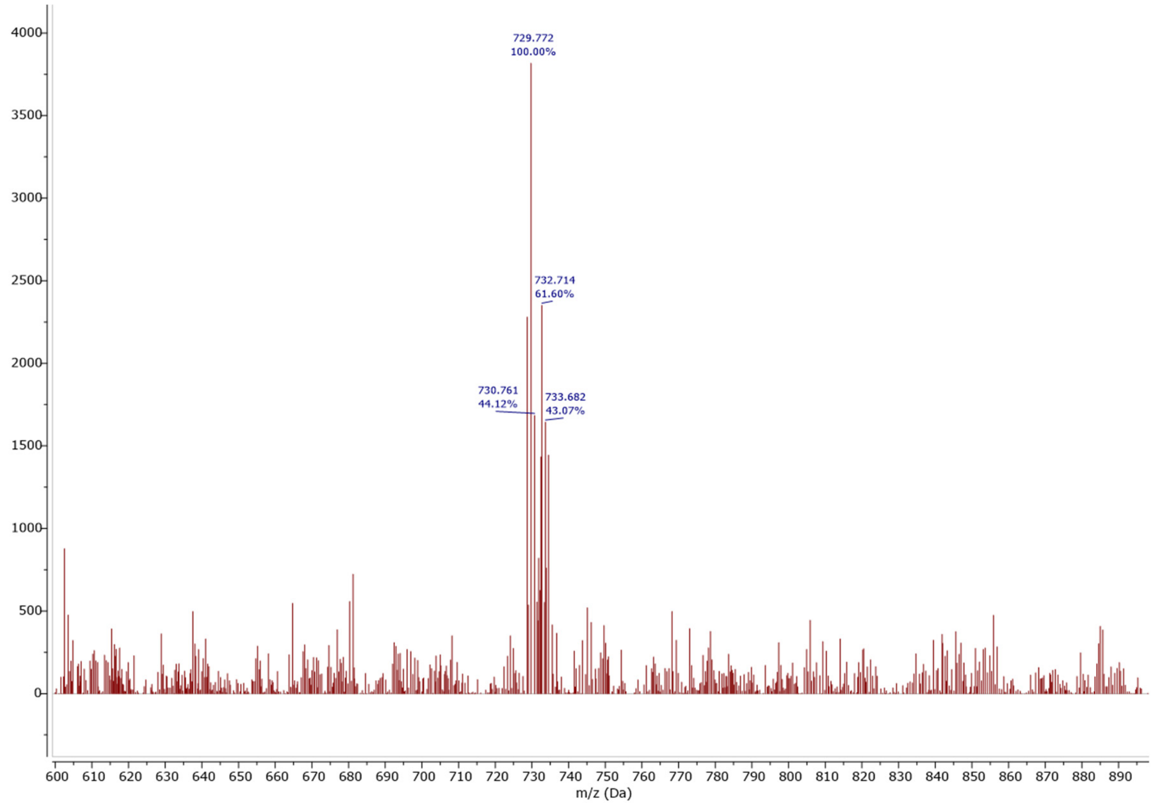

Figure S23. MS spectra for compound **BT-PCOD**.

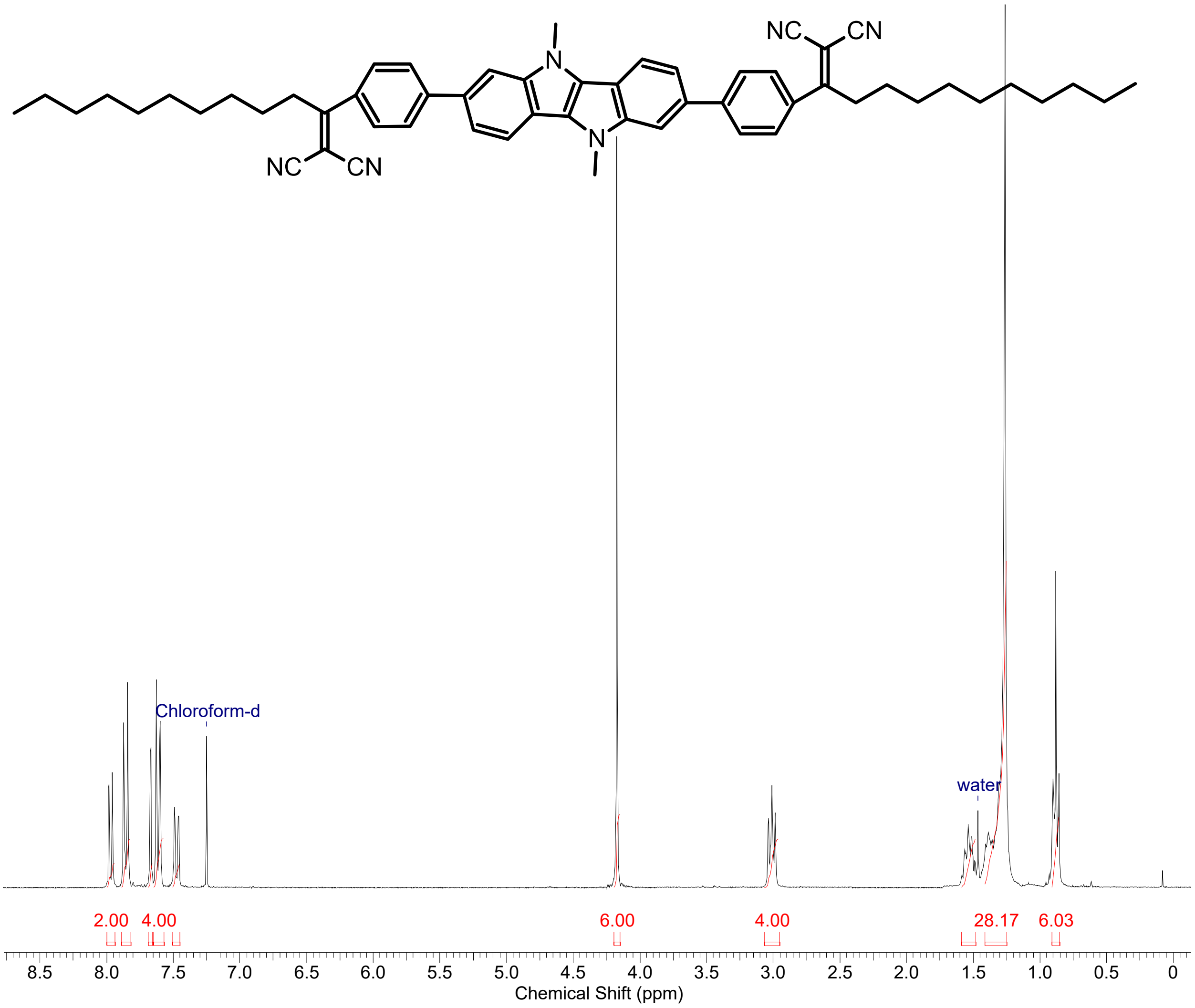

No.	Annotation	(ppm)
1	water	1.46
2	Chloroform-d	7.25

No.	(ppm)	Value	Absolute Value
1	[0.85 .. 0.91]	6.026	2.86154e+9
2	[1.25 .. 1.41]	28.170	1.33765e+10
3	[1.48 .. 1.59]	4.007	1.90296e+9
4	[2.95 .. 3.07]	3.999	1.89872e+9
5	[4.15 .. 4.20]	6.001	2.84966e+9
6	[7.45 .. 7.50]	1.996	9.47720e+8
7	[7.57 .. 7.65]	4.002	1.90053e+9
8	[7.65 .. 7.69]	2.000	9.49718e+8
9	[7.82 .. 7.89]	4.000	1.89956e+9
10	[7.94 .. 8.00]	2.002	9.50644e+8

Figure S24. ^{1}H NMR spectrum of compound **ID-PDD** in Chloroform-d.

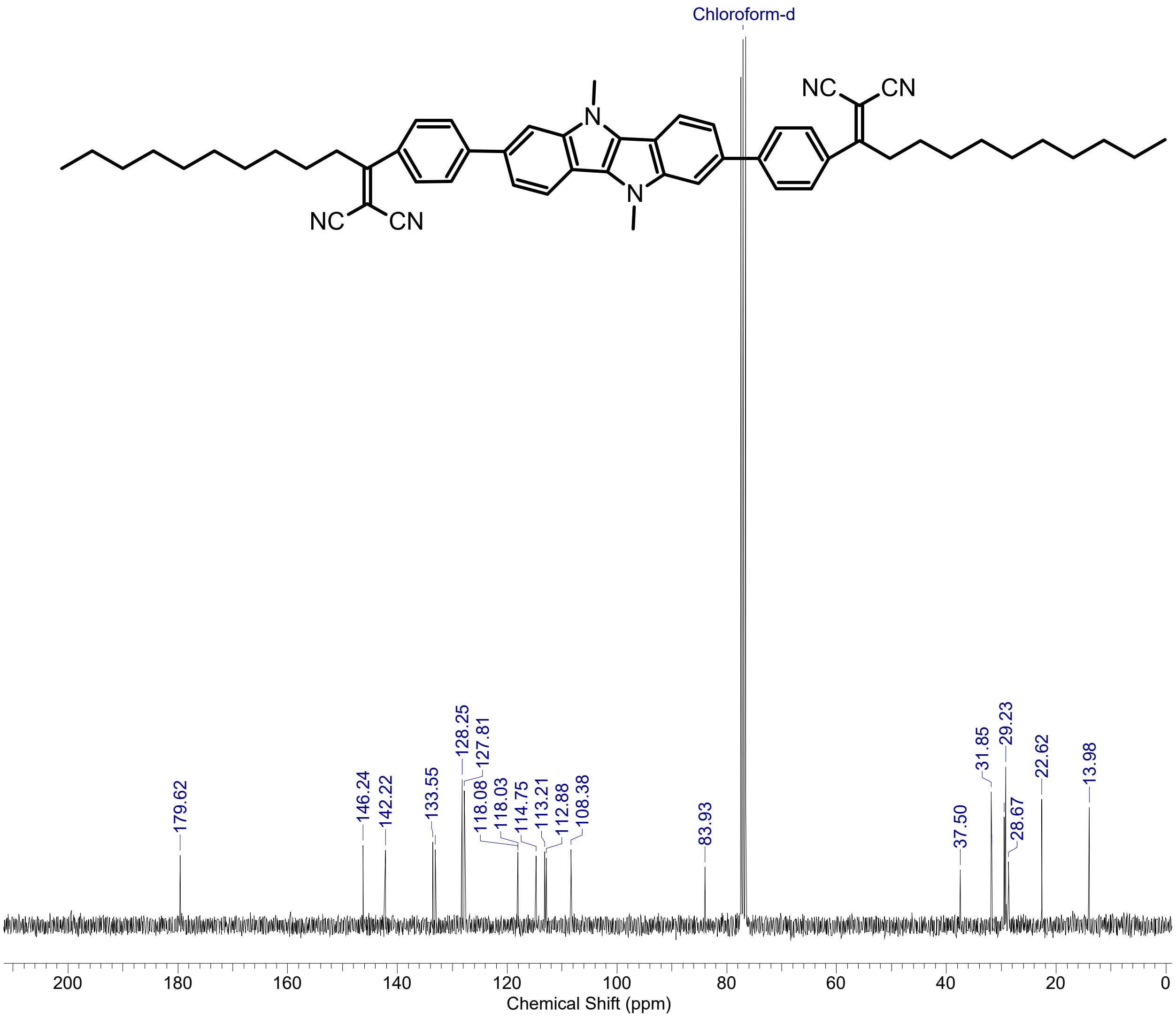

No.	(ppm)	(Hz)	Height
1	13.98	1055.4	0.1330
2	22.62	1707.3	0.1419
3	28.67	2163.9	0.0716
4	29.09	2196.0	0.1071
5	29.23	2206.0	0.1789
6	29.34	2214.3	0.1138
7	29.45	2223.1	0.1220
8	31.73	2395.0	0.0860
9	31.85	2403.9	0.1502
10	37.50	2830.2	0.0627
11	83.93	6334.8	0.0657
12	108.38	8180.3	0.0858
13	112.88	8520.3	0.0757

No.	(ppm)	(Hz)	Height
14	113.21	8544.6	0.0833
15	114.75	8661.3	0.0783
16	118.03	8908.4	0.0823
17	118.08	8912.3	0.0789
18	127.66	9635.5	0.0644
19	127.81	9647.1	0.1518
20	128.25	9679.7	0.1645
21	133.08	10044.6	0.0855
22	133.55	10080.0	0.0941
23	142.22	10734.6	0.0849
24	146.24	11037.5	0.0902
25	179.62	13557.0	0.0792

No.	Annotation	(ppm)
1	Chloroform-d	77.00

Figure S25. ^{13}C NMR spectrum of compound **ID-PDD** in Chloroform-d.

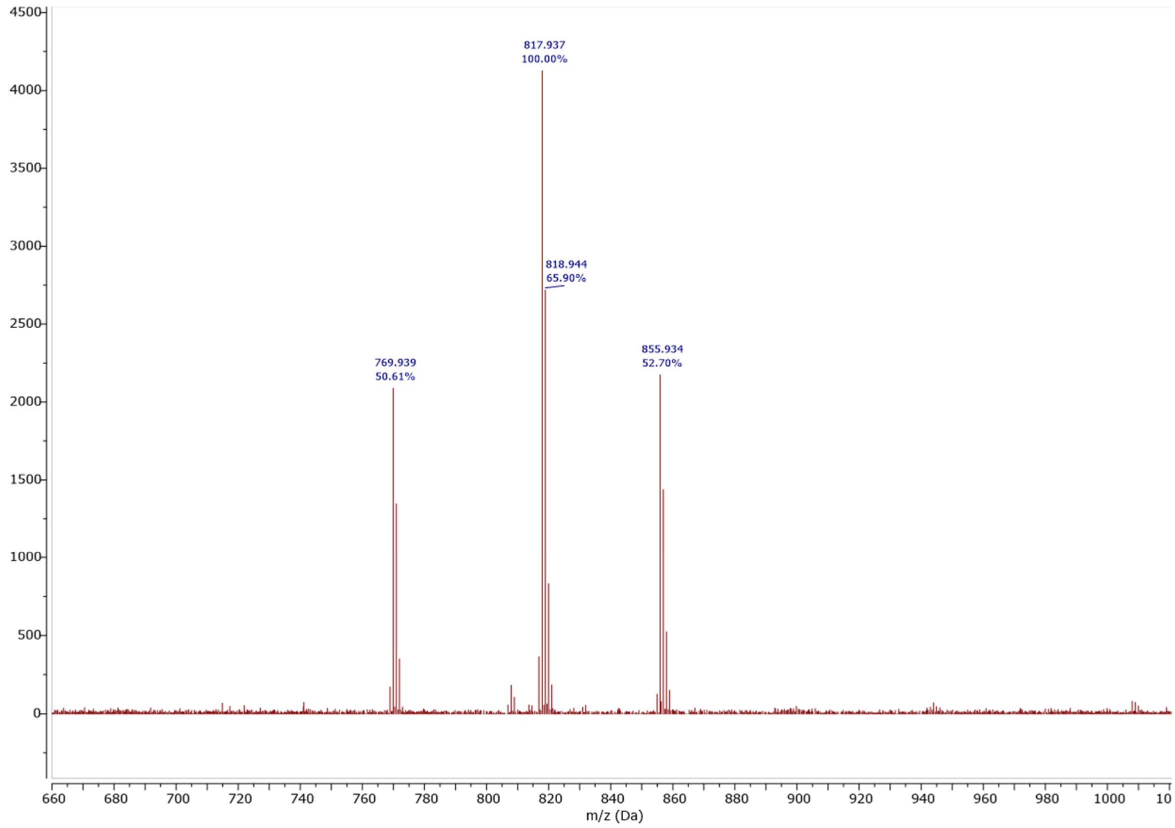

Figure S26. MS spectra for compound **ID-PDD**.

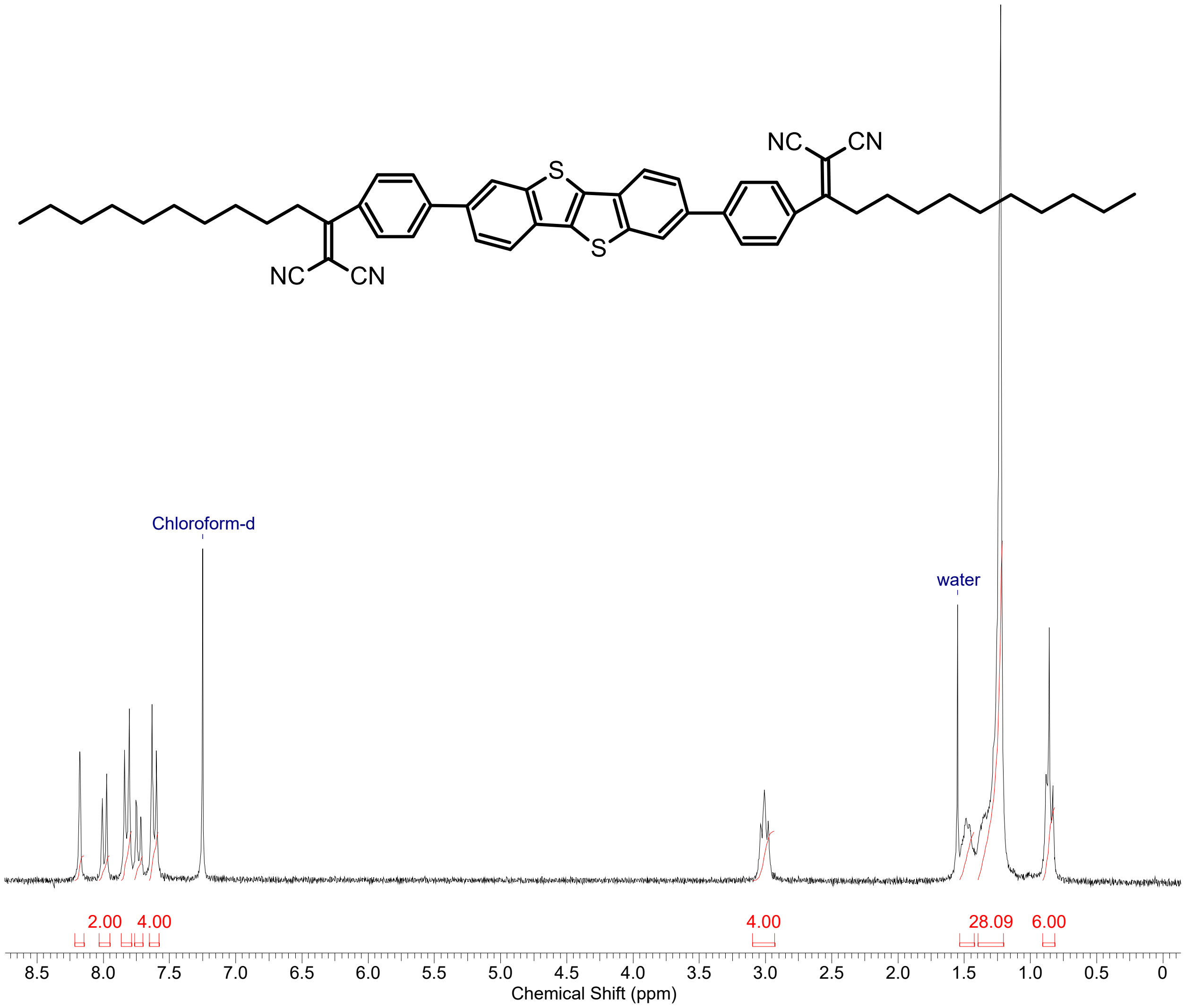

No.	Annotation	(ppm)
1	water	1.55
2	Chloroform-d	7.25

No.	(ppm)	Value	Absolute Value
1	[0.82 .. 0.91]	6.001	1.76323e+7
2	[1.21 .. 1.40]	28.093	8.25500e+7
3	[1.43 .. 1.53]	3.997	1.17465e+7
4	[2.93 .. 3.10]	3.997	1.17457e+7
5	[7.58 .. 7.65]	4.003	1.17623e+7
6	[7.70 .. 7.76]	2.000	5.87592e+6
7	[7.79 .. 7.86]	3.996	1.17409e+7
8	[7.95 .. 8.03]	2.005	5.89090e+6
9	[8.15 .. 8.22]	2.000	5.87692e+6

Figure S27. ^{1}H NMR spectrum of compound **BT-PDD** in Chloroform-d.

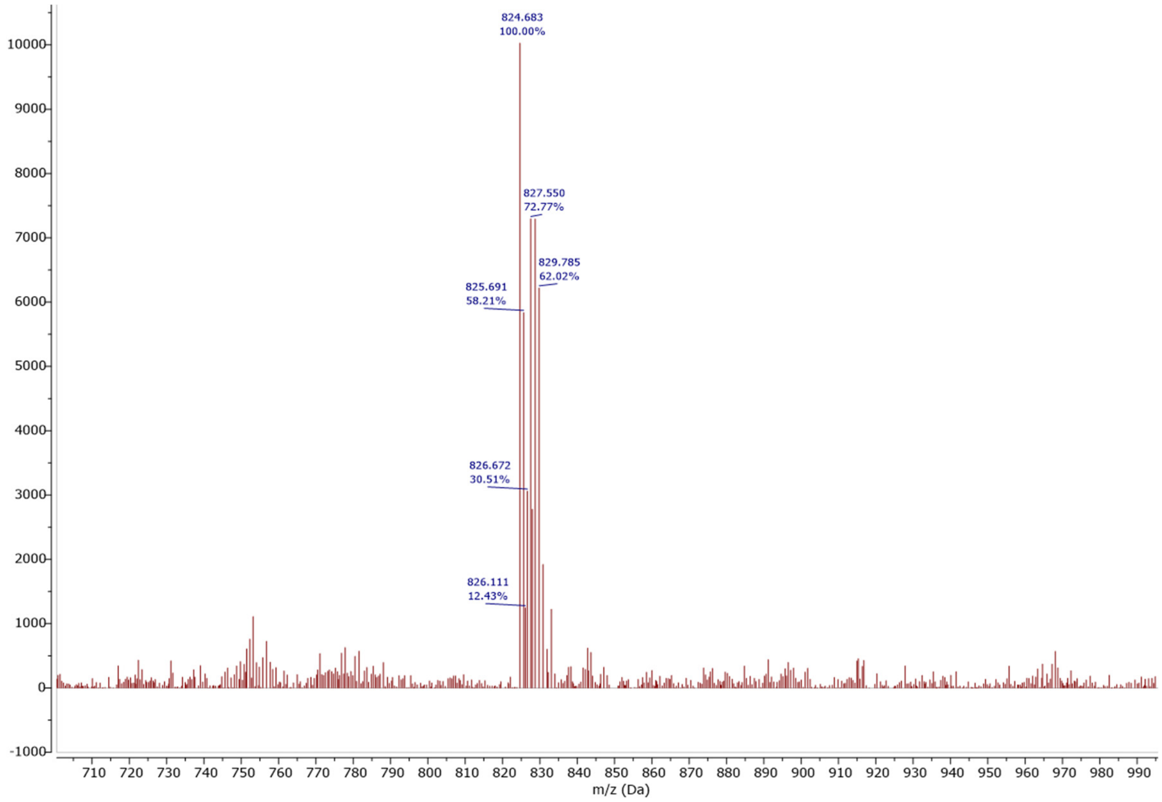

Figure S28. MS spectra for compound **BT-PDD**.

2. Optical Data

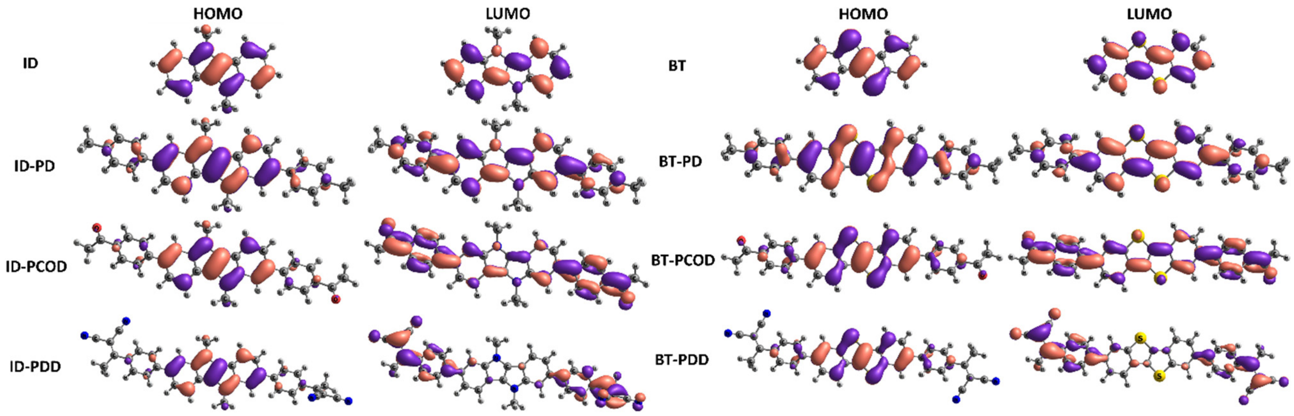

Figure S29. HOMO and LUMO of model and target compounds.

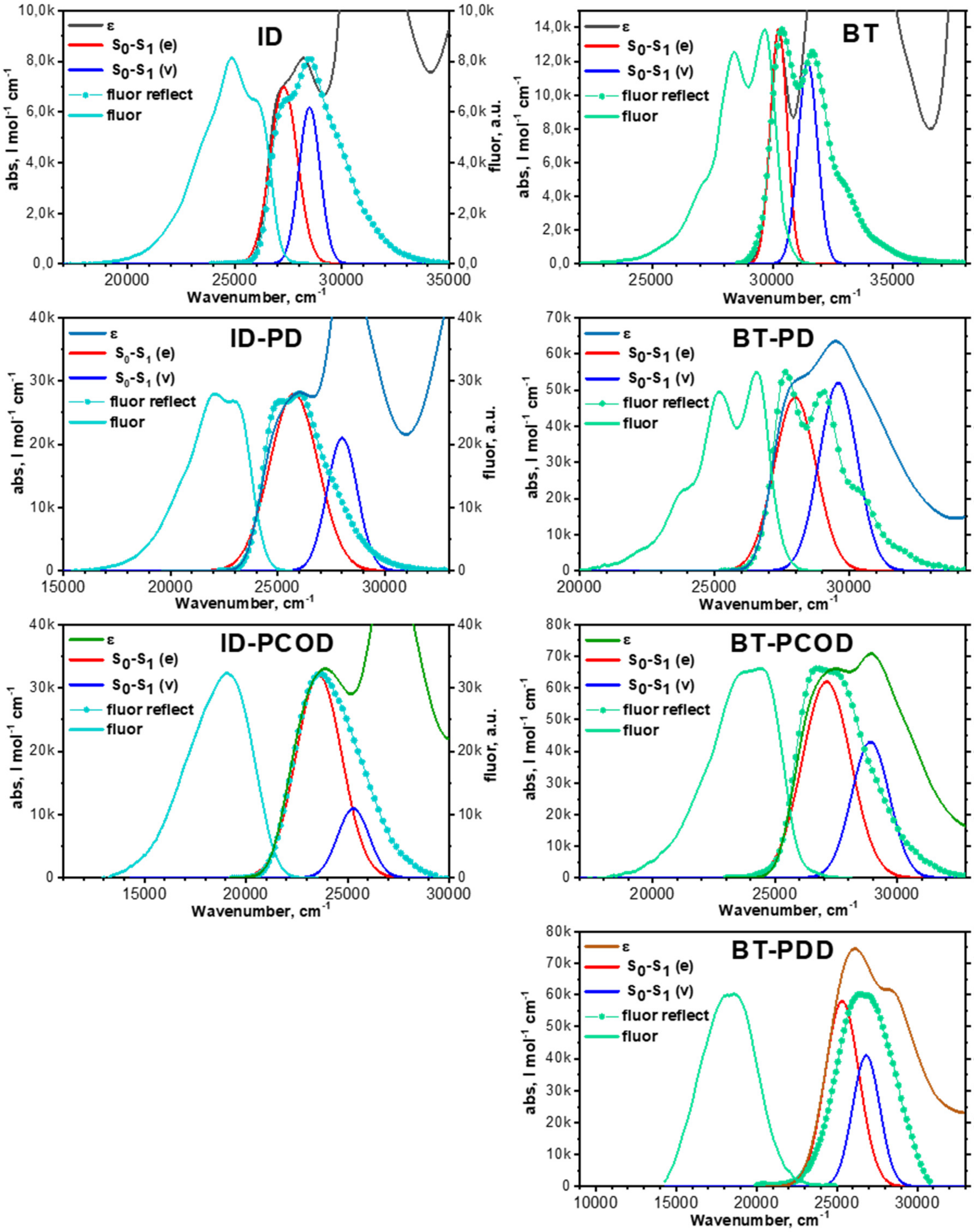

Figure S30. Mirror symmetry in the absorption and emission spectra of condensed compound solutions based on ID and BT cores in THF.

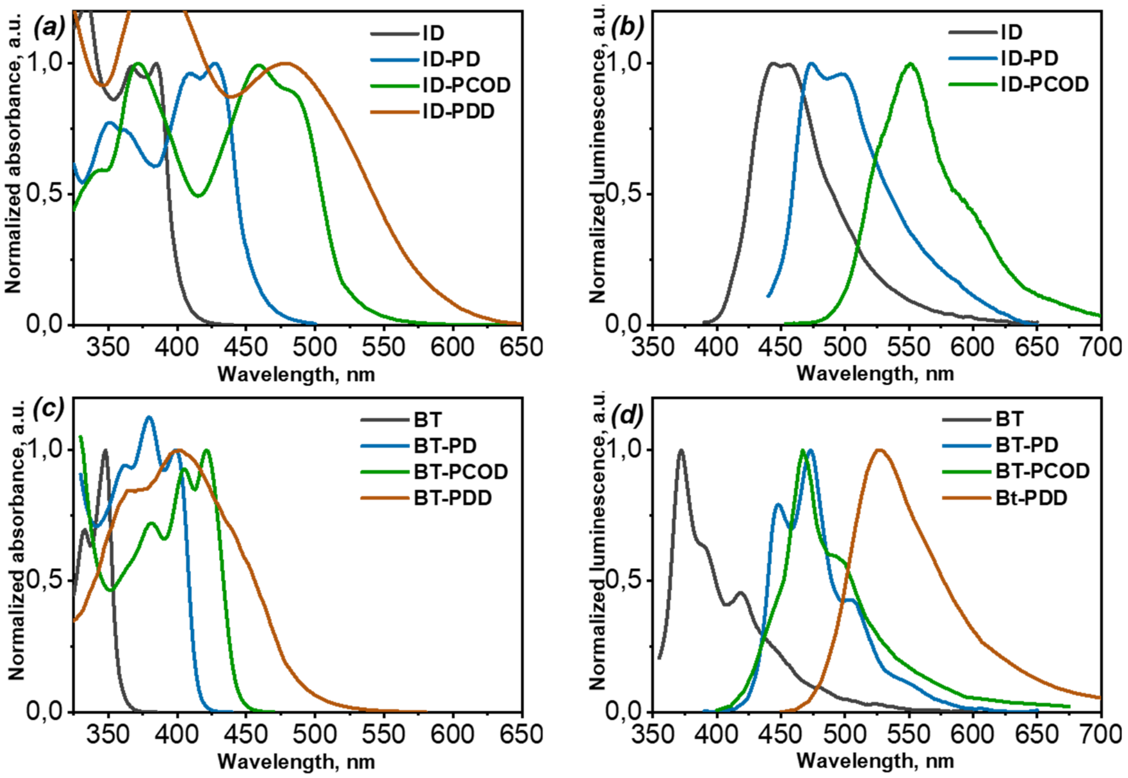

Figure S31. Normalized absorption spectra of thin polycrystalline films of condensed compounds with ID (a) and BT (c) cores. Normalized fluorescence spectra of condensed compound powders with ID (b) and BT (d) cores.

3. CV Data

Table S1. Electrochemical properties and theoretical DFT data of the model and target compounds.

**Compound**	**HOMO, eV**				**LUMO, eV**				**E_g, eV**			
	THF solution		film	GAS	THF solution		film	GAS	THF solution		film	GAS
	CV	DFT	CV	DFT	CV	DFT	CV	DFT	CV	DFT	CV	DFT
**ID**	-5.00	-5.26	-5.04	-5.18	-1.77	-0.98	-1.94	-0.87	3.23	4.28	3.10	4.31
**ID-PD**	-5.10	-5.17	-5.10	-5.09	-2.18	-1.37	-2.37	-1.24	2.92	3.80	2.73	3.85
**ID-PCOD**	-5.25	-5.34	-5.26	-5.44	-2.60	-2.06	-2.57	-2.01	2.65	3.28	2.69	3.43
**ID-PDD**	-5.26	-5.40	-5.26	-5.70	-3.25	-2.70	-3.22	-2.84	2.01	2.70	2.04	2.86
**BT**	-5.85	-6.05	-5.88	-6.00	-2.19	-1.48	-2.41	-1.45	3.66	4.57	3.47	4.55
**BT-PD**	-5.89	-5.80	-5.92	-5.73	-2.61	-1.74	-2.94	-1.65	3.28	4.06	2.98	4.08
**BT-PCOD**	-6.00	-6.01	-6.08	-6.10	-2.46	-2.25	-2.42	-2.27	3.54	3.76	3.66	3.83
**BT-PDD**	-6.00	-6.05	-6.08	-6.34	-3.10	-2.75	-3.07	-2.98	2.90	3.30	3.01	3.36

Notes: HOMO is energy of the highest occupied molecular orbital; LUMO is energy of the lowest unoccupied molecular orbital; E_g is band gap; DFT/PBE0/def2-TZVp/PCM; the values highlighted in blue – found as $E_{LUMO}^{opt} = E_g^{opt} - E_{HOMO}^{CV}$; the values highlighted in green represent the optical band gap (E_g^{opt}, eV), calculated as $E_g^{opt} = 1240/\lambda_{edge}$

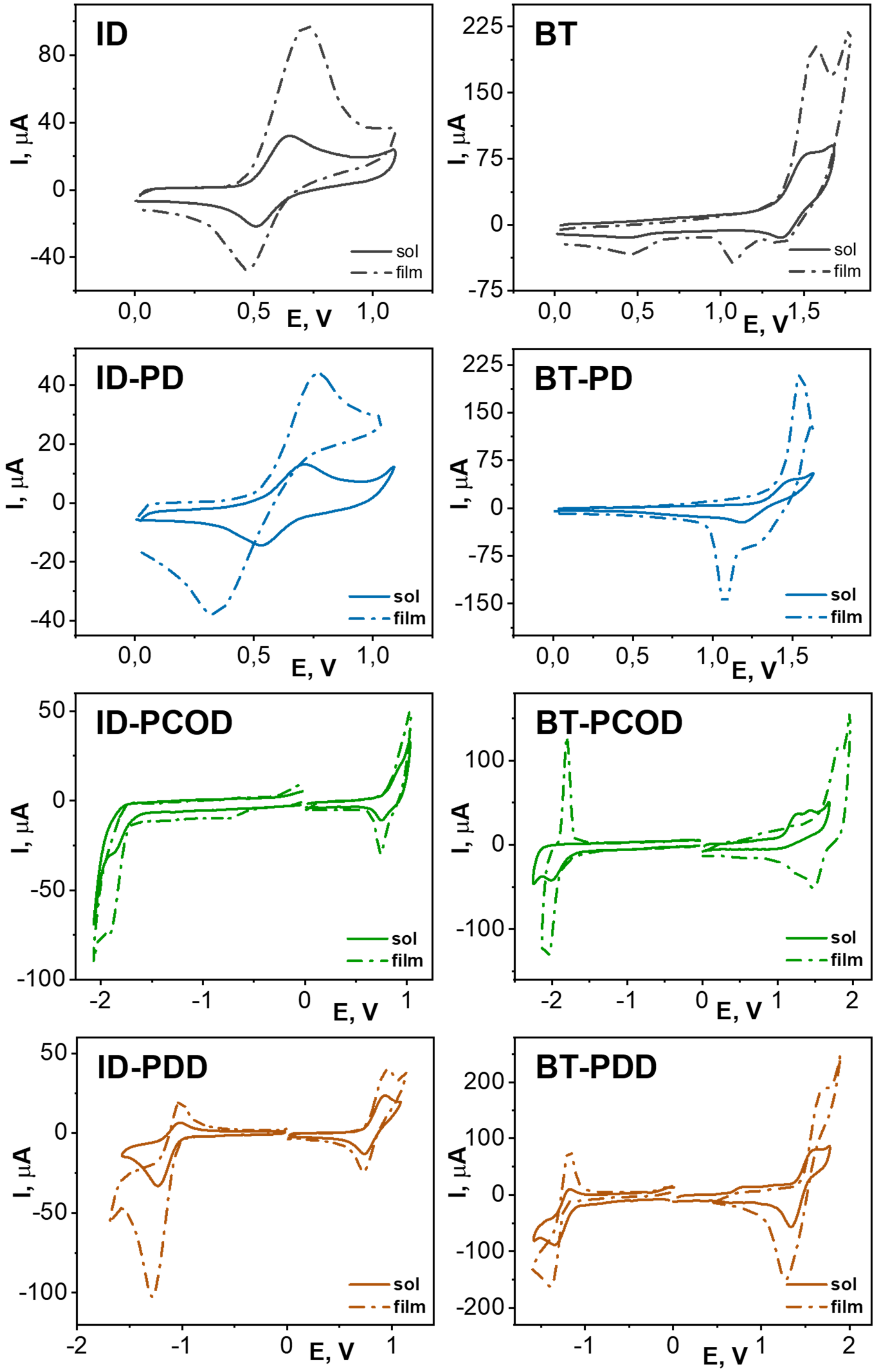

Figure S32. Cyclic voltammograms of the model and target compounds.

4. TGA and DSC Data

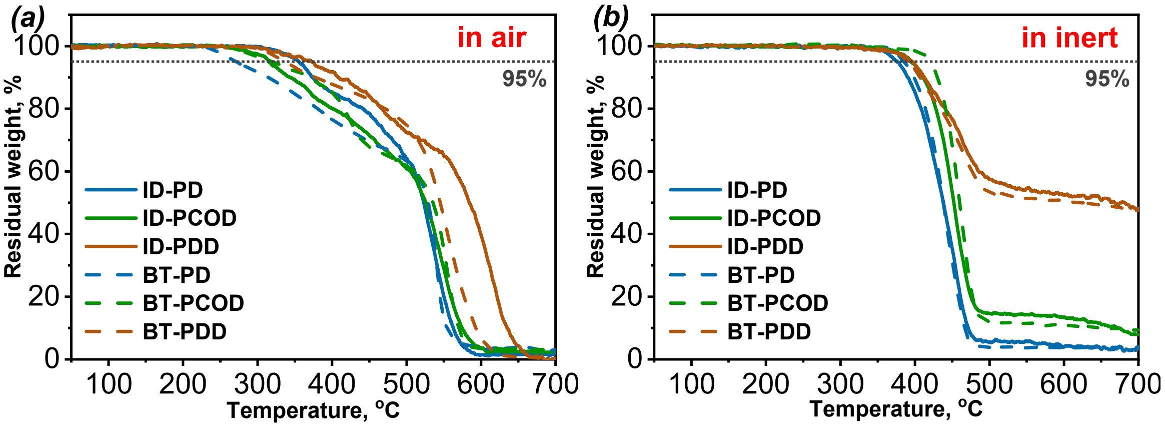

Figure S33. TGA curves in air (a) and under (b) inert atmosphere for the obtained compounds.

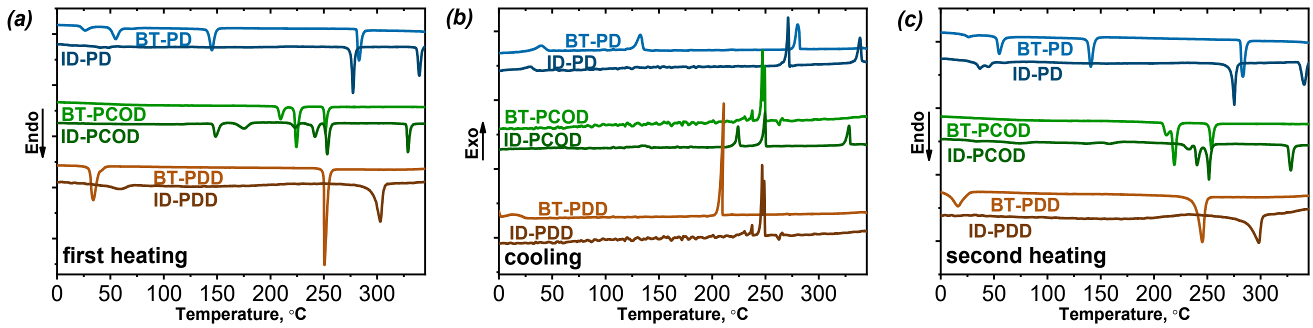

Figure S34. DSC curves for the first heating (a), cooling (b) and second heating (c).

Table S2. Phase transitions found on the DSC curves of target compounds.

Compound	T_{trans}, °C (ΔH_{trans}, J/g)		
	**1-st heating transitions**	**cooling transitions**	**2-nd heating transitions**
**ID-PD**	40.6 (-6.3); 276.9 (-27.7); 339.5 (-18.9)	338.9 (19.1); 271.7 (26.6); 29.5 (24.9)	36.7 (-24.3); 275.1 (-27.6); 340.5 (-18.2)
**ID-PCOD**	148.5 (-12.9); 175.5 (-11.1); 223.2 (-4.8); 241.7 (-12.6); 253.2 (-19.8); 328.8 (-16.1)	328.3 (13.6); 249.6 (20.0); 224.3 (13.8); 134.79 (9.4); 61.8 (10.5)	33.7 (-0.8); 73.5 (-4.0); 158.0 (-5.3); 251.4 (-45.9); 328.2 (-15.3)
**ID-PDD**	58.8 (-18.9); 302.9 (-66.9)	247.2 (49.3)	1.2 (-8.9); 298.1 (-64.8)
**BT-PD**	26.3 (-6.5); 54.9 (-17.1); 145.1 (-21.9); 283.0 (-21.3)	280.1 (21.2); 132.8 (22.1); 39.2 (17.2)	26.01 (-3.4); 54.9 (-13.8); 140.7 (-21.3); 283.4 (-21.9)
**BT-PCOD**	209.6 (-12.3); 224.3 (-29.5); 251.4 (-14.4)	252.2 (13.8); 213.1 (37.1)	219.1 (-37.3); 253.8 (-14.9)
**BT-PDD**	34.0 (-44.2); 250.6 (-58.7)	210.8 (47.9); -1.5 (32.9)	16.1 (-33.2); 245.2 (-47.8)

Notes: T_{trans} is temperature of endothermic phase transitions according; ΔH_{trans} is enthalpies of endothermic phase transitions according.

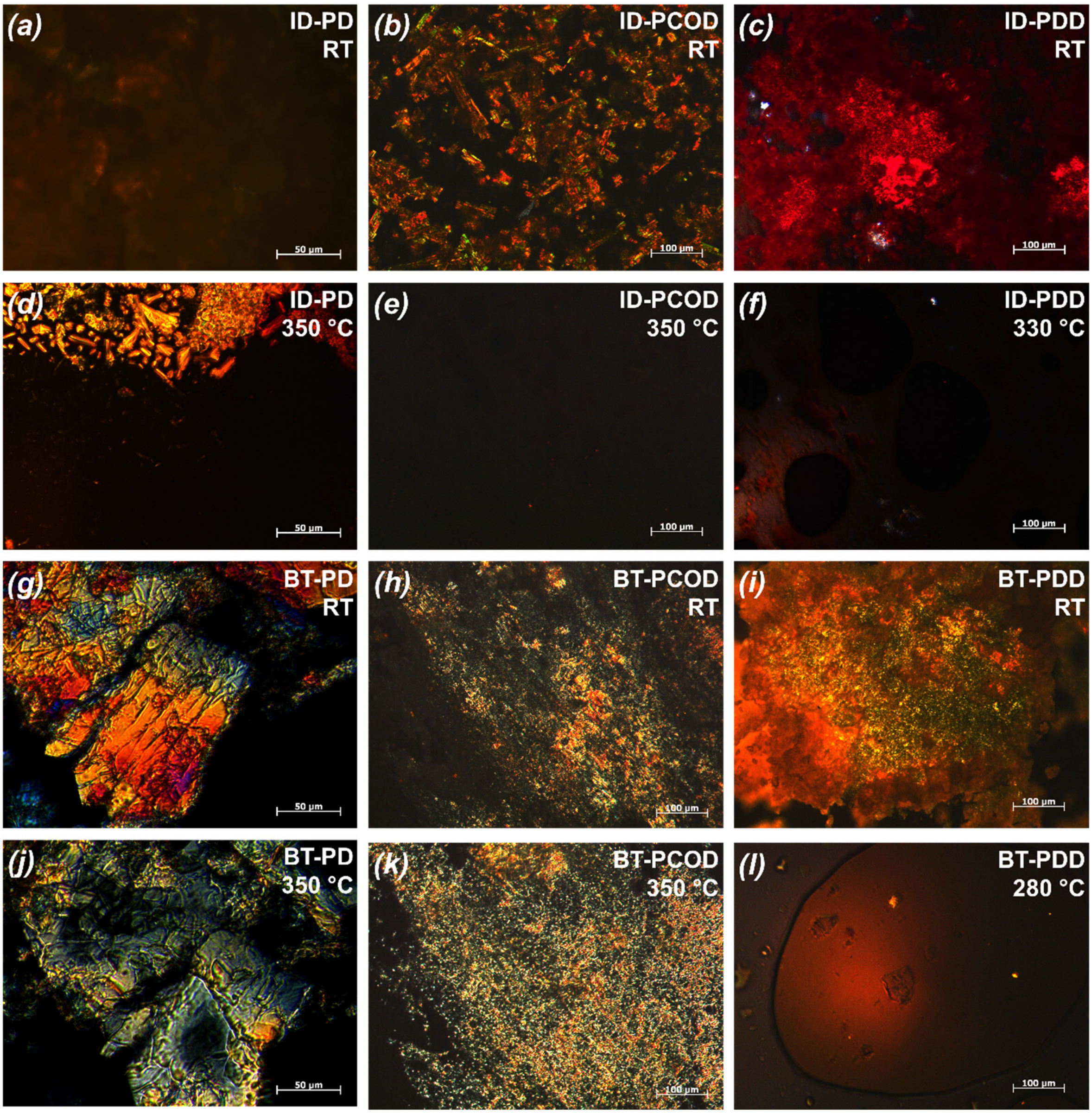

Figure S35. Optical polarizing microscopy of the target compound.

5. X-ray Data

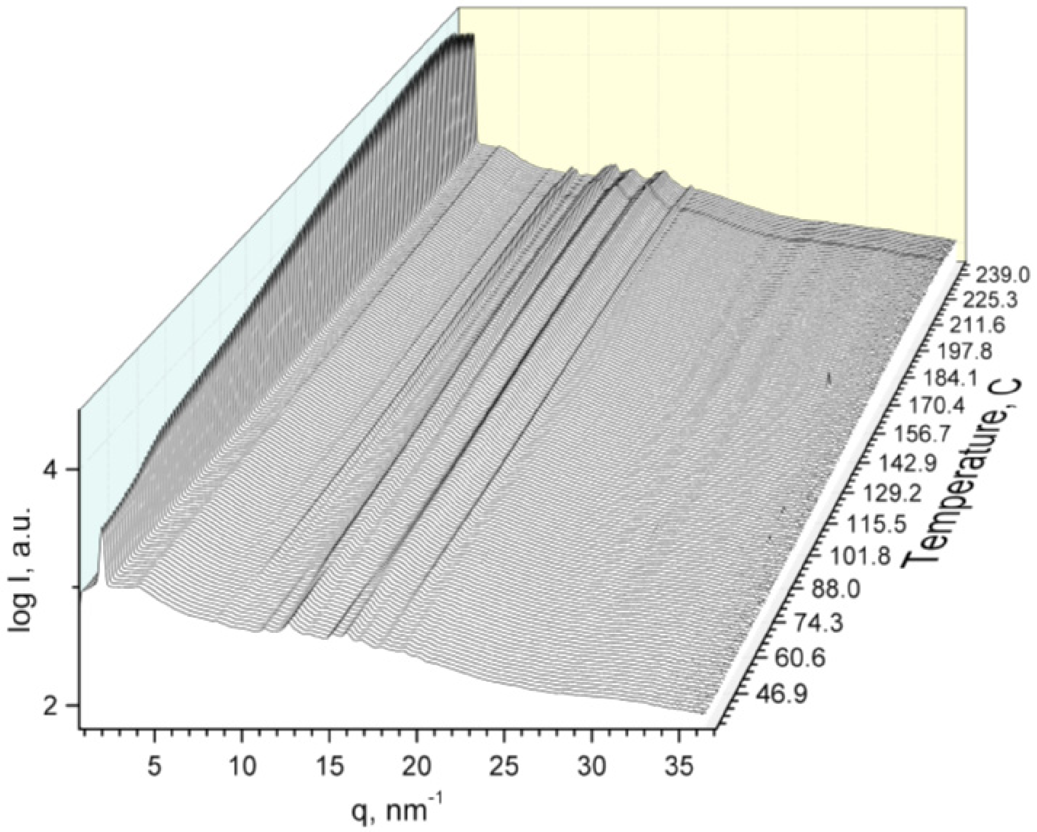

Figure S36. TWAXS plot of **ID-PDD** at heating rate of 7 C/min

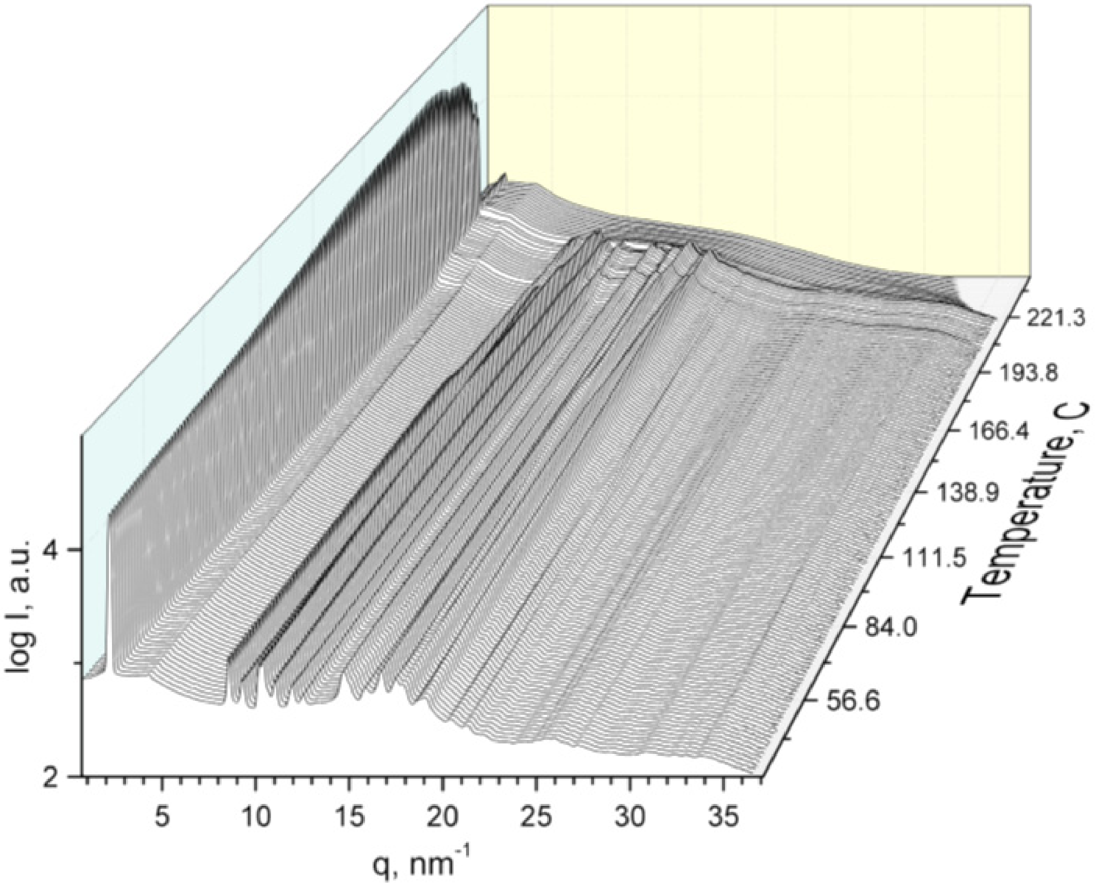

Figure S37. TWAXS plot of **BT-PDD** at heating rate of 7 C/min

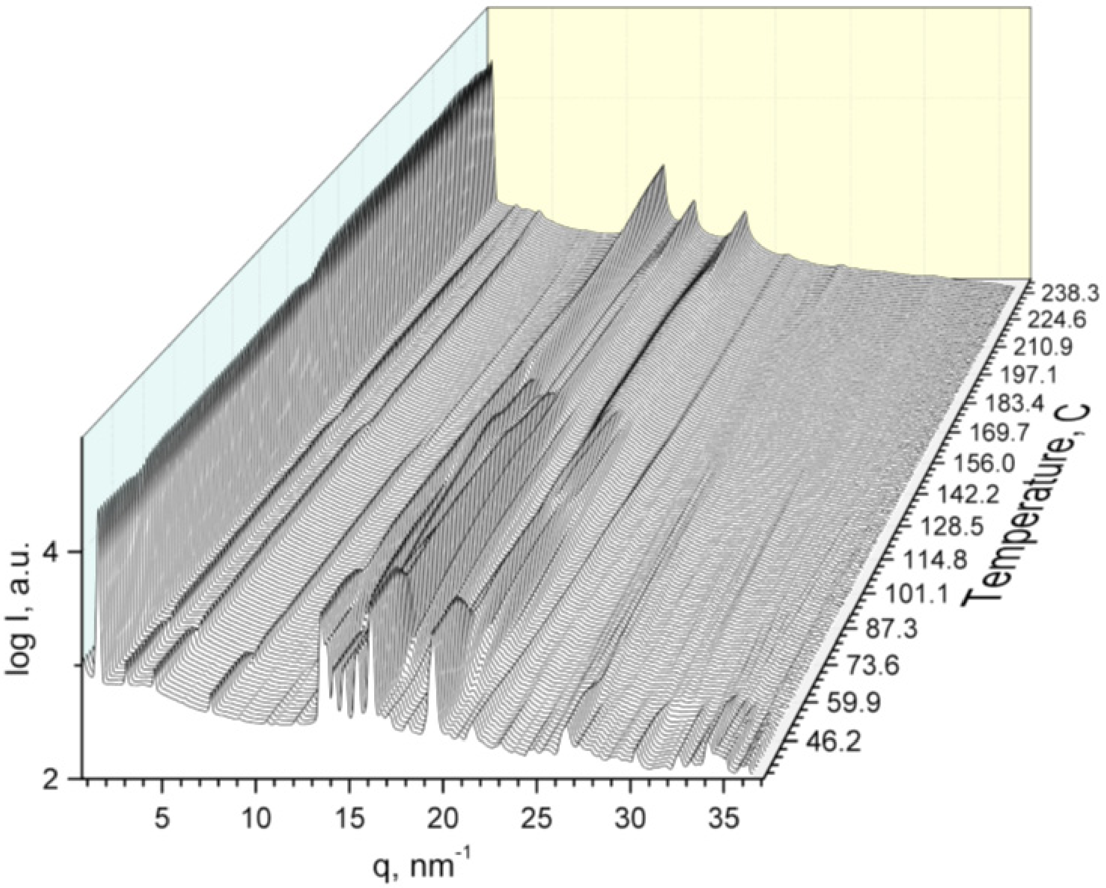

Figure S38. TWAXS plot of **BT-PD** at heating rate of 7 C/min

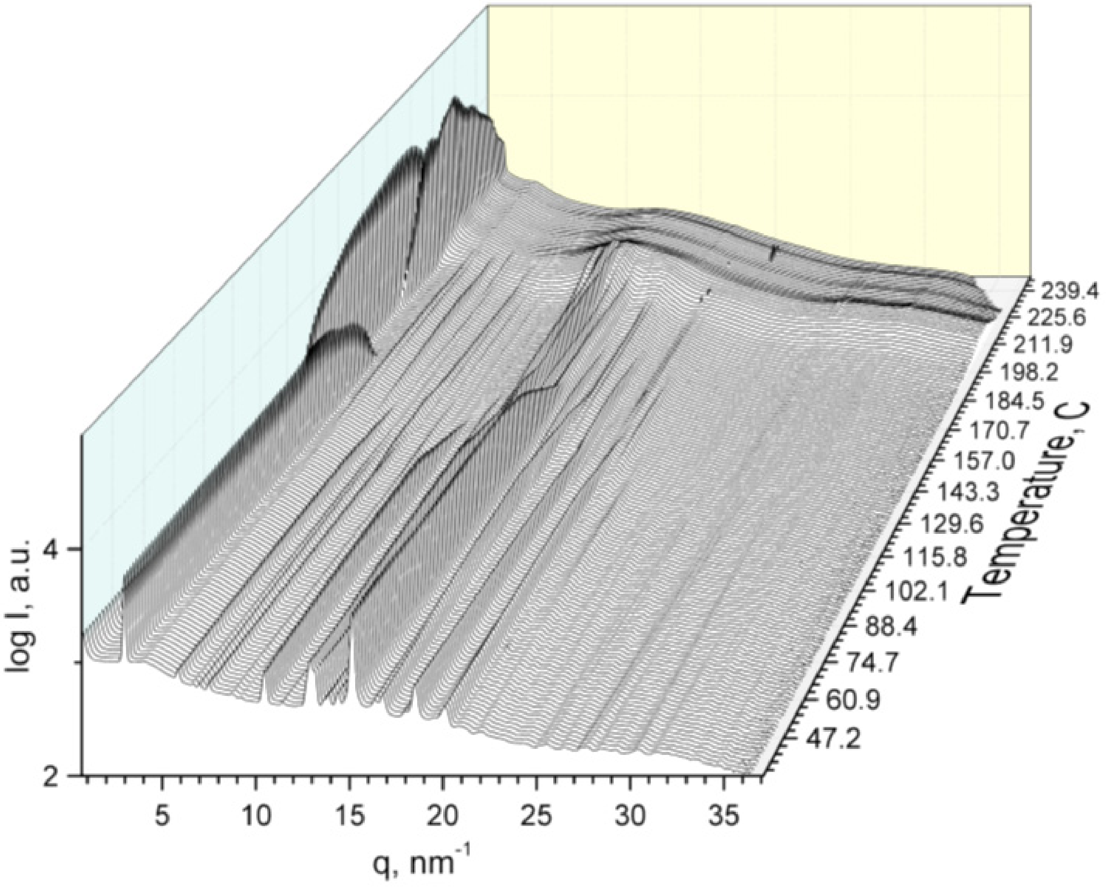

Figure S39. TWAXS plot of **ID-PCOD** at heating rate of 7 C/min

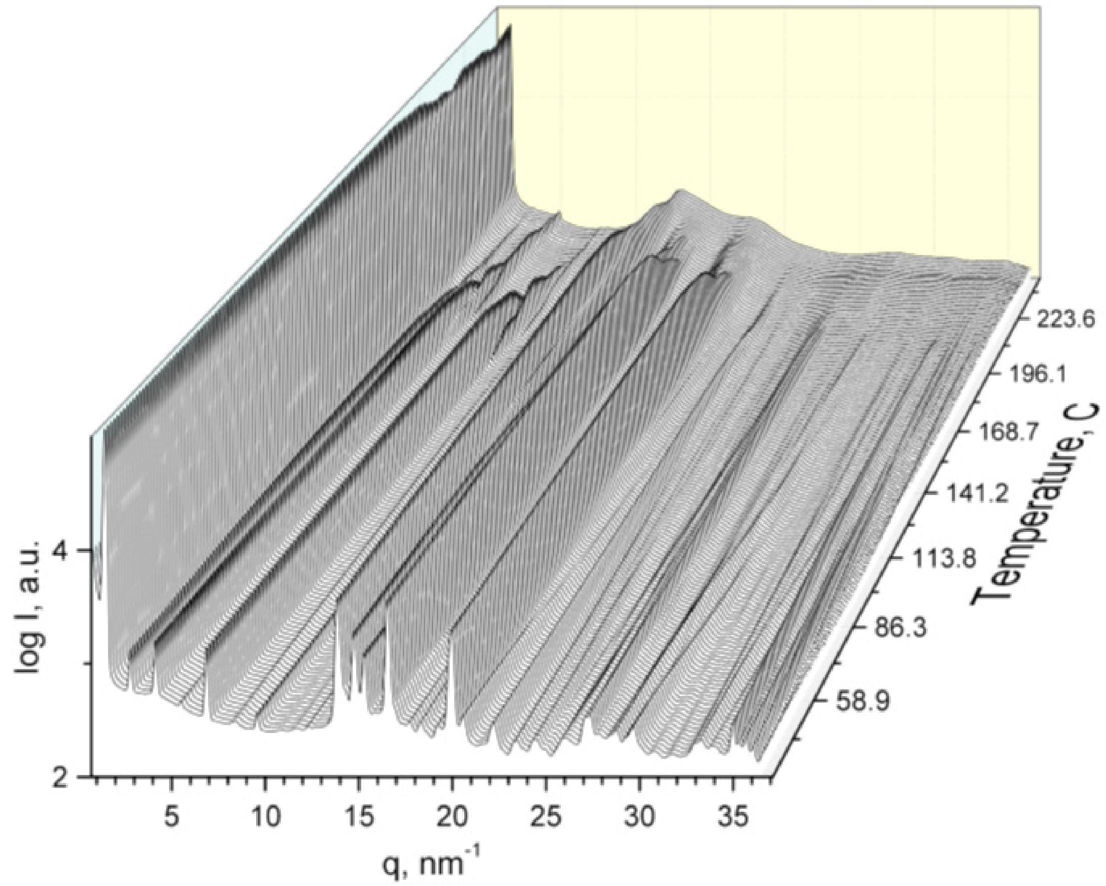

Figure S40. TWAXS plot of **BT-PCOD** at heating rate of 7 C/min

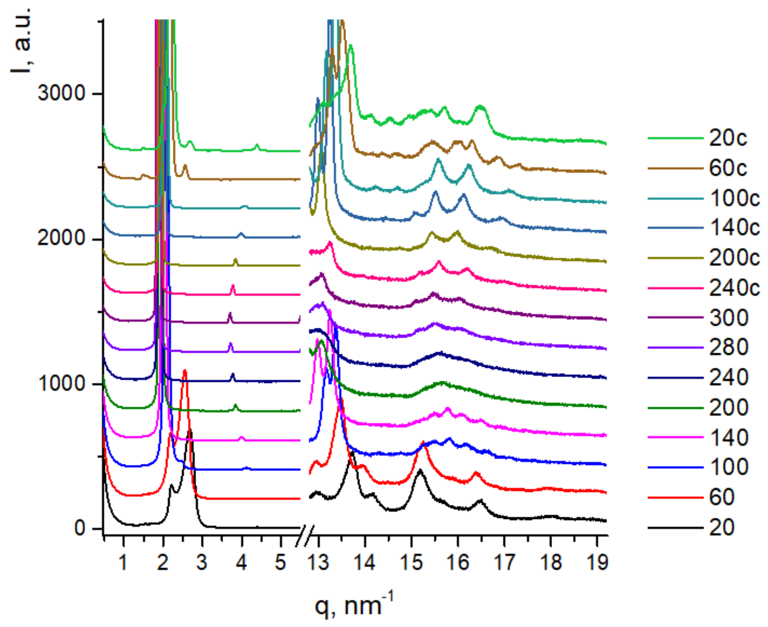

Figure S41. TWAXS plot of **ID-PD** at various temperature
